# Drift and Selection in a Recently Colonized Sea: The Genomic Basis of Adaptation to Low‐Salinity Waters in Baltic Harbour Porpoises

**DOI:** 10.1111/mec.70595

**Published:** 2026-10-16

**Authors:** Enrique Celemín, Kristín E. Tiedemann, Katja Havenstein, Harald Benke, Per Berggren, Michael Dähne, Judith Denkinger, Lisa Klemens, Christina Lockyer, Iwona Pawliczka, Anna Roos, Ursula Siebert, Linda Westphal, Ralph Tiedemann

**Affiliations:** ^1^ Institute of Biochemistry and Biology, Evolutionary Biology & Systematic Zoology University of Potsdam Potsdam Germany; ^2^ Department of Integrative Biology University of South Florida Tampa Florida USA; ^3^ Institute of Biology–Zoology Free University of Berlin Berlin Germany; ^4^ Deutsches Meeresmuseum Stralsund Germany; ^5^ School of Natural and Environmental Sciences Newcastle University Newcastle upon Tyne UK; ^6^ Institute of Biosciences University of Rostock Rostock Germany; ^7^ Bundesamt für Naturschutz Bonn Germany; ^8^ Colegio de Ciencias Biológicas y Ambientales Universidad San Francisco de Quito Quito Ecuador; ^9^ Age Dynamics Kongens Lyngby Denmark; ^10^ Hel Marine Station, Faculty of Oceanography and Geography University of Gdansk Hel Poland; ^11^ Swedish Museum of Natural History Stockholm Sweden; ^12^ Institute for Terrestrial and Aquatic Wildlife Research (ITAW) University of Veterinary Medicine Hannover Foundation Büsum Germany; ^13^ Leibniz‐Institute for Zoo and Wildlife Research Berlin Germany

**Keywords:** cetacean evolution, demographic history, local adaptation, osmoregulation

## Abstract

Understanding how demography and natural selection interact during colonization of novel environments is a central question in evolutionary biology. The Baltic Sea region combines one of the world's strongest salinity gradients with a well‐characterized postglacial history of less than ~12,000 years. The harbour porpoise ( *Phocoena phocoena* ) is distributed across this gradient as three populations—the North Sea, Belt Sea and Proper Baltic Sea populations. Using whole genome resequencing data from 59 individuals, we reconstructed the demographic history of Baltic colonization and identified genomic signatures of local adaptation. Demographic modelling supported a sequential colonization in which the Belt Sea population diverged first from an ancestral North Sea population, followed by an independent divergence of the Proper Baltic Sea population, with two equally supported timing hypotheses placing initial colonization either during the Yoldia Sea (~11,800 years ago) or the Baltic Sea period (~8000 years ago). The Proper Baltic Sea population showed drift‐dominated divergence consistent with a founder effect, while two discrete genomic islands of differentiation with selective sweep signatures were identified between North and Belt Sea populations, pointing to recent positive selection. Both regions contain osmoregulatory candidate genes, most notably SCNN1D, encoding part of the epithelial Na^+^ channels, a key regulator of sodium homeostasis. A non‐synonymous mutation in SCNN1D showed striking allele frequency divergence along the salinity gradient, confirmed across ~400 individuals. Together, these results reveal how genetic drift and divergent selection interact to drive rapid adaptation to a brackish environment, and identify SCNN1D as a compelling candidate for cetacean osmoregulatory adaptation.

## 1 Introduction

Understanding how species expand their ranges and adapt to novel environments is a long‐standing question in evolutionary biology (Savolainen et al. 2013). When colonization of new areas involves novel environmental conditions, new selective pressures may drive rapid local adaptation, particularly when selection acts on standing genetic variation already segregating in the ancestral population (Shah et al. 2020; Louis et al. 2021). However, the colonization of new habitats is rarely a purely selective process (Excoffier et al. 2009). Demographic events such as population bottlenecks and contractions, common during range expansions, can severely reduce effective population sizes (Ne) and erode the standing genetic variation upon which selection depends (Kardos et al. 2021). This tension between selection and demographic constraints is central to understanding population diversification: while environmental heterogeneity across a species' range can promote local adaptation by exposing populations to divergent selective pressures, genetic drift associated with demographic history may counteract or obscure these adaptive signals (Forester et al. 2016; Walter et al. 2016). Disentangling these processes therefore requires studying systems where populations have recently colonized environmentally distinct habitats, allowing us to reconstruct both the demographic history of colonization and the genomic signatures of rapid adaptation (Lotterhos and Whitlock 2014; Hoban et al. 2016).

The postglacial Baltic Sea represents an ideal such system: a geologically young, environmentally dynamic basin that went through two consecutive lake‐sea cycles after the last glacial maximum (LGM) before the modern brackish water‐filled Baltic Sea emerged ~8500 years before present (yBP) (Svendsen et al. 2004). These cycles included the short‐lived, partially brackish Yoldia Sea (~11,700–10,700 yBP), formed when glacial retreat opened a marine connection through central Sweden, and the subsequent freshwater Ancylus Lake (~10,700–9000 yBP), established when isostatic rebound closed that connection and isolated the basin from the ocean (Figure S1) (Björck 1995). The modern Baltic Sea is an extreme marine environment characterized by low winter temperatures and one of the strongest salinity gradients in the world, extending from the North Sea to the inner Baltic Sea (Feistel et al. 2010). The Baltic Sea presents shallow underwater ridges ranging from the North Sea through Skagerrak, Kattegat, Belt Seas to the entrance of the proper Baltic Sea, which presumably limit dispersal and gene flow (Johannesson and André 2006) in the area. Due to these remarkable conditions, Baltic populations of several marine organisms are genetically distinct from North Sea conspecifics (Wennerström et al. 2013; Sjöqvist et al. 2015; Momigliano et al. 2021; Geburzi et al. 2022), which is also the case of the North Atlantic harbour porpoise ( *Phocoena phocoena phocoena* ), the only resident cetacean species of the Baltic Sea.

The North Atlantic harbour porpoise inhabits greatly diverse coastal seascapes, broadly varying in depth, salinity and temperature, with a continuous distribution extending from the French Biscayan waters to the Barents Sea, and from the Norwegian Sea to the western North Atlantic coast of Canada and the United States (Gaskin 1992; Read et al. 2025). Although genetic and genomic data suggest that populations on both sides of the Atlantic belong to a single panmictic unit (Ben Chehida et al. 2021; Celemín et al. 2025), substantial population structure has been documented at finer geographic scales (Alfonsi et al. 2012; Luna et al. 2012; Quintela et al. 2020). In particular, passive acoustic (Carlén et al. 2018), telemetry (Sveegaard et al. 2015), morphological (Börjesson and Berggren 1997; Galatius et al. 2012), and genetic data (Tiedemann et al. 1996; Wiemann et al. 2010; Lah et al. 2016; Autenrieth et al. 2024; Celemín et al. 2025) consistently point to the existence of distinct ecotypes or populations in the peripheral waters of the Baltic Sea. Three such ecotypes or populations have been described based on a combination of genetic, morphological and seasonal migration patterns delineating putative population borders (Gallus et al. 2012; Sveegaard et al. 2015; Carlén et al. 2018): one in the North Sea, Skagerrak and northern Kattegat (North Sea population, NOS), one in the southern Kattegat, the Sound, the Belt Seas and western Baltic (Belt Sea population, BES), and one in the inner Baltic Sea (Proper Baltic Sea population, PBS). While spatial overlap between populations has been reported (Celemín et al. 2025), their management boundaries are defined by geographic segregation during the reproductive season (Figure 1A; Sveegaard et al. 2015; Carlén et al. 2018).

**FIGURE 1:**
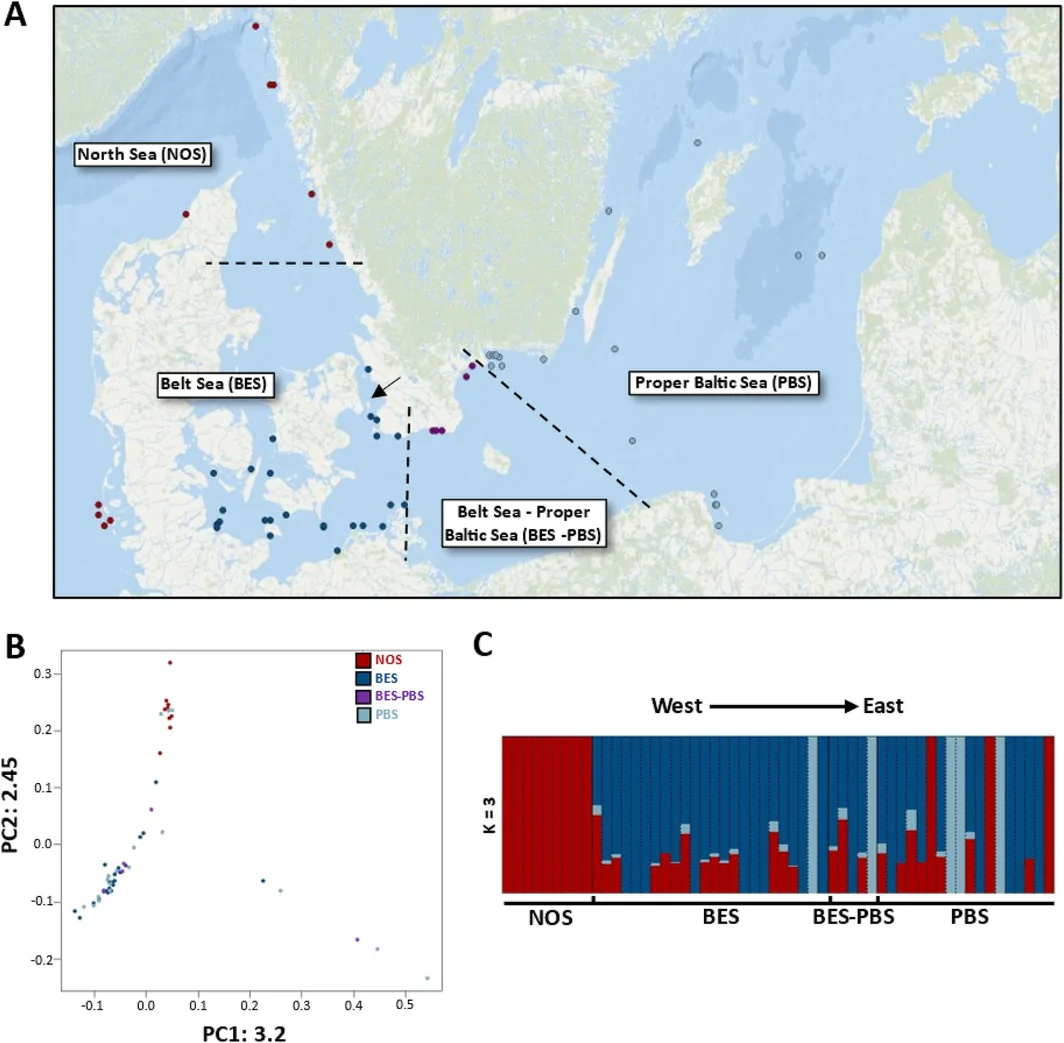
Map and population structure of Harbour porpoises from the Baltic region demonstrating the existence of three populations: North Sea, Belt Sea and Proper Baltic Sea. (A) The 56 Harbour porpoise samples used in the WGS project. Samples are coloured according to location: North Sea—Skagerrak (NOS) red, Belt Sea (BES) dark blue, Belt Sea—Proper Baltic Sea transition zone (BES‐PBS) purple, Proper Baltic Sea (PBS) light blue. The dotted lines represent the May to October population boundaries identify by Carlén et al. (2018) and Sveegaard et al. (2015). The arrow depicts the Öresund strait. (B) Principal Component Analysis (PCA) showing the first and second PCs. The colours represent sampling location. (C) Admixture Analysis, only K = 3 is shown. Colours represent genetic clusters: Red North Sea population, dark blue Belt Sea population, light blue Proper Baltic Sea population.

Harbour porpoise population borders in the Baltic coincide with the marked salinity gradient, ranging from ~34 to 20 practical salinity units (psu) in the Skagerrak (NOS population), ~30–10 psu in the Belt Seas (BES populations), to ~10–0 psu in the innermost parts of the Baltic (PBS population) (Feistel et al. 2010). A previous genomic study found that salinity is the major driver of genomic variation in North Atlantic Harbour porpoises, suggesting that Baltic populations have undergone local adaptation to low‐salinity conditions (Celemín et al. 2025). Occupation of low‐salinity environments requires significant osmoregulatory adaptations to maintain stable ion and water homeostasis, particularly the renal reabsorption of sodium (Na^+^), one of the most critical ions for vertebrate extracellular fluid balance (Wichmann and Althaus 2020). A key molecular mechanism underlying this process are the epithelial sodium channels (ENaCs), which mediate selective Na^+^ influx across the apical membrane of epithelial cells in the kidney's distal nephron and are essential for fine‐tuned sodium homeostasis in tetrapod vertebrates (Rossier et al. 2015). Although recent studies have begun to reveal the molecular footprints of cetacean adaptation to freshwater environments (Zhou et al. 2018; Ramos et al. 2023), these studies have largely focused on deeply diverged species or populations and examined only a limited set of candidate osmoregulatory genes, leaving the genomic basis of rapid brackish water adaptation poorly understood.

Here we addressed this gap by investigating the diversification of harbour porpoises in the Baltic Sea region and unravelling the molecular footprints of their colonization of a brackish environment. Using whole genome sequencing (WGS) data, we first identified the three populations occurring in the Baltic region and reconstructed their demographic history to determine the sequence and timing of Baltic colonization events in the context of the postglacial history of the basin. We then compared their genomic landscape of diversity and divergence, looked for outlier loci and signatures of selective sweeps, and examined the genotypes of putative adaptive genetic variants. We further developed a reduced SNP panel that clearly separates the three populations and used it in a larger sample set to study the geographical distribution of a relevant intragenic SNP located in an osmoregulatory gene that encodes part of the epithelial Na^+^ channel. Given the strong salinity gradient across the Baltic region and the demographic bottlenecks expected during colonization of a novel, low‐salinity environment, we expect to find highly divergent genomic regions associated with osmoregulatory candidate genes between populations distributed in the high (NOS) and low (BES, PBS) salinity areas, and that the demographic history of Baltic populations reflects the successive postglacial connections between the Baltic basin and the North Sea.

## 2 Material and Methods

### 2.1 Sampling, DNA Extraction and WGS Sequencing

We used 59 whole genomes of harbour porpoises sampled in Baltic waters (Figure 1A, Table S1), of which 51 were generated by Celemín et al. (2025) (PRJNA997110) and 8 were newly produced during the present project. To validate the SNP panel and characterize population structure at a larger geographic scale, we additionally genotyped 470 tissue samples (436 unique individuals and 34 replicates; Table S2). Sampling was opportunistic, conducted on dead bycaught or stranded individuals; no live porpoises were targeted. DNA was extracted from skin or muscle tissue, and whole genome libraries were prepared and sequenced following the protocol described in Celemín et al. (2025).

### 2.2 Reference Genome Alignment, WGS Reads Processing and SNP Calling

Since cetacean chromosomal arrangement is widely conserved (Yuan et al. 2021), we used the chromosome‐level reference genome of the closely related vaquita ( *Phocoena sinus* ; Morin et al. 2021, accession no. GCF_008692025.1) to assign scaffolds of the Belt Sea harbour porpoise genome (Autenrieth et al. 2018, accession no. GCA_003071005.2) to pseudochromosomes. Synteny alignments were performed with SatsumaSynteny2 (Grabherr et al. 2010), retaining only scaffold alignments with identity ≥ 0.6 and at least 50% of scaffold length mapped to a single chromosome, following de Greef et al. (2022). Of the 160 harbour porpoise scaffolds > 1 Mb (representing 97.69% of the genome), 126 aligned confidently to a vaquita autosome, 27 to chromosome X, and the remaining 7 were classified as unplaced, yielding 22 pseudochromosomes with chromosomal coverage ranging from 70% to 99% of each vaquita chromosome (Figure S2).

WGS reads were processed following Celemín et al. (2025); the full bioinformatic pipeline is summarized in Figure S3. Briefly, low‐quality reads were filtered with Fastp (v.0.23.2; Chen, Marschall, et al. 2018; Chen, Zhou, et al. 2018), mapped to the reference genome with the Bwa mem (v.0.7.17; Li and Durbin 2009), and Picard tools (v.2.27.2) was used to add read groups and to remove duplicates from the aligned reads. Alignment files were further processed by removing interspersed repeats identified with RepeatMasker (v.4.1.2; Smit et al. 2013), removing scaffolds mapped to chromosome X, and excluding regions with coverage below one‐third or above twice the mean. Two individuals identified as first‐degree relatives and one with coverage below 5X (3.5X) were discarded, yielding a final dataset of 56 individuals with a mean sequencing depth of 10.4X (Table S1).

Genotype likelihoods were estimated with ANGSD (v.10.2.0; Korneliussen et al. 2014) using the Samtools model (GL 1), retaining SNPs with a minimum minor allele frequency (MAF) of 0.05, data in at least 75% of individuals and, a SNP *p*‐value< 1e^−6^, yielding 7,485,436 high‐quality SNPs. Genotypes were called with Bcftools (v.1.11; Danecek et al. 2021) mpileup and call, using the multiallelic and rare‐variant calling option ‐m, retaining biallelic SNPs with MAF > 0.05, minimum site and mapping quality of 30, and data in at least 95% of individuals, resulting in a final set of 6,277,152 high‐quality SNPs. Additionally, a VCF including invariant sites was generated with Bcftools, retaining biallelic sites with a maximum of 5% missing data per site, for use in downstream diversity and divergence analyses.

### 2.3 WGS Population Structure Analysis

Genomic population structure was assessed using principal component analysis (PCA) implemented in PCAngsd (Meisner and Albrechtsen 2018) and admixture analysis implemented in NGSAdmix (Skotte et al. 2013). First, since genomic clustering can be affected by the presence of first‐degree relatives, we calculated pairwise relatedness statistics with the software *NgsRelate* (v.2.1; Hanghøj et al. 2019) to remove any potential first‐degree relatives that could bias our analysis. Second, unlinked genotype likelihoods were identified with ngsLD (Fox et al. 2019), treating SNPs on the same scaffold within 20 Kb of each other as potentially in linkage disequilibrium (LD) and applying a minimum LD weight threshold of 0.5. For the admixture analysis, 50 independent runs were performed for each value of K (ranging from 2 to 3), with a minimum convergence tolerance of 1 × 10^−10^ and a minimum likelihood ratio of 1 × 10^−6^. The best‐supported K was determined using the ΔK method (Evanno et al. 2005). For downstream analyses, individuals were assigned to a population if they showed ≥ 75% ancestry to any single cluster under K = 3; individuals with < 75% ancestry to any cluster were considered admixed and excluded from population‐level analyses.

### 2.4 Demographic History Inference

Demographic history inference was performed using genomic data from 15 harbour porpoise specimens selected to maximize sequencing coverage and to include the same number of individuals per population (five from each population: NOS, BES and PBS: cf. Table S1). The previously generated VCF file was further filtered to remove missing data and putative adaptive SNPs identified with Pcadapt (see below) to ensure demographic models were based exclusively on neutral SNPs. Then, the observed site frequency spectrum (SFS) was generated using easySFS (Overcast 2023; Gutenkunst et al. 2009), and demographic modelling was performed with fastsimcoal2 v2.7.0 (Excoffier et al. 2021).

Thirteen scenarios of increasing complexity, representing different models of population divergence and connectivity, were tested (see Figure S4, Table S3 for details). Scenario P1 assumed a simultaneous split of the three populations after the formation of the Baltic Sea (~8500 years before present (yBP)) without subsequent gene flow (1 splitting time estimated). The P2 scenarios assumed sequential splits, in which one ancestral population first divided into two, and later gave rise to a third population, without gene flow (2 splitting times estimated). The timing of these splits was either restricted to the Baltic Sea period (< 8500 yBP; scenarios P2a and P2b) or allowed to extend back into the Yoldia Sea period (< 12,000 yBP; scenarios P2c, P2d, and P2e). The topology (i.e., the order of divergence among the three populations) was unconstrained in scenarios P2a and P2c. Scenario P2b assumed that PBS and BES diverged from NOS shortly after Baltic Sea formation (~8500–6000 yBP), followed by a split between BES and PBS (~6000–5500 yBP). Scenario P2d assumed the same topology but placed the initial isolation of Baltic porpoises from NOS during the Yoldia Sea period (~12,000–10,000 yBP), with the subsequent divergence of BES from PBS occurring after Baltic Sea formation (~8500 yBP). Finally, scenario P2e assumed that PBS was the first to diverge, separating from the ancestral population during the Yoldia Sea period (~12,000–10,000 yBP), with BES subsequently originating from NOS after Baltic Sea formation (~8500 yBP). The P6 and P8 scenarios resemble their respective P2 counterparts, but additionally incorporate asymmetric gene flow: among adjacent populations only in the P6 scenarios (2 splitting times and 4 migration rates estimated), or among all populations in the P8 scenarios (2 splitting times and 6 migration rates estimated).

We applied a mutation rate of 2.56 × 10^−8^ per site per generation (Louis et al. 2021; derived from Yim et al. 2014) and converted generations to years using the harbour porpoise generation time of 11.9 years (Taylor et al. 2007). This mutation rate was adopted for consistency with our previous study on the evolutionary history of *P. phocoena* subspecies and populations (Celemín et al. 2025), allowing direct comparability of demographic estimates across analyses. Given the assumption of constant mutation rates across time, we ran additional models for the two preferred scenarios (see below) using two alternative mutation rates to assess their influence on the inferred demographic trajectories: the rate estimated for vaquita, 1.08 × 10^−8^ (Morin et al. 2021), and the Yim et al. (2014) rate rescaled using the harbour porpoise generation time, 1.44 × 10^−8^. For scenarios incorporating gene flow, the proportion of migrants during population splits was set to 100% and population growth rates were fixed at zero. Migration rates were allowed to vary between 0.000001 and 0.001. Uniform prior distributions were applied to all parameters except migration rates. Ancestral deme sizes were bounded between 1000 and 2 × 10^6^ individuals, while post‐split deme sizes were constrained to 0.01–100 times the ancestral deme size. NOS was designated as the ancestral population in all scenarios.

For each scenario, 100 independent likelihood maximization runs were performed using expectation‐conditional maximization (ECM) optimization over 75 cycles with random starting parameter sets. Likelihoods were approximated from expected SFS values derived from 600,000 coalescent simulations per cycle. The parameter set yielding the highest likelihood across all 100 runs was then selected as the starting point for final inference, in which likelihood distributions were computed from 100 runs of 1000,000 coalescent simulations per model. Then, scenarios were compared using a strictly orthogonal scheme (Figure S5 and Table S3) to maintain an experiment wise error rate of *α* = 0.01 without further correction (Sokal and Rohlf 1995). Model comparisons were based on two criteria: non‐overlap of likelihood ranges across the 100 iterations, and biological plausibility of the inferred population parameters.

### 2.5 Population Pairwise Nucleotide Divergence and Diversity

To characterize the genomic landscape of divergence and diversity across the three populations, we estimated pairwise relative divergence (Fst), absolute divergence (dxy) and within‐population nucleotide diversity (*π*) in non‐overlapping 20 Kb windows across the genome. Prior to Fst estimation, LD decay was assessed along the longest scaffold using PopLDdecay (Zhang et al. 2018), and a subset of unlinked genotypes was identified with PLINK (v.1.9; Purcell et al. 2007) using the ‐‐indep‐pairwise option (20 Kb window size, 20 Kb step size, *r* ^2^ threshold of 0.5). Population pairwise‐weighted Fst values were then calculated for each population comparison (NOS‐BES, NOS‐PBS and BES‐PBS) with VCFtools (v.0.1.16; Danecek et al. 2011) using the ‐‐weir‐fst‐pop option. Results were visualized as Manhattan plots along the 21 pseudochromosomes (unplaced scaffolds were merged in an artificial “unplaced” chromosome) using the R package qqman (Turner 2018), with the top 0.05% of windows highlighted as candidate high‐differentiation regions. Genome‐wide Fst values per population comparison were additionally summarized in a heatmap.

Nucleotide diversity (*π*) and absolute divergence (dxy) were computed with Pixy (v.0.95.02; Korunes and Samuk 2021) on the VCF including invariant sites, using the same 20 Kb non‐overlapping window approach. To disentangle the relative contributions of selection and demographic history to the genomic landscape, we used linear models to examine pairwise relationships between Fst, *π*, and dxy estimates. As a proxy for effective population size (Ne) and the relative influence of genetic drift across populations, Watterson's θ and Tajima's D were estimated with ANGSD. For each inferred population, a folded site frequency spectrum (SFS) was first computed independently, and both statistics were then estimated in non‐overlapping 20 Kb windows using the same window and step size as above.

### 2.6 Selection Analysis: Outlier SNPs and Selective Sweeps

To identify candidate SNPs and genomic regions under local adaptation, we employed two complementary approaches: differentiation outlier scans and selective sweep detection. Three outlier scan methods were performed to identify SNPs with excessive divergence relative to the genomic background, which may reflect reduced gene flow due to divergent selection (Giakoumis et al. 2023). The first was a multivariate, population‐assignment‐free scan implemented in the R package Pcadapt (Privé et al. 2020), using three principal components (K) selected based on the population structure analysis and the scree plot decay rate to minimize genomic inflation. Outlier SNPs were identified using *q*‐values computed with the R package *q*‐value, applying a false discovery rate (FDR) threshold of 0.1. The second scan was based on per‐site Fst values computed with VCFtools ‐‐weir‐fst‐pop on the subset of unlinked genotypes for each population comparison (NOS‐BES, NOS‐PBS and BES‐PBS), retaining the top 1% of SNPs per comparison as candidates. The third scan was a genotype‐environment association (GEA) analysis using Latent Factor Mixed Models (LFMM) implemented in the R package lfmm (Caye et al. 2019), with mean sea surface salinity (mSSS) as the environmental variable, previously identified as the major driver of genomic variation in North Atlantic Harbour porpoises (Celemín et al. 2025). Salinity values were downloaded from BioOracle (v.2.2; Tyberghein et al. 2012; Assis et al. 2018) and candidate SNPs were identified at a FDR of 0.1 (*q*‐value < 0.01). Only SNPs identified by all three methods were retained as high‐confidence putative adaptive SNPs for downstream analyses.

When a strongly advantageous mutation rises to fixation, linked neutral variation nearby is reduced through hitchhiking, leaving a detectable signature of recent positive selection known as a selective sweep. To identify such signatures across Baltic Harbour porpoise populations, SNPs with > 5% missingness were first removed and remaining SNPs were imputed and phased with BEAGLE (v.5.2; Browning et al. 2018, 2021) using default imputation parameters (1600 model states, 6 cM haplotype segments, 0.1 cM step size, 7 consecutive steps) and 20 phasing iterations (maximum 3 burnin iterations, 280 model states). Cross‐population extended haplotype homozygosity (XP‐EHH) statistics (Sabeti et al. 2007) were computed for each population comparison using the ies2xpehh function in the R package rehh (Gautier and Vitalis 2012). Candidate sweep regions were identified with the calc_candidate_regions function using a *q*‐value threshold of 0.01 (−log_10_(0.01)), a window size of 100 Kb overlapping by 10 Kb, and a minimum of two significant markers per region. Significant selective sweeps were defined as SNPs falling within a candidate region and with *q*‐value < 0.01. Positive XP‐EHH values indicate greater haplotype homozygosity in the first population, consistent with recent positive selection in that population, while negative values implying possible putative positive selection in the second population (Sabeti et al. 2007; Gautier and Vitalis 2012).

### 2.7 Candidate Genes Identification and Gene Ontology Analysis

Candidate genes were identified by mapping putative adaptive SNPs to the vaquita genome annotation. For each SNP, a 601 bp sequence (300 bp flanking region on each side) was extracted from the Harbour porpoise genome using BEDtools (v.2.30; Quinlan and Hall 2010) and used as a query in a BLASTN search against the vaquita genome, retaining only hits with *e*‐value ≤ 1 × 10^−50^ and sequence identity ≥ 95%. The best hit per SNP was retained and genes located within 20 Kb of each putative adaptive SNP were retrieved from the vaquita reference genome annotation using MAGMA (de Leeuw et al. 2015). Gene Ontology (GO) enrichment analysis was performed on the resulting candidate gene set using DAVID (Huang et al. 2009; Sherman et al. 2022), with significance assessed using Fisher's exact test at *p* < 0.01.

### 2.8 SNP Panel Selection and Evaluation

We developed a reduced SNP panel with two main objectives: to enable cost‐effective population assignment of opportunistically sampled individuals across the Baltic region at a larger geographic scale, and to characterize the geographic distribution of the putative adaptive variant within SCNN1D across Baltic waters. To maximize discriminatory power between the three Baltic populations, SNPs were ranked by pairwise Fst and the 64 SNPs with the highest Fst were selected for each population comparison (NOS‐BES, NOS‐PBS and BES‐PBS), following procedures used in similar studies (Jenkins et al. 2019; Bonanomi et al. 2015; Therkildsen et al. 2013). SNPs already selected in another comparison or with a neighbouring SNP within 200 bp were replaced by the next highest‐ranking SNP. Additionally, the putative adaptive non‐synonymous SNP within SCNN1D was included regardless of its Fst rank, given its functional relevance to osmoregulatory adaptation. This yielded a candidate panel of 192 SNPs (Table S4). For each SNP, a 401 bp sequence (200 bp flanking region on each side) was extracted from the Harbour porpoise genome using BEDtools (Quinlan and Hall 2010) getfasta and submitted to the Fluidigm D3 Assay Design portal for primer design.

The discriminatory power of the panel was evaluated by reassigning the WGR baseline individuals (those assigned to a population under K3 with > 75% ancestry) back to their source population using three approaches. PCA was performed with the R package adegenet (Jombart and Ahmed 2011) and admixture analysis with STRUCTURE (v.2.3.4; Pritchard et al. 2000), run with 10^6^ MCMC repetitions, a burnin of 10^5^ and K3. Assignment accuracy was further assessed using the R package assignPOP (v.1.2.4; Chen, Marschall, et al. 2018; Chen, Zhou, et al. 2018), which implements a resampling cross‐validation workflow in which individuals are divided into training and test sets and assignment tests are repeated across different proportions of SNPs (top 10%, 25%, 50% and all) and training set sizes (50%, 70% and 90% of individuals) to estimate membership probabilities. Similar evaluation frameworks have been applied in comparable SNP panel studies (Sun et al. 2023; O'Donnell and Sullivan 2021; Jenkins et al. 2019; Wringe et al. 2018).

### 2.9 SNP Panel Genotyping and Analysis

SNP genotyping was carried out with Fluidigm SNP type assays using a Juno controller and a EP1 reader, following the manufacturer's protocol and the method described by Seeb et al. (2009). Fluidigm SNP type assays perform two consecutive amplification steps: the first pair of primers amplifies the SNP locus, while the second amplifies specific alleles and binds allele‐specific fluorescent tags that are measured to determine genotype (Carroll et al. 2018). This technology presents very low error rates (DeWoody et al. 2017; Nussberger et al. 2014) and has been successfully applied to a broad range of sample types and taxa, including plants (Nygaard et al. 2022; Finch et al. 2020), aquatic (Östergren et al. 2021; Therkildsen et al. 2013) and terrestrial animals (von Thaden et al. 2020; Doyle et al. 2016), and non‐invasive (von Thaden et al. 2017; Kraus et al. 2014), tissue (Jenkins et al. 2019; Förster et al. 2018) and otolith samples (Bonanomi et al. 2015, 2016).

We used ten 96 × 96 GT Integrated Fluidic Circuit (IFC) chips—which perform an additional preamplification reaction for improved genotyping accuracy and simultaneously genotype 96 SNPs across 96 samples—to genotype the 192 selected SNPs across 480 samples (436 unique individuals, 34 replicates and 10 negative controls; Table S2), including all individuals for which WGS data were available to enable WGS‐Fluidigm concordance assessment. Samples were diluted to ~20 ng/μL and run for 57 amplification cycles, including 14 preamplification cycles. Genotypes were called using the Fluidigm genotyping analysis software (v.4.5.1) with the SNPtype normalization method. Samples and SNPs with a call rate < 75% or auto confidence < 90% were excluded as unreliable. For each remaining SNP, genotyping error rates were estimated from replicate pairs (both WGS‐Fluidigm and Fluidigm‐Fluidigm comparisons) as the proportion of replicates yielding discordant genotypes, and SNPs with an error rate > 25% in either comparison were discarded, yielding a final set of 149 reliable SNPs.

Population assignment of all genotyped individuals was performed using the assignPOP function assign.X with a naïve Bayes model, assigning each individual to one of the three Baltic populations. Population allele frequencies for the putative adaptive SNP within SCNN1D were then calculated for each assigned population and plotted geographically to characterize the spatial distribution of this osmoregulatory variant across Baltic waters.

## 3 Results

### 3.1 Genetic Structure

To characterize the genomic diversity and population structure of harbour porpoises across the Baltic region, we sequenced 59 individuals to a mean depth of 10.4X, of which 56 passed quality and relatedness filters (Table S1; see Methods). Genotype likelihood estimation across autosomal scaffolds yielded 7,485,436 high‐quality SNPs, of which ~1.7 million were unlinked. The population structure analysis (Figure 1) confirmed the existence of three harbour porpoise populations occurring in the Baltic. The PCA (Figure 1B) revealed two major axes of differentiation: PC1 (3.2% of variance) separated five individuals (3 from PBS region, 1 from BES‐PBS and the second‐most eastern BES individual) from the rest of the samples, while PC2 (2.45% of variance) broadly divided Baltic porpoises from North Sea individuals, with the exception of three PBS porpoises clustering with the NOS group. Admixture results were consistent with the PCA: K2 separated PBS from the remaining individuals, K3 further subdivided NOS from BES porpoises, and the Delta K method supported K3 as the best‐supported number of populations (Figure S6). For downstream analysis, individuals were assigned to a population if ancestry membership was ≥ 75% under K3 (Figure S7), yielding 12 porpoises assigned to the North Sea population (red cluster), 30 assigned to the BES population (dark blue cluster), and 5 assigned to the Proper Baltic Sea population (light blue cluster). Nine admixed individuals between NOS and BES were excluded from downstream analyses.

### 3.2 Demographic Inference

To reconstruct the colonization history of Baltic harbour porpoises, we modelled 13 demographic scenarios (Figure S4) of increasing complexity. We compared models of simultaneous and sequential population splits with and without gene flow, and tested whether colonization timing was consistent with the Baltic Sea or the earlier Yoldia Sea period (see Methods; Figure S5; Table S3). The simplest simulated scenario—a simultaneous split of one ancestral population into the three populations NOS, BES, and PBS (scenario P1; cf. Figure S4)—was rejected in favour of scenarios involving two sequential splits, which yielded considerably higher likelihoods (Figure S8). Notably, simulations with unconstrained topology (scenarios P2a and P2c; cf. Figure S4) supported a scenario in which BES diverged first, followed by a later split of PBS from the NOS population. When simulations were constrained to a closer relationship between the two Baltic populations (scenarios P2b and P2d; cf. Figure S4) or to an early divergence of PBS (scenario P2e; cf. Figure S4), likelihoods decreased (Figure S8). Incorporating gene flow between adjacent populations substantially increased the likelihoods of the respective models (compare scenarios P6x with their P2x counterparts; Figure S8), whereas allowing gene flow among all populations did not yield meaningful further improvements (compare scenarios 6a and 6c with 8a and 8c, respectively; Figure S8). Further, they yielded biologically implausible population size estimates, with 98% of all porpoises assigned to BES (Table S5). In summary, these simulations support a scenario in which the BES population first diverged from an ancestral NOS population. Subsequently, the PBS population also originated from NOS, rather than from the geographically adjacent BES population (Figure 2; Table S3).

**FIGURE 2:**
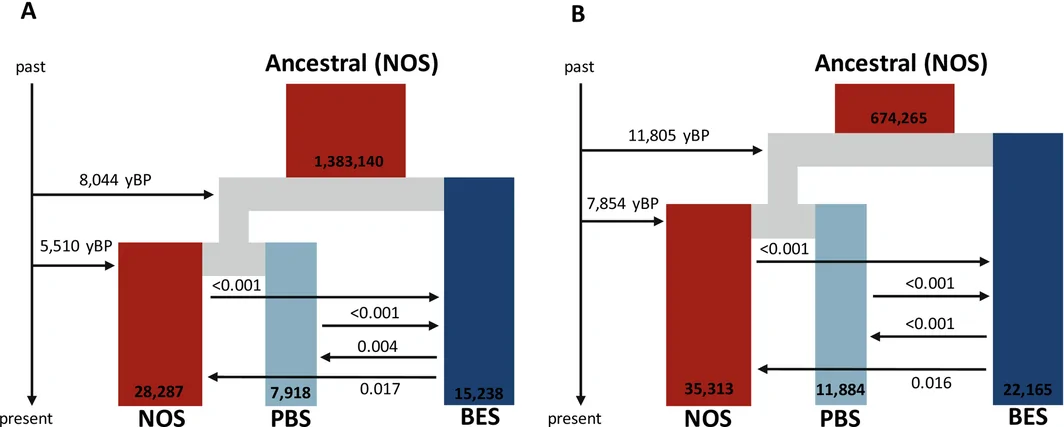
Inferred demographic history of harbour porpoise populations under the best‐supported scenarios. Graphical representation of the maximum likelihood parameter estimates from *fastsimcoal2* for scenarios P6a (A) and P6c (B). Bar width is proportional to the inferred effective population size (number of individuals shown in bold within each bar) for the ancestral NOS population (red), and the present‐day NOS (red), Belt Sea (BES; dark blue), and Proper Baltic Sea (PBS; light blue) populations. Arrows indicate asymmetric migration rates (proportion of migrants per generation) between adjacent populations, with values shown alongside each arrow. Divergence times are given in years before present (yBP) and indicated by horizontal lines. In scenario P6a, BES diverged from the ancestral NOS population at 8044 yBP, followed by the divergence of PBS from NOS at 5510 yBP, both within the Baltic Sea period. In scenario P6c, the first divergence event is placed during the Yoldia Sea period (11,805 yBP), with the subsequent divergence of PBS from NOS occurring during the Baltic Sea period (7854 yBP).

Regarding the timing of colonization, restricting the colonization after the Baltic Sea emergence (scenarios P2a, P6a, P8a) yielded similar likelihoods as their counterparts in which colonization during the Yoldia Sea period was allowed (scenarios P2c, P6c, P8c) (Figure S8). In the Baltic Sea scenarios, the BES divergence was estimated to have occurred between 7902 and 8044 years before present (yBP), with the subsequent divergence of PBS from NOS between 4296 and 5510 yBP (Table S3). In the Yoldia Sea scenarios, the BES divergence was between 10,103 and 11,805 yBP and the subsequent PBS divergence from NOS between 4891 and 7854 yBP (Table S3). Notably, in the best‐supported Yoldia Sea scenario (P6c; Figure 2B), the first split is estimated to fall within the Yoldia Sea period (~11,805 yBP) and the second during after the Baltic Sea emergence (~7854 yBP)—both intervals corresponding to periods during which the Baltic and North Seas were connected.

### 3.3 Genomic Landscape of Divergence and Diversity

To characterize patterns of genomic divergence and diversity across the three populations, we computed pairwise Fst, absolute divergence (dxy) and within‐population nucleotide diversity (π) in 109,703 non‐overlapping 20 Kb windows covering the genome. Windowed Fst estimates revealed 56 highly divergent genomic regions (exceeding the 99.9th percentile) across the three pairwise comparisons (Figure 3A,B; Figure S9). Strikingly, the NOS‐BES comparison presented two clear high‐Fst peaks located in pseudoautosomes 1 and 15 (Figure 3A), with no equivalent elevated regions detected in the BES‐PBS or NOS‐PBS comparisons (Figure 3B; Figure S9). Despite this, genome‐wide Fst was lower in the NOS‐BES comparison (0.013) than in BES‐PBS and NOS‐PBs (0.034 and 0.040, respectively; Figure 3E), while absolute divergence (dxy) was similar across all three comparisons (NOS‐BES: 0.00192; NOS‐PBS: 0.00190; BES‐PBS: 0.00188) and heterogeneously distributed along the genome (Figure 3A,B; Figure S9).

**FIGURE 3:**
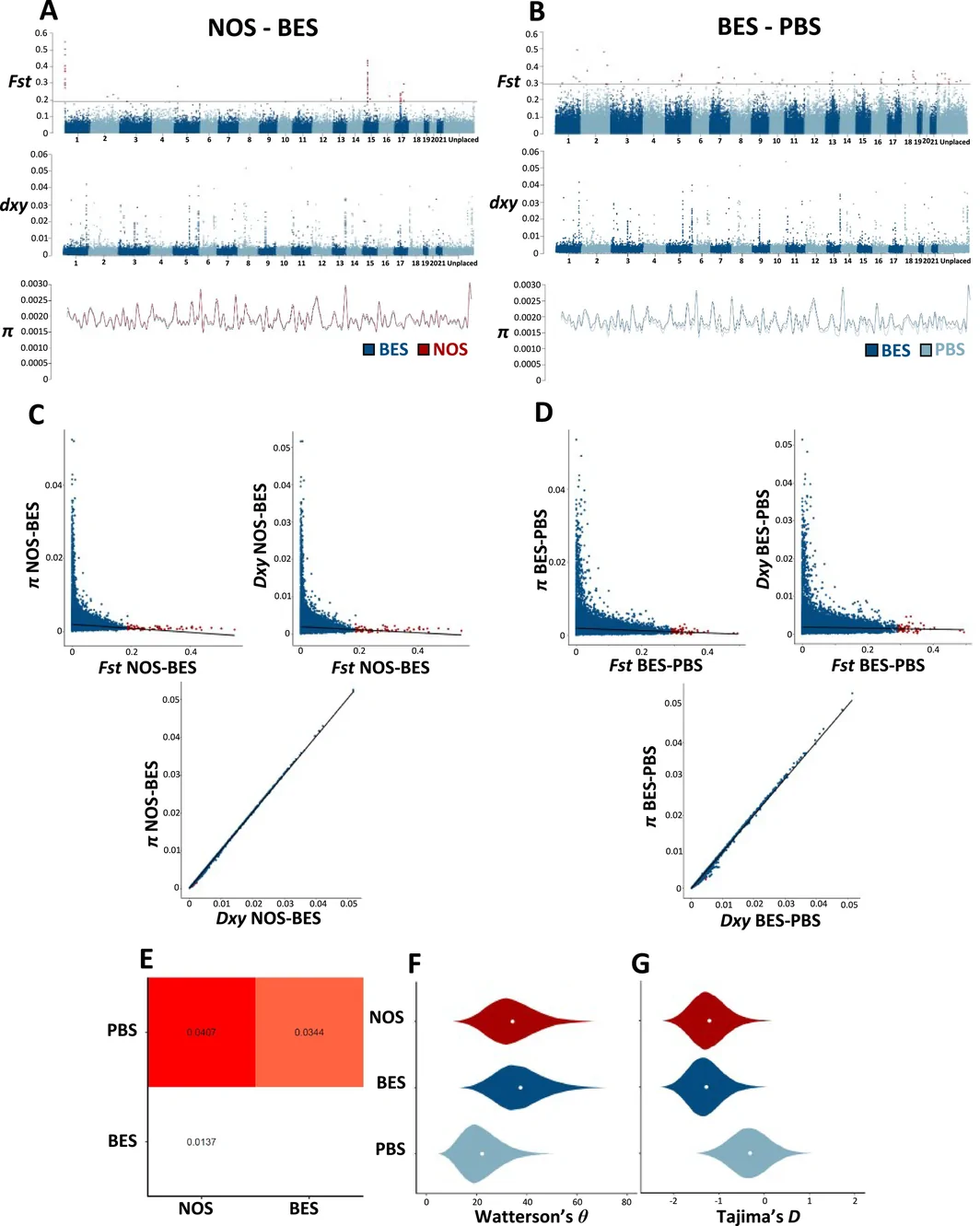
Population genomics statistics showing the role of selection and genetic drift on the origin and maintenance of Harbour porpoise populations in the Baltic region. (A) NOS‐BES Fst, dxy and π statistic values. The populations consist only of individuals with more than 75% ancestry to the given cluster under K3. Each dot represents a 20 Kb non‐overlapping window, red dots represent outlier windows (0.05% top Fst) and pseudochromosomes are coloured in an alternating pattern to aid visualization. (B) BES‐PBS Fst, dxy and π statistic values. (C) Relationship between within population diversity (π), relative (Fst) and absolute (dxy) nucleotide distance between NOS and BES. Each dot represents a 20 Kb window, red dots stand for the top 0.05% Fst windows and the black lines indicate a regression line fitted with a linear model. (D) Relationships between π, Fst and dxy values between BES and PBS. (E) Heatmap of mean Fst across populations. (F) Watterson's θ values. (G) Tajima's D estimates.

Within‐population diversity followed a consistent pattern, with NOS showing the highest nucleotide diversity (*π* = 0.00192), followed by BES (*π* = 0.00187) and PBS (*π* = 0.00177). π was also heterogeneously distributed along the genome, and it showed similar patterns across the three populations, but always with PBS showing the lowest estimates (Figure S10). Watterson's θ showed the same ranking, with PBS displaying the lowest values across the genome (Figure 3F), consistent with a smaller effective population size in the innermost Baltic population. Tajima's D estimates were centered around negative values across all populations (Figure 3G), consistent with demographic expansion or recent selective sweeps, though PBS showed a distribution closest to zero with a notable tail above zero, possibly reflecting stronger genetic drift relative to selection or a recent population contraction.

Pairwise correlations between Fst, dxy and π were significant across all three population comparisons (Figure 3C,D; Figure S9), with negative relationships for π‐Fst and dxy‐Fst, and a positive relationship for π‐dxy. The π‐Fst and dxy‐Fst slopes were steepest in the NOS‐BES comparison, where Fst outlier windows showed a 35% reduction in dxy and a 49% reduction in π relative to genome‐wide averages (Figure 3C), compared to 15% and 32% reductions respectively in BES‐PBS outliers (Figure 3D). Regarding the π‐dxy correlation, most genomic windows clustered near the 1:1 line, with Fst outliers (highlighted in red) deviating towards higher absolute divergence (dxy) than within population diversity (π). The NOS‐BES comparison showed less spread in π‐dxy values than BES‐PBS and NOS‐PBS, which both contained windows with jointly elevated π and dxy.

### 3.4 Selection Scans

To identify genomic regions potentially under local adaptation, we employed three complementary outlier SNP scans (Pcadapt, per‐site Fst, and LFMM using sea surface salinity as the environmental variable) alongside cross‐population extended haplotype homozygosity (XP‐EHH) analyses to detect selective sweeps. Pcadapt was the genome scan that identified the largest number of outlier SNPs, followed by LFMM and the top 1% Fst SNPs (Figure 4A). Considering only the 78 SNPs shared across all three methods as putative adaptive SNPs (Table S6), we found that BES and PBS individuals (both inhabiting low‐salinity environments) consistently shared the same genotype at these loci, while NOS individuals (from the high‐salinity Skagerrak strait and North Sea areas) presented a divergent genotype (Figure 4B). These 78 SNPs mapped within 20 Kb of 59 protein‐coding genes in the vaquita reference genome (Tables S7 and S8), of which 33 contained at least one intragenic SNP. Gene Ontology (GO) enrichment analyses revealed 33 significantly enriched GO terms (Fisher's exact test *p* < 0.01; Table S9) across the 59 protein‐coding candidate genes, with the most gene‐rich terms being cellular process (48 genes), membrane (35 genes), and protein‐containing complex (26 genes).

**FIGURE 4:**
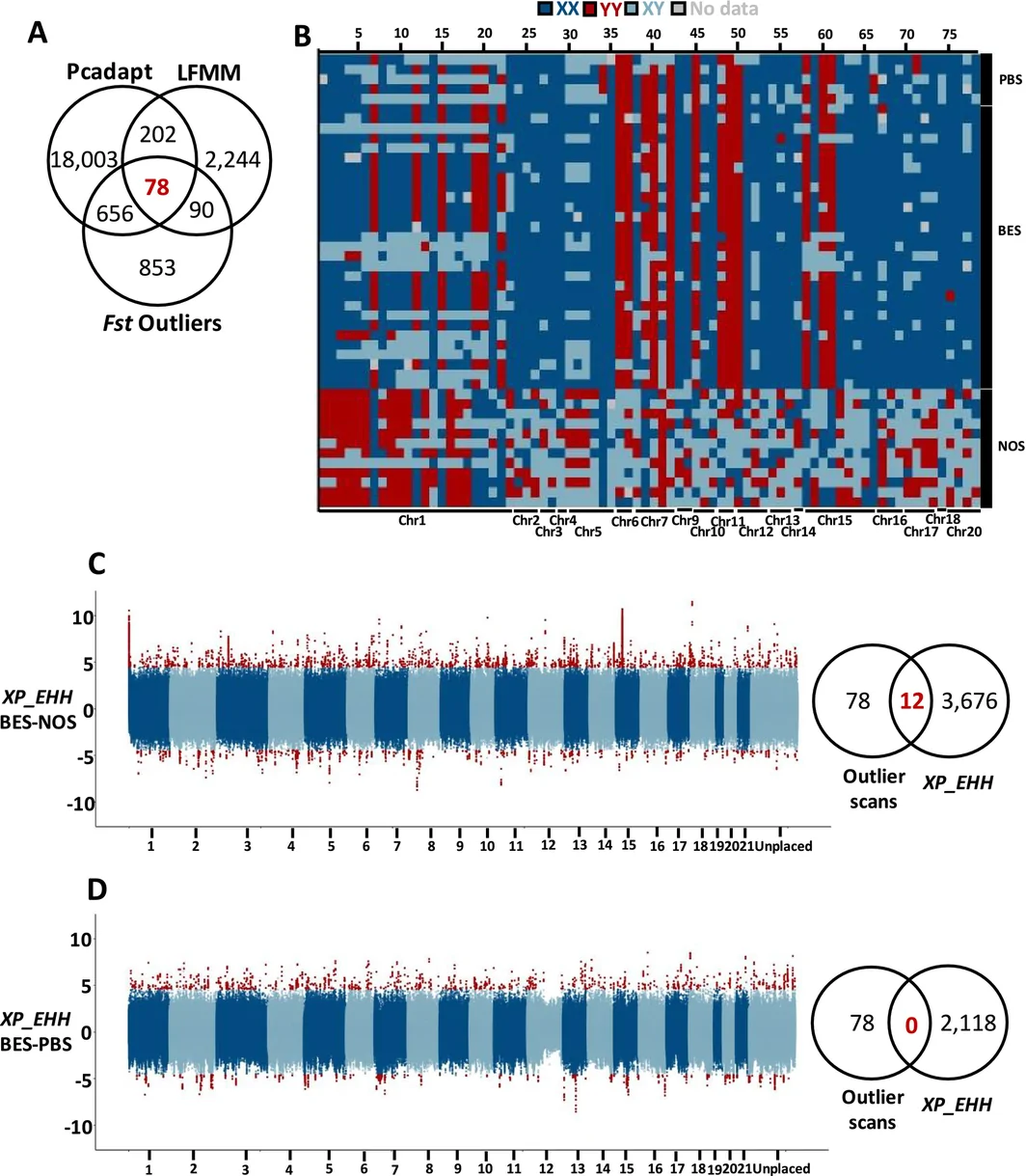
Local adaptation analysis on Harbour porpoises from the Baltic region. (A) Venn diagram of the number of putative adaptive SNPs identified by *Pcadapt*, LFMM and top 0.01% *Fst* SNPs. (B) Genotypes of the 78 SNPs found under putative selection. Each row represents one individual and the population to which it belongs is annotated at the right. Dark blue, red, light blue and grey show homozygous SNPs for the reference allele, homozygous SNPs for the alternative allele, heterozygous SNPs and missing data, respectively. Each column represents one SNP position and their pseudochromosome position is annotated at the bottom. (C) NOS‐BES XP‐EHH values. Positive scores suggest recent positive selection in BES, while negative one's positive selection in NOS. (D) BES‐PBS XP‐EHH scores, positive scores suggest recent positive selection in BES, while negative scores positive selection in PBS. Each dot represents a SNP, and putative selective sweeps are coloured in red.

XP‐EHH analyses revealed widespread signatures of selection across all three population comparisons, with the largest number of candidate regions identified in the NOS‐PBS comparison (873 regions; 2290 putative sweeps; Figure S11), followed by NOS‐BES (526 regions; 3676 sweeps; Figure 4C) and BES‐PBS (365 regions; 2117 sweeps; Figure 4C,D) (Figure S11). Notably, 12 of the 78 putative adaptive SNPs overlapped with selective sweep regions in the NOS‐BES scan (Figure 4C), 2 in the NOS‐PBS scan (Figure S11), while none were identified in the BES‐PBS scan (Figure 4D), suggesting that the strongest adaptive divergence occurred between the North Sea and Belt Sea populations.

Integrating windowed Fst estimates, outlier and selective sweep scans, we identified two genomic regions showing convergent evidence of local adaptation between NOS and BES: one on pseudochromosome 1 (Scaffold_2264, 0–280,000 bp; Figure 5A) and one on pseudochromosome 15 (Scaffold_9915, 12,250,000–12,800,000 bp; Figure 5B). Both regions displayed the highest levels of Fst (Figure 3A), the strongest XP‐EHH signals (Figure 4C), the largest numbers of putative adaptive SNPs and selective sweeps (225 and 364 sweeps for pChr1 and pChr15, respectively), and contained all 12 SNPs identified by both outlier and sweep scans. Further, allele frequencies at these SNPs were similar between BES and PBS, but highly divergent from NOS (Figure 5C), consistent with shared adaptation to low‐salinity conditions in the two Baltic populations.

**FIGURE 5:**
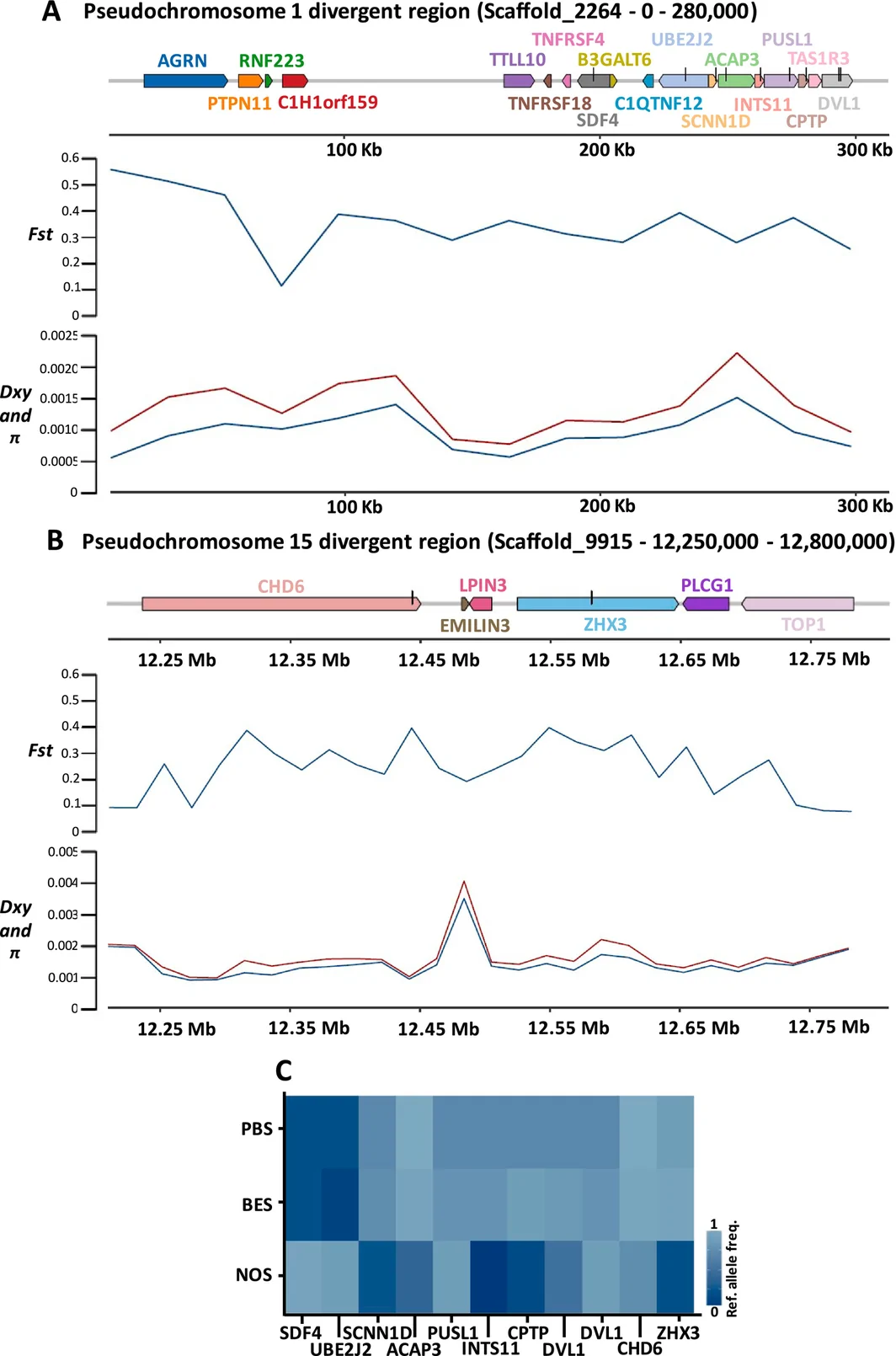
Genomic landscape of the two divergent regions between NOS and BES populations. (A) Pseudochromosome 1, Scaffold_2264. (B) Pseudochromosome 15, Scaffold_9915. The annotated genes, the putative adaptive SNPs (vertical lines), *Fst*, *dxy* (red) and *π* (dark blue) values are shown in different panels for both divergent regions. (C) Population allele frequencies of the 11 putative adaptive SNPs also identified as selective sweeps in pseudochromosome 1 divergent region. Each row represents the reference allele frequencies of one population, ranging from 0 (all alleles were different from the reference) to 1 (all alleles were the same as the reference). Each column represents one SNP position, the gene where the SNP is located is annotated at the bottom.

The peak on pseudochromosome 1 is located in a gene‐rich region containing at least 21 protein‐coding genes and 9 intragenic putative adaptive SNPs (Figure 5A, Table S10). To prioritize among these candidates, we ranked the underlying genes by the strength of their selection signal (Fst and XP‐EHH) and cross‐referenced the top‐ranking genes against their known functional annotation. SCNN1D emerged as the strongest candidate on both criteria: besides showing among the highest selection‐signal values in the region, it encodes part of the epithelial Na^+^ channels (ENaC), a heterotrimer that arose at the water‐to‐land transition of vertebrates and plays a central role in extracellular fluid homeostasis (Kashlan et al. 2024). Interestingly, the putative adaptive SNP within SCNN1D is a non‐synonymous mutation located in exon 8, with Fst values of 0.57 and 0.52 for the NOS‐BES and NOS‐PBS comparisons respectively, and an XP‐EHH value of 7.9 in the NOS‐BES scan, among the highest values observed genome‐wide.

The peak on pseudochromosome 15 contains 6 protein coding genes and 2 intragenic putative adaptive SNPs (Figure 5B, Table S11) in the CHD6 and ZHX3 genes. CHD6 encodes a chromodomain‐helicase‐DNA‐binding (CHD) protein involved in chromatin remodelling, and the regulation of transcription, replication, and DNA repair (Manning and Yusufzai 2017). ZHX3 encodes a zinc finger and homeobox transcription factor implicated in cell differentiation and organ development in the liver, kidneys, and blood cells (Liu et al. 2015).

### 3.5 Large‐Scale Genotyping

To validate population assignments at a larger geographic scale and trace the distribution of putative adaptive variation across Baltic waters, we developed a reduced SNP panel based on the 192 most differentiated SNPs (Fst range: 0.53–1.0; Table S10), including the putative adaptive SNP within SCNN1D. After Fluidigm genotyping and quality control (retaining SNPs with > 75% call rate, > 90% auto confidence, and error rates below 25% across replicate comparisons) 149 reliable SNPs were retained (Table S4) and genotyped across 376 unique individuals (Figure S12; Table S2). The panel recapitulated the three‐population structure identified by WGR: PCA and admixture analyses clearly separated NOS, BES and PBS individuals (Figure S13) and all 46 WGS individuals previously assigned to a population under K3 received identical assignments from the panel. Further, ~95% of replicates (i.e., samples genotyped twice with Fluidigm) were assigned to the same cluster, confirming the panel's accuracy and reproducibility across both WGS‐Fluidigm and Fluidigm‐Fluidigm comparisons.

Since harbor porpoises are much more common in the Belt Sea area than in the inner Baltic (Carlén et al. 2018), our dataset included considerably more samples from the BES region (232) than from the BES‐PBS transition zone (43) or the PBS region (39). Accordingly, most individuals were assigned to BES (284), followed by NOS (85) and PBS (7). Restricting analyses to the reproductive season (May–October), when the BES and PBS porpoises are geographically segregated (Carlén et al. 2018), did not substantially improve the match between sampling location and assigned population (Figure S14; Table S12). When further restricting the analysis to bycaught individuals during this period (Figure S15; Table S13), the number of migrants across population boundaries decreased, yet a significant number of BES individuals were still assigned in the NOS region and vice versa.

Crucially, the larger set of assigned individuals allowed us to characterize the distribution of the putative adaptive SNP within SCNN1D across populations. Allele frequencies at this SNP showed a striking divergence along the salinity gradient: individuals assigned to NOS carried the C allele at a frequency of 0.70, while those assigned to BES and PBS showed a C allele frequency of only 0.20 (Figure 6B,D). This near‐identical allele frequency in BES and PBS, contrasting sharply with NOS, mirrors the pattern observed genome‐wide at putative adaptive SNPs (Figure 4B) and is consistent with shared or parallel selection for osmoregulatory function across both low‐salinity Baltic populations. Importantly, the persistence of this allele frequency difference despite ongoing gene flow between populations argues for active positive selection maintaining divergence at this locus, rather than neutral differentiation by drift alone.

**FIGURE 6:**
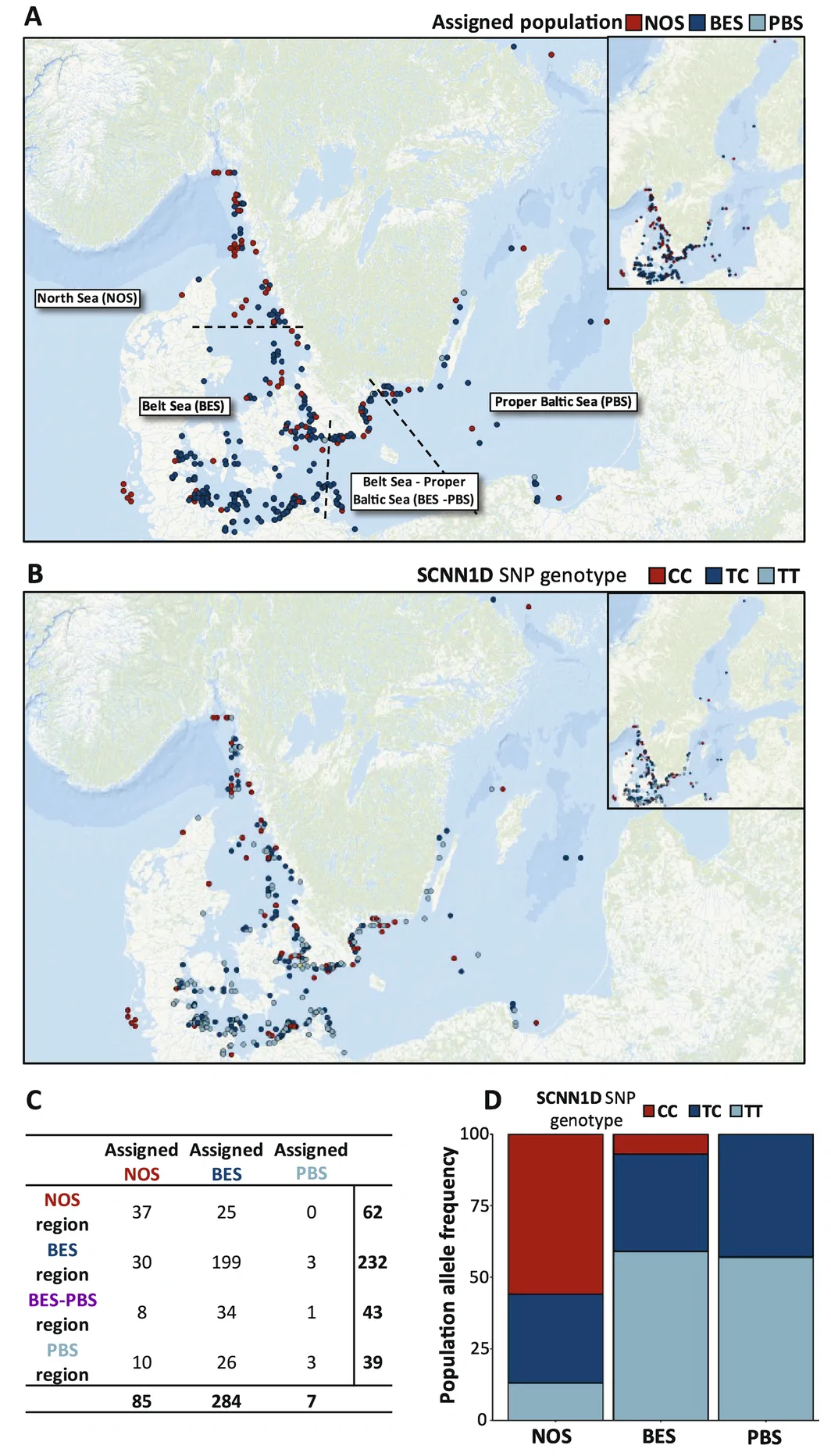
Population assignment and cline analysis of the putative adaptive SNP located in the osmoregulatory gene SCNN1D. (A) Assigned populations of the 376 Harbour porpoises. (B) Genotypes of the SCNN1D intragenic SNP of the 376 Harbour porpoises assigned to populations. (C) Assigned populations and sampling region for each of the 376 Harbour porpoises. (D) Population allele frequencies of the SCNN1D intragenic SNP.

## 4 Discussion

### 4.1 Postglacial Colonization of the Baltic

Our demographic modelling revealed that the diversification of Baltic harbour porpoises followed a sequential colonization pattern, with BES diverging first from an ancestral NOS population, followed by an independent divergence of PBS from NOS rather than from the geographically adjacent BES population (Figure 2). This topology was consistently supported across models of varying complexity, and the results proved robust to the mutation rate assumed: likelihoods for scenarios 6a and 6c did not differ significantly between mutation rates, and their relative support remained consistent across the three rates tested (Figure S16); divergence time estimates were similarly stable (CV ~1%–15%), whereas Ne estimates were more sensitive to this parameter (CV 8%–148%, driven largely by lack of convergence due to low number of replicates ran; Table S14), thus Ne estimates should be interpreted with caution. The inferred topology is further congruent with the population structure results, where admixed individuals were exclusively found between NOS and BES, suggesting these two populations maintain the highest connectivity. The independent origin of PBS from NOS, rather than from BES, is somewhat counterintuitive given the geographic arrangement of the three populations, but may reflect the existence of a historical dispersal corridor between the North Sea and the inner Baltic that bypassed the Belt Sea, potentially across the Öresund Strait, situated between the areas of Copenhagen/Denmark and Malmö/Sweden (Figure 1; Björck 1995).

Our models could not discriminate between two equally supported hypotheses regarding colonization timing: one placing the initial BES divergence within the Baltic Sea period (8044 yBP) and one extending colonization back to the Yoldia Sea period (11,805 yBP). Both periods correspond to times when the Baltic basin was connected to the North Sea, providing a marine corridor for colonization. Notably, in the best‐supported Yoldia Sea scenario (Figure 2B), the second split (the divergence of PBS from NOS) falls after Baltic Sea formation (~7854 yBP), suggesting that regardless of when BES first colonized the basin, PBS colonization likely occurred under the modern brackish conditions of the Baltic Sea. The inability to discriminate between the two timing hypotheses highlights the challenge of resolving ancient demographic events from contemporary genomic data alone. Interestingly, archaeological evidence of harbor porpoise exploitation in the eastern Baltic dating to the Neolithic (ca. 6000–4000 yBP) confirms that the species was already established in the basin by that time, consistent with our demographic estimates placing colonization before ~4000 yBP (Lõugas and Bērziņš 2023). Future analyses incorporating ancient DNA from these subfossil remains could help resolve the ambiguity between the Yoldia and Baltic Sea colonization scenarios. Nevertheless, both scenarios place the colonization of the Baltic within a postglacial timeframe of less than ~12,000 years, making the divergence of Baltic harbor porpoises (and the associated osmoregulatory adaptations) a striking example of rapid evolution in a recently established marine environment.

### 4.2 Harbour Porpoise Diversification in the Baltic: Demography Versus Selection

We found two distinct patterns of genome‐wide differentiation (Fst) in our population comparisons: genomic islands of differentiation in the NOS‐BES comparison (Figure 3A) and relatively uniform differentiation along the genome in the NOS‐PBS and BES‐PBS comparisons (Figure 3B; Figure S9). Despite the absence of pronounced Fst peaks, overall differentiation levels were paradoxically higher between PBS and both NOS (~0.04) and BES (~0.035) than between NOS and BES (~0.015). This is surprising in light of the demographic inference: since PBS diverged more recently from NOS than BES did, one would expect PBS to be genomically more related to NOS, not more differentiated. Several non‐mutually exclusive processes could explain this paradox. First, the low Ne of PBS—reflected in reduced Watterson's θ (Figure 3F) and nucleotide diversity (π) (Figure S10), and previously reported high inbreeding and low heterozygosity (Celemín et al. 2025)—would substantially accelerate genetic drift, causing rapid and stochastic allele frequency shifts across the genome that inflate genome‐wide Fst independently of divergence time (Kardos et al. 2021). Second, a strong founder effect during colonization of the inner Baltic may have captured only a fraction of ancestral NOS variation, producing an immediate divergence regardless of colonization timing. Third, PBS has experienced a well‐documented severe and recent demographic decline (Carlén et al. 2021; Amundin et al. 2022), which would have further eroded genetic diversity and amplified drift over a very short timescale. Finally, reduced gene flow between PBS and the other populations, as supported by our demographic models, would have allowed drift to accumulate more freely in PBS without the homogenizing effect of migration. Together, these processes likely explain both the uniformly elevated genome‐wide Fst between PBS and the other populations and the extreme clustering of PBS individuals in the PCA (Figure 1B) and are consistent with similar patterns of low Ne and drift‐dominated differentiation reported for the Black Sea harbour porpoise subspecies (Celemín et al. 2025). We acknowledge the small and unequal number of genomes available per population (NOS = 12, BES = 30, PBS = 5), which may affect allele frequency estimates, though large SNP datasets have been shown to mitigate the impact of small sample sizes on population genomic statistics (Nazareno et al. 2017).

Across most of the genome, within‐population diversity (π) is nearly as large as absolute divergence (dxy) in all three pairwise comparisons, with the majority of genomic windows clustering near the 1:1 line (Figure 3; Figure S9)—the expectation for populations sharing very recent common ancestry (Irwin et al. 2018), consistent with our demographic estimates placing all divergence events within the last ~12,000 years. Against this background of shallow, largely uniform divergence, the two genomic islands of elevated relative differentiation (Fst) exclusive to the NOS‐BES comparison (Figure 3A) stand out as strong candidates for divergent selection. These high‐Fst regions are associated with locally reduced absolute divergence (dxy) and diversity (π) (Figure 3C), a signature of linked selection and local adaptation through selective sweeps (Irwin et al. 2018), confirmed by the strong XP‐EHH signals retrieved in both regions (Figure 4C). The large positive XP‐EHH values suggest recent positive selection in the BES population and purifying selection in NOS (Sabeti et al. 2007; Gautier and Vitalis 2012). Notably, no equivalent selective sweep signatures or Fst peaks were detected in the comparisons involving PBS. This is consistent with the reduced efficacy of selection relative to drift expected in populations with very low Ne, where even strongly advantageous mutations may fail to produce detectable sweep signatures (Charlesworth 2009). The absence of selection signatures in PBS comparisons therefore likely reflects not an absence of selective pressure, but an inability to detect it against the background of pervasive drift. The fact that the strongest adaptive signatures are instead found in the NOS‐BES comparison (between populations with higher Ne, greater diversity, and substantial ongoing gene flow) underscores that divergent selection across the Baltic salinity gradient is most detectable precisely where demographic conditions allow selection to overcome drift.

### 4.3 Genetic Basis of Osmoregulation to Baltic Salinity Gradient

We identified two highly divergent genomic regions between NOS and BES populations containing candidate genes associated with osmoregulation (Figure 5). The most compelling candidate is SCNN1D, which encodes the delta subunit of the amiloride‐sensitive epithelial Na + channel (ENaC), a highly specialized sodium‐permeable, non‐voltage‐sensitive ion channel essential for salt and water homeostasis in tetrapod vertebrates (Wichmann and Althaus 2020). ENaC is an obligate heterotrimer composed of four paralogous subunits—α (SCNN1A), β (SCNN1B), γ (SCNN1G), and δ (SCNN1D)—located in the apical membrane of cells lining the aldosterone‐sensitive distal nephron, where they facilitate Na^+^ reabsorption critical for body osmoregulation (Rossier et al. 2015). ENaC regulates its own activity through a process termed Na^+^ self‐inhibition, whereby extracellular Na^+^ binds to regulatory sites on the channel that limit further sodium entry into the cell (Wang et al. 2024). The δ subunit emerged from an α duplication in a jawed‐fish ancestor (Wang et al. 2022) and, unlike the α, β, and γ subunits, which are expressed mainly in the kidney, lungs, and colon (Enuka et al. 2012), is expressed predominantly in the brain (Giraldez et al. 2012), where it interferes in cardiovascular regulation through salt‐induced depolarization of vasopressin neurons (Sharma et al. 2017; Wichmann and Althaus 2020). ENaC α and δ subunits are interchangeable in assembled channels and confer distinct functional properties: while αβγ ENaC shows robust Na^+^ self‐inhibition and protease activation, δβγ ENaC is less sensitive to both (Ji et al. 2006; Haerteis et al. 2009), suggesting that the identity of the δ subunit shapes the channel's sensitivity to extracellular Na^+^, with potential consequences for osmoregulation in variable salinity environments (Ahmad et al. 2023).

The ENaC δ subunit gene SCNN1D is located within the most divergent genomic region between NOS and BES (Figure 3A), and the selective sweep signature at this locus indicates recent positive selection in BES and purifying selection in NOS (Figure 4C). The non‐synonymous mutation in exon 8 (with Fst values of 0.57 and 0.52 for NOS‐BES and NOS‐PBS respectively) represents one of the strongest candidates for adaptive divergence identified in this study. Interestingly, other candidate genes in the pseudochromosome 1 peak (Table S10) are significantly enriched in GO terms associated with neurogenesis and nervous system development (GO:0022008, GO:0061564, GO:0048699, GO:0007399, GO:0150034, GO:0048666; Table S8), including AGRN, PTPN11, SDF4, ACAP3 and DVL1, several of which also carry putative adaptive SNPs identified as selective sweeps (Figure 4C). Given the predominant expression of the ENaC δ subunit in the brain and its role in salt‐induced cardiovascular regulation, the co‐localization of neurogenesis‐related candidate genes in the same divergent region suggests a coordinated reorganization of neurological osmoregulatory pathways alongside renal adaptation.

Since NOS and BES porpoises regularly move between high and low salinity areas (Carlén et al. 2018; Sveegaard et al. 2015; Celemín et al. 2025), they must cope with the osmotic and cardiovascular challenges imposed by fluctuating Na^+^ environments (Sundell et al. 2021). In high‐salinity conditions, ENaC's Na^+^ self‐inhibition mechanism could limit further sodium entry into cells, maintaining stable osmotic pressure (Mutchler et al. 2021; Wang et al. 2024), while in low‐salinity environments, increased ENaC activity could facilitate Na^+^ reabsorption to prevent ionic depletion. The brain‐mediated mechanism may operate in parallel: high dietary salt intake increases ENaC expression and activity in vasopressin neurons, supporting cardiovascular homeostasis during osmotic challenge (Sharma et al. 2017). The SCNN1D gene, with its dual roles in renal Na + reabsorption and neurological blood pressure regulation, is therefore well positioned to underlie the physiological flexibility required by porpoises navigating the steep Baltic salinity gradient. These findings extend current knowledge on the molecular basis of cetacean adaptation to low‐salinity environments (Zhou et al. 2018; Ramos et al. 2023), and identify a candidate gene under recent positive selection within a divergence timeframe of less than ~12,000 years—placing Baltic harbour porpoises among the most compelling examples of rapid osmoregulatory adaptation documented in marine mammals. Functional analyses will nonetheless be essential to confirm the physiological consequences of the exon 8 substitution and determine whether it alters Na^+^ self‐inhibition kinetics, protease sensitivity, or tissue‐specific expression in porpoises inhabiting contrasting salinity environments.

### 4.4 SNP Panel as a Tool to Study Rapid Adaptation and Population Connectivity in Baltic Harbour Porpoises

The development of reduced SNP panels based on highly differentiated loci has proven particularly valuable for studying population structure and adaptive variation in marine species with overlapping distributions (Östergren et al. 2021; Therkildsen et al. 2013; Bonanomi et al. 2015, 2016). Our final panel of 149 SNPs correctly reassigned 100% of WGR individuals to their population of origin and reliably assigned 376 opportunistically sampled individuals to one of the three Baltic populations, demonstrating sufficient power for large‐scale population assignment from bycaught or stranded specimens. Applying this panel at scale confirmed the widespread geographic overlap between populations throughout the Baltic region, even during the reproductive season when populations are expected to be most segregated (Table S12; Figure S14). The persistence of a significant number of migrants across population boundaries among bycaught individuals during May–October (Table S13; Figure S15) indicates that population overlap reflects genuine year‐round movement, resulting in the inferred ongoing gene flow between populations experiencing markedly different salinity environments. This has important implications for understanding how adaptive variation is maintained: the large allele frequency differences observed at putative adaptive loci are sustained despite this connectivity, providing strong evidence that divergent selection actively maintains differentiation against the homogenizing effect of gene flow.

Assigning individuals to populations allowed us to robustly characterize the geographic distribution of the putative adaptive non‐synonymous SNP within SCNN1D at a scale that rules out sampling artefacts from the small and unequal WGR sample sizes. The striking allele frequency difference between NOS (C allele frequency = 0.70) and both BES and PBS (C allele frequency = 0.20) confirms that this locus is among the most strongly differentiated in the genome and that its divergence is geographically consistent with the salinity gradient. The convergent allele frequencies in BES and PBS are particularly noteworthy considering our demographic finding that PBS colonized the inner Baltic independently from NOS rather than from BES: the similarity at this adaptive locus therefore likely reflects parallel selective pressures imposed by the shared low‐salinity environment rather than shared ancestry. This pattern, mirrored genome‐wide across all 78 putative adaptive SNPs (Figure 4B), points to salinity as a pervasive and repeatable selective force shaping genomic divergence across the Baltic gradient. Together, these results suggest that divergence at SCNN1D (and at the broader set of putative adaptive loci) reflects a salinity‐driven selective barrier actively maintained against gene flow, making Baltic harbour porpoises a valuable model for studying the genomic basis of rapid adaptation to environmental gradients in marine mammals. Beyond their value for evolutionary inference, the genomic resources developed here offer a practical tool for cost‐effective population monitoring, enabling assignment of individual bycaught or stranded porpoises to their population of origin—an application of particular importance for tracking demographic trends in the critically endangered PBS population (Carlström et al. 2023).

## Author Contributions

E.C. and R.T. designed the study. E.C. and K.H. performed laboratory work. R.T. provided funding. H.B., P.B., M.D., J.D., C.L., I.P., A.R., U.S. and L.W. provided samples and associated biological information. E.C. executed bioinformatic analyses and analysed the data. K.E.T. performed the fastsimcoal simulations. E.C. and R.T. interpreted the results. E.C. wrote the draft manuscript with input from R.T. and K.E.T. All authors edited and approved the final manuscript.

## Funding

Harbour porpoises from Mecklenburg‐Prepomerania were collected under contracts funded by the Landesamt für Umwelt, Naturschutz und Geologie (Contract Reg. Nr. 20.42117). Harbour porpoises from Schleswig‐Holstein Germany were collected through the stranding network and monitoring program funded by the Schleswig‐Holstein Ministry of Energy Transition, Climate Protection Environment and Nature (MEKUN) and the National Park Office of Schleswig‐Holstein. This work was financially supported by the University of Potsdam and the Bundesamt für Naturschutz (FKZ 3521532214 and FKZ 352520020I) granted to R.T.

## Disclosure

Benefit‐Sharing Statement: A research collaboration was developed with scientists of Baltic Sea countries providing genetic samples; all collaborators are included as co‐authors, the results of research have been shared with the provider communities and the broader scientific community (see above), and the research addresses a priority concern, in this case the conservation of the critically endangered Proper Baltic Sea Harbour porpoise population. More broadly, our group is committed to international scientific partnerships, as well as institutional capacity building.

## Ethics Statement

No live animal was targeted in the course of this project. Sample collection was carried out from carcasses by authorized institutions under the respective national legislation. All the research complies with applicable laws on sampling from natural populations and animal experimentation (including the ARRIVE guidelines).

## Conflicts of Interest

The authors declare no conflicts of interest.

## Supporting information


**Table S1:** WGS sample information.
**Table S2:** Reduced SNP panel sample information.
**Table S3:** Testing scheme in fastsimcoal2.
**Table S4:** 192 selected SNPs for the reduced panel of SNPs.
**Table S5:** Estimated metrics per population with fastsimcoal2.
**Table S6:** 78 putative adaptive SNPs Candidate genes.
**Table S7:** Curated BLAST hits of the 78 putative adaptive SNPs.
**Table S8:** Candidate genes.
**Table S9:** Significantly enriched GO terms candidate genes.
**Table S10:** Genes and adaptive SNPs Pseudochromosome 1.
**Table S11:** Genes and adaptive SNPs Pseudochromosome 15.
**Table S12:** May–October porpoises assigned populations.
**Table S13:** Bycaught May–October porpoises assigned populations.
**Table S14:** Mutation rate sensitivity analysis.
**Figure S1:** Postglacial geological history of the Baltic Sea basin.
**Figure S2:** Harbour porpoise—vaquita genome alignment.
**Figure S3:** Bioinformatic pipeline.
**Figure S4:** Demographic scenarios tested with fastsimcoal2.
**Figure S5:** Graphical visualization of fastsimcoal2 testing scheme.
**Figure S6:** Admixture K2 and Delta K method results.
**Figure S7:** Assignment and location WGS samples.
**Figure S8:** Likelihood distributions fastsimcoal2 tested scenarios.
**Figure S9:** NOS‐PBS Fst, dxy and π statistic values and correlations.
**Figure S10:** Within population diversity (π) values calculated with pixy.
**Figure S11:** NOS‐PBS XP_EHH values.
**Figure S12:** Map of porpoises genotyped with the SNP panel.
**Figure S13:** Evaluation of power of assignment of the SNP panel.
**Figure S14:** Map of May–October porpoises assigned populations.
**Figure S15:** Map of bycaught May–October porpoises assigned pop.
**Figure S16:** Likelihood distributions of altered mutation rates.

## Data Availability

Genomic sequencing data were previously deposited under BioProject PRJNA997110. Raw reads for the 8 additional individuals can be found at the NCBI database under the BioProject PRJNA1543563. Bioinformatic scripts are available in a public GitHub repository: https://github.com/kikecelemin/Baltic_Pho_pho.git.
