# Supplementary material for: Drift and Selection in a Recently Colonized Sea: The Genomic Basis of Adaptation to Low‐Salinity Waters in Baltic Harbour Porpoises
Source: Mol Ecol. 2026 Oct 16;35(20):e70595. doi: 10.1111/mec.70595 (PMC13675720; doi:10.1111/mec.70595)
Supplement: Supplementary file 1 [file MEC-35-e70595-s001.docx]

**Supplemental Information for:**

**Drift and selection in a recently colonized sea: the genomic basis of adaptation to low-salinity waters in Baltic Harbour porpoises**

Enrique Celemín, Kristín E. Tiedemann, Katja Havenstein, Harald Benke, Per Berggren, Michael Dähne, Judith Denkinger, Lisa Klemens, Christina Lockyer, Iwona Pawliczka, Anna Roos, Ursula Siebert, Linda Westphal, Ralph Tiedemann

**Table of Contents:**

| **Table S1: WGS sample information** | **Page 2-3** |
| --- | --- |
| **Table S2: Reduced SNP panel sample information** | **Page 4-14** |
| **Table S3: Testing scheme in fastsimcoal2** | **Page 15** |
| **Table S4: 192 selected SNPs for the reduced panel of SNPs** | **Page 16-22** |
| **Table S5: Estimated metrics per population with fastsimcoal2** | **Page 23** |
| **Table S6: 78 putative adaptive SNPs Candidate genes** | **Page 24-25** |
| **Table S7: Curated BLAST hits of the 78 putative adaptive SNPs** | **Page 26-27** |
| **Table S8: Candidate genes** | **Page 28-29** |
| **Table S9: Significantly enriched GO terms candidate genes** | **Page 29-31** |
| **Table S10: Genes and adaptive SNPs Pseudochromosome 1** | **Page 32** |
| **Table S11: Genes and adaptive SNPs Pseudochromosome 15** | **Page 32-33** |
| **Table S12: May-October porpoises assigned populations** | **Page 33** |
| **Table S13: Bycaught May-October porpoises assigned populations** | **Page 33** |
| **Table S14: Mutation rate sensitivity analysis** | **Page 34** |
| **Fig S1: Postglacial geological history of the Baltic Sea basin** | **Page 35** |
| **Fig S2: Harbour porpoise - vaquita genome alignment** | **Page 36** |
| **Fig S3: Bioinformatic pipeline** | **Page 37** |
| **Fig S4: Demographic scenarios tested with fastsimcoal2** | **Page 38** |
| **Fig S5: Graphical visualization of fastsimcoal2 testing scheme** | **Page 39** |
| **Fig S6: Admixture K2 and Delta K method results** | **Page 40** |
| **Fig S7: Assignment and location WGS samples** | **Page 40** |
| **Fig S8: Likelihood distributions fastsimcoal2 tested scenarios** | **Page 41** |
| **Fig S9: NOS-PBS Fst, dxy and π statistic values and correlations** | **Page 42** |
| **Fig S10: Within population diversity (π) values calculated with pixy** | **Page 43** |
| **Fig S11: NOS-PBS XP_EHH values** | **Page 43** |
| **Fig S12: Map of porpoises genotyped with the SNP panel** | **Page 44** |
| **Fig S13: Evaluation of power of assignment of the SNP panel** | **Page 45** |
| **Fig S14: Map of May-October porpoises assigned populations** | **Page 46** |
| **Fig S15: Map of bycaught May-October porpoises assigned pop.** | **Page 47** |
| **Fig S16: Likelihood distributions of altered mutation rates** | **Page 48** |

**Table S1:** Sample information of the individuals used in the WGS project indicating sample ID, region, country where the porpoise was found, coordinates, sex, discovery date, bycaught/stranded and final coverage after filtering. ^*^ indicates the samples that were removed from the analysis due to low coverage or for being a first relative of another sample. ^‡^ indicates the samples that were used in the demographic history analysis.

| **Sample** | **Region** | **Country** | **Coordinates** | **Sex** | **Date** | **Final Coverage** |
| --- | --- | --- | --- | --- | --- | --- |
| **20773** | NOS | Germany | N54.5 W8.3 | F | 06/09/1989 | 23.25 |
| **4993** | NOS | Germany | N54.55 W8.4 | F | 10/05/2015 | 21 |
| **21069** | NOS | Germany | N54.6 W8.2 | F | 15/10/2003 | 18.51 |
| **4996^‡^** | NOS | Germany | N54.7 W8.2 | F | 10/07/2009 | 9 |
| **C2007-08047^‡^** | SKA | Sweden | N58.5 W11.1 | M | 23/09/2007 | 8.97 |
| **A2019-05574^‡^** | SKA | Sweden | N57.55 W11.93 | F | 11/06/2019 | 8.65 |
| **96-012^‡^** | SKA | Denmark | N57.37 W9.68 | F | 01/08/2013 | 7.99 |
| **C2012-00016^‡^** | SKA | Sweden | N59 W10.85 | M | 14/05/2011 | 7.4 |
| **A2005-05129** | SKA | Sweden | N58.5 W11.15 | F | 08/07/2004 | 5.78 |
| **A2016-05647** | KAT | Sweden | N57 W12 | F | 19/08/2016 | 6.52 |
| **5092** | BES | Germany | N54.51 W10.2 | F | 04/08/2009 | 37.01 |
| **5067** | BES | Germany | N54.54 W10.2 | F | 11/07/2009 | 23.95 |
| **5077** | BES | Germany | N54.55 W11.01 | F | 12/08/2009 | 19.7 |
| **B22-15** | BES | Germany | N54.485 W12 | M | 25/06/2015 | 12.8 |
| **B50-14** | BES | Germany | N54.5 W12 | F | 15/07/2014 | 10.85 |
| **B23-15** | BES | Germany | N54.26 W12.23 | F | 01/07/2015 | 9.8 |
| **1927^‡^** | BES | Denmark | N55.04 W10.78 | F | 12/08/2002 | 9.11 |
| **21206** | BES | Germany | N54.49 W10.2 | F | 13/08/2015 | 9.06 |
| **5058** | BES | Germany | N54.48 W10.2 | F | 24/07/2009 | 8.67 |
| **A1984-05062*** | BES | Sweden | N55.37 W13.15 | M | 05/07/1984 | 8.55 |
| **B43-15^‡^** | BES | Germany | N54.5 W12.66 | M | 08/10/2015 | 8.51 |
| **B58-15^‡^** | BES | Germany | N54.7 W13.36 | F | 28/09/2015 | 8.27 |
| **C2009-04450** | BES | Sweden | N55.35 W12.9 | F | 25/08/2009 | 7.91 |
| **1752** | BES | Germany | N54.49 W13 | F | 01/09/2001 | 7.8 |
| **B75-14^‡^** | BES | Germany | N54.7 W13.13 | F | 19/09/2014 | 7.71 |
| **A1988-05142^‡^** | BES | Sweden | N55.35 W13.25 | M | 21/07/1988 | 7.66 |
| **5023** | BES | Germany | N54.65 W10.3 | F | 30/07/2009 | 7.39 |
| **B41-14*** | BES | Germany | N54.31 W12.26 | F | 02/07/2014 | 7.35 |
| **98-028** | BES | Denmark | N55.32 W11.14 | F | 22/08/1998 | 7.33 |
| **20565** | BES | Germany | N55 W11 | M | 11/06/2014 | 7.22 |
| **A2022-00301** | BES | Sweden | N55.97 W12.75 | F | 02/05/2022 | 7.02 |
| **B34-13** | BES | Germany | N54.6 W11.37 | F | 03/09/2013 | 6.83 |
| **97-034^‡^** | BES | Denmark | N55 W10.23 | F | 01/08/1997 | 6.66 |
| **5071** | BES | Germany | N54.4 W11.1 | F | 04/08/2009 | 6.06 |
| **5013** | BES | Germany | N54.5 W11 | F | 14/07/2009 | 5.77 |
| **A2016-05698*** | BES | Sweden | N55.5 W13 | F | 15/09/2016 | 2.73 |
| **A2020-05733** | BES-PBS | Sweden | N55.4 W13.81 | M | 12/09/2020 | 9.47 |
| **A1986-05214** | BES-PBS | Sweden | N55.4 W13.84 | M | 08/11/1986 | 8.81 |
| **C1990-03105** | BES-PBS | Sweden | N55.9 W14.4 | M | 13/03/1991 | 8.65 |
| **A1995-05049** | BES-PBS | Sweden | N56 W14.5 | - | 27/05/1995 | 6.67 |
| **A2020-05731** | BES-PBS | Sweden | N55.4 W14 | F | 29/08/2020 | 6.06 |
| **C1989-05720** | PBS | Sweden | N56.1 W16.9 | F | 15/08/2009 | 25.02 |
| **C2004-5008** | PBS | Latvia | N57 W20.4 | F | 31/01/2004 | 23.78 |
| **C2004-5009** | PBS | Latvia | N57 W20 | M | 28/09/2014 | 21.32 |
| **Pol-58** | PBS | Poland | N54.5 W18.65 | F | 01/01/1998 | 14.94 |
| **Pol-68** | PBS | Poland | N55.3 W17.2 | F | 01/11/2000 | 14.06 |
| **A1993-05153** | PBS | Sweden | N56.1 W14.79 | M | 22/06/1993 | 9.67 |
| **A1993-05175^‡^** | PBS | Sweden | N56.5 W16.25 | M | 05/08/1993 | 8.91 |
| **Pol-72^‡^** | PBS | Poland | N54.8 W18.58 | F | 01/06/2001 | 8.88 |
| **A1984-05087^‡^** | PBS | Sweden | N57.4 W16.8 | M | 29/07/1984 | 8.4 |
| **A1997-05187** | PBS | Sweden | N56.1 W14.86 | F | 05/05/1996 | 8.2 |
| **A1986-05229** | PBS | Sweden | N56 W14.83 | M | 12/11/1986 | 8.2 |
| **C1991-04044** | PBS | Sweden | N56 W15 | M | 01/10/1991 | 8.02 |
| **Pol_53** | PBS | Poland | N54.7 W18.6 | M | 01/07/1996 | 6.68 |
| **G1989-07863** | PBS | Sweden | N56.1 W14.9 | M | 13/04/1989 | 6.6 |
| **C1988-05707** | PBS | Sweden | N56.08 W14.96 | F | 30/05/1988 | 6.42 |
| **A1987-05226** | PBS | Sweden | N56.06 W15.7 | M | 13/08/1987 | 6.38 |
| **A1997-05190** | PBS | Sweden | N58 W18 | F | 22/01/1997 | 6.14 |
| **Pol-74** | PBS | Poland | N54.7 W18.62 | F | 01/02/2002 | 5.54 |

**Table S2**: Sample information of the individuals genotyped on the reduced SNP panel indicating sample ID, region, country where the porpoise was found, coordinates, sex, discovery date and information whether the sample was included in the WGS project, replicated in the Fluidigm genotyping and if it was assigned to a population.

| **Sample** | **Region** | **Country** | **Coords** | **Sex** | **Date** | **WGS** | **Fluidigm replicate** | **Assigned** |
| --- | --- | --- | --- | --- | --- | --- | --- | --- |
| **706** | PBS | Finland | N62.00 W21.00 | M | 30/06/1996 | - | - | Y |
| **750** | PBS | Finland | N60.50 W19.00 | M | 01/06/1999 | - | - | Y |
| **1472** | BES | Germany | N54.45 W12.53 | F | 01/08/1999 | - | - | Y |
| **1695** | BES | Germany | N54.32 W13.08 | F | 01/07/2000 | - | - | Y |
| **1700** | KAT | Denmark | N56.18 W10.23 | M | 17/04/2000 | - | - | Y |
| **1747** | BES | Germany | N54.57 W10.02 | F | 01/08/2001 | - | - | Y |
| **1752** | BES | Germany | N54.49 W13.00 | F | 01/09/2001 | Y | - | Y |
| **1927** | BES | Denmark | N55.04 W10.78 | F | 12/08/2002 | Y | - | Y |
| **3098** | BES | Germany | N54.48 W11.00 | F | 01/08/2005 | - | - | Y |
| **3567** | BES | Denmark | N55.04 W10.47 | M | 11/10/2006 | - | - | Y |
| **4907** | BES | Germany | N54.50 W10.10 | F | 30/06/2009 | - | - | Y |
| **4993** | NOS | Germany | N54.55 W8.40 | F | 11/07/2009 | Y | - | Y |
| **4996** | NOS | Germany | N54.70 W8.20 | F | 10/07/2009 | Y | - | Y |
| **5013** | BES | Germany | N54.55 W11.10 | F | 14/07/2009 | Y | - | Y |
| **5023** | BES | Germany | N54.65 W10.30 | F | 30/07/2009 | Y | Y | Y |
| **5025** | BES | Germany | N54.55 W10.20 | F | 15/07/2009 | - | - | Y |
| **5027** | BES | Germany | N55.10 W9.60 | F | 20/07/2009 | - | - | Y |
| **5058** | BES | Germany | N54.48 W10.20 | F | 24/07/2009 | Y | - | Y |
| **5061** | BES | Germany | N55.05 W9.60 | F | 29/07/2009 | - | - | Y |
| **5067** | BES | Germany | N54.54 W10.25 | F | 12/08/2009 | Y | - | Y |
| **5069** | BES | Germany | N54.55 W10.00 | F | 11/08/2009 | - | Y | Y |
| **5071** | BES | Germany | N54.40 W11.10 | F | 04/08/2009 | Y | Y | Y |
| **5077** | BES | Germany | N54.55 W11.01 | F | 04/08/2009 | Y | - | Y |
| **5085** | BES | Germany | N54.16 W11.32 | F | 01/08/2009 | - | - | Y |
| **5086** | BES | Germany | N54.50 W9.90 | F | 14/08/2009 | - | - | Y |
| **5088** | BES | Germany | N54.60 W10.05 | F | 10/08/2009 | - | Y | Y |
| **5091** | BES | Germany | N55.10 W11.00 | F | 17/08/2009 | - | - | Y |
| **5092** | BES | Germany | N54.51 W10.20 | F | 15/08/2009 | Y | - | Y |
| **5160** | BES | Germany | N55.00 W11.00 | F | 13/08/2009 | - | - | Y |
| **5216** | BES | Germany | N55.15 W9.65 | F | 01/09/2009 | - | - | Y |
| **6480** | BES | Germany | N54.57 W10.10 | F | 28/07/2011 | - | - | Y |
| **6655** | BES | Germany | N55.16 W9.55 | F | 03/05/2012 | - | - | Y |
| **6662** | BES | Germany | N54.55 W11.15 | F | 03/06/2012 | - | - | Y |
| **6669** | BES | Germany | N54.57 W11.16 | F | 24/05/2012 | - | Y | Y |
| **6750** | BES | Germany | N54.45 W10.00 | F | 29/07/2012 | - | - | Y |
| **6751** | BES | Germany | N54.49 W10.00 | F | 25/07/2012 | - | - | Y |
| **6752** | BES | Germany | N54.00 W11.00 | F | 05/05/2012 | - | - | Y |
| **6754** | BES | Germany | N54.50 W10.15 | F | 22/06/2012 | - | Y | Y |
| **6755** | BES | Germany | N54.50 W11.00 | F | 31/07/2012 | - | - | Y |
| **6759** | BES | Germany | N54.50 W10.08 | F | 28/07/2012 | - | - | Y |
| **6801** | NOS | Germany | N54.45 W8.40 | F | 16/07/2012 | - | - | Y |
| **6848** | BES | Germany | N55.04 W9.51 | F | 28/07/2012 | - | Y | Y |
| **20042** | BES | Germany | N54.47 W9.85 | M | 19/01/2013 | - | - | Y |
| **20057** | BES | Germany | N54.33 W11.08 | F | 15/12/2012 | - | - | Y |
| **20153** | BES | Germany | N54.77 W9.96 | M | 21/02/2013 | - | - | Y |
| **20155** | BES | Germany | N54.01 W10.77 | M | 03/05/2013 | - | - | Y |
| **20565** | BES | Germany | N55.00 W11.10 | - | 11/06/2014 | Y | Y | Y |
| **20751** | BES | Germany | N55.10 W10.00 | F | 16/09/2014 | - | Y | Y |
| **20755** | BES | Germany | N55.00 W10.00 | F | 18/09/2014 | - | Y | Y |
| **20757** | BES | Germany | N55.06 W9.70 | F | 18/09/2014 | - | - | Y |
| **20773** | NOS | Germany | N54.50 W8.30 | F | 28/09/2014 | Y | - | Y |
| **21069** | NOS | Germany | N54.60 W8.20 | F | 10/05/2015 | Y | - | Y |
| **21088** | BES | Germany | N54.70 W10.08 | F | 26/05/2015 | - | - | - |
| **21091** | BES | Germany | N54.50 W10.02 | F | 17/06/2015 | - | - | Y |
| **21198** | BES | Germany | N54.55 W10.95 | F | 20/08/2015 | - | - | Y |
| **21203** | BES | Germany | N54.50 W10.00 | F | 17/08/2015 | - | - | - |
| **21206** | BES | Germany | N54.49 W10.20 | F | 13/08/2015 | Y | - | Y |
| **21216** | BES | Germany | N55.15 W9.90 | F | 09/08/2015 | - | - | Y |
| **21217** | BES | Germany | N55.10 W9.80 | F | 12/08/2015 | - | - | Y |
| **21264** | BES | Germany | N55.12 W9.65 | F | 13/09/2015 | - | - | Y |
| **21281** | BES | Germany | N54.47 W10.24 | F | 01/08/2015 | - | - | Y |
| **21299** | BES | Germany | N54.80 W10.00 | F | 25/07/2015 | - | - | Y |
| **21301** | BES | Germany | N54.40 W10.90 | F | 25/08/2015 | - | - | Y |
| **21458** | BES | Germany | N54.47 W10.34 | M | 14/02/2016 | - | - | - |
| **21484** | BES | Germany | N54.79 W9.44 | F | 09/03/2016 | - | - | Y |
| **21495** | BES | Germany | N54.37 W10.99 | M | 06/04/2016 | - | - | Y |
| **21614** | BES | Germany | N54.32 W10.67 | F | 31/05/2016 | - | - | Y |
| **21770** | BES | Germany | N54.44 W10.18 | F | 21/08/2016 | - | - | Y |
| **22167** | BES | Germany | N54.41 W10.41 | F | 23/10/2016 | - | - | Y |
| **22499** | BES | Germany | N54.44 W11.29 | F | 07/03/2017 | - | - | Y |
| **22589** | BES | Germany | N54.40 W10.21 | M | 20/05/2017 | - | - | Y |
| **22663** | BES | Germany | N54.99 W10.79 | F | 28/06/2017 | - | - | Y |
| **23124** | BES | Germany | N54.02 W10.76 | F | 24/05/2018 | - | - | Y |
| **23126** | BES | Germany | N54.40 W11.31 | M | 09/02/2018 | - | - | Y |
| **23127** | BES | Germany | N54.50 W10.34 | F | 28/03/2018 | - | - | Y |
| **23268** | BES | Germany | N54.84 W9.50 | F | 07/06/2018 | - | - | Y |
| **23817** | BES | Germany | N54.50 W10.30 | F | 25/02/2019 | - | - | Y |
| **24144** | BES | Germany | N54.14 W10.95 | F | 10/08/2019 | - | - | Y |
| **24365** | BES | Germany | N54.55 W10.33 | M | 22/07/2019 | - | - | Y |
| **24393** | BES | Germany | N54.80 W9.43 | F | 17/12/2019 | - | - | Y |
| **24586** | BES | Germany | N54.33 W10.82 | M | 17/03/2020 | - | - | Y |
| **24597** | BES | Germany | N54.14 W10.96 | M | 12/11/2019 | - | - | Y |
| **24685** | BES | Germany | N54.34 W11.07 | M | 21/04/2020 | - | - | Y |
| **24711** | BES | Germany | N54.58 W10.30 | M | 07/03/2020 | - | - | Y |
| **24822** | BES | Germany | N54.58 W10.27 | M | 01/08/2020 | - | - | Y |
| **25159** | BES | Germany | N54.14 W10.96 | M | 19/11/2020 | - | - | Y |
| **25311** | BES | Germany | N54.06 W10.77 | F | 09/12/2020 | - | - | Y |
| **25315** | BES | Germany | N54.50 W11.22 | M | 30/11/2020 | - | - | Y |
| **25326** | BES | Germany | N54.77 W10.00 | M | 11/11/2020 | - | - | Y |
| **25399** | BES | Germany | N54.50 W10.09 | F | - | - | - | Y |
| **25400** | BES | Germany | N54.52 W10.55 | F | 14/01/2021 | - | - | Y |
| **25515** | BES | Germany | N54.44 W10.31 | F | 05/06/2021 | - | - | Y |
| **25527** | BES | Germany | N54.01 W10.77 | F | 01/05/2021 | - | - | Y |
| **26076** | BES | Germany | N54.58 W10.23 | M | 23/11/2021 | - | - | Y |
| **26098** | BES | Germany | N54.15 W10.98 | M | 16/11/2021 | - | - | Y |
| **26174** | BES | Germany | N54.60 W10.20 | M | 08/02/2022 | - | - | Y |
| **26410** | BES | Germany | N54.76 W9.85 | M | 18/04/2022 | - | - | Y |
| **26724** | BES | Germany | N54.44 W10.33 | M | 20/12/2021 | - | - | Y |
| **96_011** | SKA | Denmark | N57.58 W10.10 | F | 01/08/2013 | - | - | Y |
| **96_012** | SKA | Denmark | N57.37 W9.68 | F | 01/08/2013 | Y | - | Y |
| **97_002** | KAT | Denmark | N55.93 W11.50 | F | 01/04/1997 | - | - | Y |
| **97_003** | KAT | Denmark | N55.94 W11.40 | F | 02/04/1997 | - | - | Y |
| **97_004** | KAT | Denmark | N55.98 W11.38 | F | 03/04/1997 | - | - | Y |
| **97_006** | BES | Denmark | N55.32 W10.85 | F | 19/05/1997 | - | Y | Y |
| **97_007** | KAT | Denmark | N56.62 W10.38 | F | 19/06/1997 | - | Y | Y |
| **97_016** | BES | Denmark | N54.93 W12.03 | F | 01/04/1997 | - | - | Y |
| **97_032** | BES | Denmark | N55.43 W10.80 | F | 01/11/1997 | - | Y | Y |
| **97_038** | BES | Denmark | N55.40 W9.68 | F | 08/09/1997 | - | - | Y |
| **97-034** | BES | Denmark | N55.00 W10.15 | F | 01/08/1997 | Y | - | Y |
| **98_014** | KAT | Denmark | N55.94 W11.32 | F | 01/04/1998 | - | - | Y |
| **98_053** | BES | Denmark | N54.82 W12.13 | F | 27/10/1998 | - | - | Y |
| **98-028** | BES | Denmark | N55.32 W11.14 | F | 22/08/1998 | Y | - | Y |
| **A1973-00210** | PBS | Sweden | N65.60 W23.00 | F | 16/09/1973 | - | - | Y |
| **A1975-00016** | PBS | Sweden | N60.60 W18.05 | F | 01/01/1975 | - | - | Y |
| **A1978-05004** | BES | Sweden | N55.36 W12.55 | F | 22/12/1977 | - | - | Y |
| **A1978-05045** | BES-PBS | Sweden | N55.40 W14.26 | M | 25/04/1978 | - | - | Y |
| **A1978-05083** | BES-PBS | Sweden | N55.70 W14.24 | F | 29/07/1978 | - | - | Y |
| **A1979-05171** | BES-PBS | Sweden | N55.47 W14.38 | M | 29/11/1979 | - | - | Y |
| **A1981-05089** | BES-PBS | Sweden | N55.60 W14.33 | F | 05/10/1981 | - | - | Y |
| **A1984-05019** | BES-PBS | Sweden | N55.80 W14.23 | F | 10/03/1984 | - | - | Y |
| **A1984-05020** | BES-PBS | Sweden | N55.85 W14.25 | M | 10/03/1984 | - | - | Y |
| **A1984-05044** | BES | Sweden | N55.50 W12.30 | M | 06/05/1984 | - | - | Y |
| **A1984-05047** | BES-PBS | Sweden | N55.90 W14.28 | M | 23/05/1984 | - | - | - |
| **A1984-05062** | BES | Sweden | N55.50 W12.90 | M | 05/07/1984 | Y | - | Y |
| **A1984-05087** | PBS | Sweden | N57.40 W16.80 | M | 29/07/1984 | Y | - | Y |
| **A1984-05187** | BES-PBS | Sweden | N55.40 W13.65 | M | 16/10/1984 | - | - | Y |
| **A1985-05073** | PBS | Sweden | N56.40 W16.30 | M | 14/04/1985 | - | - | Y |
| **A1985-05179** | BES-PBS | Sweden | N55.65 W14.40 | F | 09/01/1985 | - | - | Y |
| **A1985-05196** | BES-PBS | Sweden | N55.65 W14.30 | F | 17/10/1985 | - | - | Y |
| **A1985-05223** | BES | Sweden | N55.60 W12.90 | M | 24/11/1985 | - | - | Y |
| **A1986-05044** | PBS | Sweden | N56.00 W14.75 | F | 11/05/1986 | - | - | Y |
| **A1986-05132** | BES-PBS | Sweden | N55.75 W14.30 | F | 29/08/1986 | - | - | Y |
| **A1986-05214** | BES-PBS | Sweden | N55.40 W13.84 | M | 08/11/1986 | Y | - | Y |
| **A1986-05229** | PBS | Sweden | N56.00 W14.83 | M | 12/11/1986 | Y | - | Y |
| **A1987-05045** | BES | Sweden | N55.70 W13.00 | F | 27/04/1987 | - | - | Y |
| **A1987-05217** | BES-PBS | Sweden | N55.40 W13.70 | M | 31/07/1987 | - | - | Y |
| **A1987-05226** | PBS | Sweden | N56.06 W15.70 | M | 13/08/1987 | Y | - | Y |
| **A1987-05357** | BES-PBS | Sweden | N55.35 W13.72 | F | 02/12/1987 | - | - | Y |
| **A1988-05010** | PBS | Sweden | N55.90 W15.05 | F | 20/02/1988 | - | - | Y |
| **A1988-05094** | BES | Sweden | N55.70 W12.90 | M | 26/05/1988 | - | - | Y |
| **A1988-05142** | BES | Sweden | N55.35 W13.25 | M | 21/07/1988 | Y | - | Y |
| **A1989-05156** | BES | Sweden | N55.45 W12.90 | F | 03/10/1989 | - | - | Y |
| **A1993-05152** | PBS | Sweden | N56.00 W15.20 | F | 22/06/1993 | - | - | Y |
| **A1993-05153** | PBS | Sweden | N56.10 W14.79 | M | 22/06/1993 | Y | Y | Y |
| **A1993-05175** | PBS | Sweden | N56.50 W16.25 | M | 05/08/1993 | Y | - | Y |
| **A1994-05053** | PBS | Sweden | N57.00 W16.60 | M | 20/01/1994 | - | - | Y |
| **A1994-05175** | BES-PBS | Sweden | N55.75 W14.20 | M | 26/09/1994 | - | - | Y |
| **A1995-05049** | BES-PBS | Sweden | N56.00 W14.50 | - | 27/05/1995 | Y | - | Y |
| **A1997-05187** | PBS | Sweden | N56.10 W14.86 | F | 05/05/1996 | Y | - | Y |
| **A1997-05190** | PBS | Sweden | N58.00 W18.30 | F | 22/01/1997 | Y | - | Y |
| **A1998-05770** | PBS | Sweden | N56.10 W16.20 | F | 16/01/1998 | - | - | Y |
| **A2005-05129** | SKA | Sweden | N58.50 W11.15 | F | 08/07/2004 | Y | - | Y |
| **A2006-05040** | BES-PBS | Sweden | N55.35 W13.50 | F | 03/11/2005 | - | - | Y |
| **A2006-05202** | SKA | Sweden | N57.50 W11.90 | F | 02/08/2006 | - | - | Y |
| **A2006-05226** | BES-PBS | Sweden | N55.80 W14.20 | F | 02/09/2006 | - | - | Y |
| **A2008-05348** | BES-PBS | Sweden | N55.35 W13.55 | M | 11/08/2008 | - | - | Y |
| **A2008-05357** | BES | Sweden | N55.65 W12.80 | F | 31/08/2008 | - | - | - |
| **A2016-05647** | SKA | Sweden | N57.10 W12.10 | - | 19/08/2016 | Y | - | Y |
| **A2016-05697** | BES | Sweden | N55.63 W12.95 | M | 14/09/2016 | - | - | Y |
| **A2016-05698** | BES | Sweden | N55.53 W12.80 | F | 15/09/2016 | Y | - | Y |
| **A2018-05712** | BES | Sweden | N55.21 W13.11 | M | 05/09/2018 | - | Y | - |
| **A2019-05574** | SKA | Sweden | N57.55 W11.80 | F | 11/06/2019 | Y | - | Y |
| **A2019-05576** | SKA | Sweden | N59.00 W10.90 | F | 14/07/2019 | - | - | Y |
| **A2019-05577** | BES-PBS | Sweden | N55.93 W14.43 | M | 06/08/2019 | - | - | Y |
| **A2019-05579** | BES-PBS | Sweden | N55.38 W13.58 | M | 11/08/2019 | - | - | - |
| **A2019-05583** | BES-PBS | Sweden | N55.50 W14.40 | F | 01/09/2019 | - | - | Y |
| **A2019-05584** | BES | Sweden | N55.48 W13.00 | M | 12/09/2019 | - | - | Y |
| **A2020-05730** | BES | Sweden | N55.55 W12.70 | F | 25/08/2020 | - | - | Y |
| **A2020-05731** | BES-PBS | Sweden | N55.40 W14.00 | F | 29/08/2020 | Y | Y | Y |
| **A2020-05732** | BES-PBS | Sweden | N55.55 W14.35 | F | 09/09/2020 | - | - | - |
| **A2020-05733** | BES-PBS | Sweden | N55.40 W13.90 | M | 12/09/2020 | Y | - | Y |
| **A2021-05265** | BES | Sweden | N55.20 W13.22 | F | 26/10/2020 | - | - | Y |
| **A2021-05266** | BES | Sweden | N55.20 W13.16 | F | 26/10/2020 | - | - | - |
| **A2021-05335** | BES | Sweden | N55.40 W13.20 | F | 08/03/2021 | - | - | Y |
| **A2021-08109** | BES-PBS | Sweden | N55.39 W13.55 | F | 20/09/2021 | - | - | Y |
| **A2021-08110** | BES-PBS | Sweden | N55.43 W13.94 | F | 06/10/2021 | - | - | Y |
| **A2021-08113** | BES | Sweden | N55.36 W13.44 | F | 24/09/2021 | - | Y | Y |
| **A2021-08114** | BES | Sweden | N55.39 W12.70 | M | 02/10/2021 | - | - | Y |
| **A2021-08174** | BES | Sweden | N55.67 W13.06 | F | 11/11/2021 | - | - | Y |
| **A2021-08176** | PBS | Sweden | N56.51 W16.37 | M | 06/11/2021 | - | - | Y |
| **A2021-08212** | BES | Sweden | N55.40 W12.96 | M | 16/09/2021 | - | - | Y |
| **A2021-08213** | BES | Sweden | N55.34 W13.28 | F | 08/08/2020 | - | - | Y |
| **A2021-08214** | BES | Sweden | N55.38 W12.92 | F | 07/08/2020 | - | Y | - |
| **A2021-08215** | BES | Sweden | N55.39 W12.85 | M | 16/08/2020 | - | - | Y |
| **A2022-00043** | SKA | Sweden | N57.00 W12.28 | F | 28/01/2022 | - | - | Y |
| **A2022-00291** | SKA | Sweden | N58.07 W11.47 | F | 06/03/2022 | - | - | Y |
| **A2022-00292** | BES | Sweden | N55.45 W12.54 | M | 20/03/2022 | - | - | Y |
| **A2022-00293** | BES | Sweden | N55.46 W12.43 | F | 21/03/2022 | - | - | Y |
| **A2022-00294** | BES | Sweden | N55.46 W12.53 | F | 30/03/2022 | - | - | Y |
| **A2022-00295** | KAT | Sweden | N56.88 W12.45 | F | 06/04/2022 | - | - | Y |
| **A2022-00296** | KAT | Sweden | N55.90 W12.77 | F | 11/04/2022 | - | - | Y |
| **A2022-00297** | KAT | Sweden | N55.85 W12.77 | M | 11/04/2022 | - | - | Y |
| **A2022-00298** | KAT | Sweden | N55.80 W12.70 | M | 11/04/2022 | - | - | Y |
| **A2022-00299** | KAT | Sweden | N55.95 W12.77 | M | 11/04/2022 | - | - | Y |
| **A2022-00300** | KAT | Sweden | N56.80 W12.60 | F | 25/04/2022 | - | - | Y |
| **A2022-00301** | KAT | Sweden | N55.97 W12.75 | F | 02/05/2022 | Y | - | Y |
| **A2022-00307** | SKA | Sweden | N58.07 W11.47 | M | 11/05/2022 | - | - | Y |
| **A2022-00308** | SKA | Sweden | N58.10 W11.18 | M | 11/05/2022 | - | - | Y |
| **A2022-00309** | SKA | Sweden | N58.04 W11.18 | F | 11/05/2022 | - | - | Y |
| **A2022-00428** | KAT | Sweden | N55.78 W12.89 | F | 03/07/2022 | - | - | Y |
| **A2022-00461** | SKA | Sweden | N57.95 W11.54 | M | 15/08/2022 | - | - | Y |
| **A2022-00464** | PBS | Sweden | N56.15 W15.05 | F | 22/08/2022 | - | - | - |
| **A2022-00477** | SKA | Sweden | N58.59 W11.10 | M | 21/09/2022 | - | - | Y |
| **A2022-00483** | SKA | Sweden | N58.59 W11.20 | - | 26/09/2022 | - | - | Y |
| **A2022-00503** | SKA | Sweden | N58.60 W11.20 | - | 03/10/2022 | - | - | Y |
| **A2022-00504** | SKA | Sweden | N58.59 W11.23 | - | 04/10/2022 | - | Y | Y |
| **A2022-00561** | - | - | - | - | - | - | - | Y |
| **A2022-00649** | BES | Sweden | N55.38 W13.05 | F | 08/11/2022 | - | - | Y |
| **B07-17** | BES-PBS | Germany | N54.32 W13.72 | F | 23/09/2015 | - | - | Y |
| **B10-15** | BES | Germany | N54.30 W12.30 | F | 05/09/2014 | - | - | Y |
| **B11-15** | BES | Germany | N54.30 W11.90 | F | 10/09/2014 | - | - | - |
| **B12-15** | BES | Germany | N54.34 W12.00 | F | 27/05/2015 | - | - | Y |
| **B18-13** | BES | Germany | N54.53 W12.54 | F | 11/07/2013 | - | - | Y |
| **B20-15** | BES | Germany | N54.42 W11.85 | M | 18/06/2015 | - | - | - |
| **B22-13** | BES | Germany | N54.30 W12.05 | M | 10/07/2013 | - | - | Y |
| **B22-15** | BES | Germany | N54.49 W12.00 | M | 25/06/2015 | Y | - | Y |
| **B23-15** | BES | Germany | N54.26 W12.23 | F | 01/07/2015 | Y | - | Y |
| **B23a-15** | BES | Germany | N54.40 W12.20 | M | 01/07/2015 | - | - | Y |
| **B26-15** | BES | Germany | N54.40 W11.80 | M | 24/07/2015 | - | - | Y |
| **B30-12** | BES | Germany | N54.55 W13.10 | M | 05/10/2012 | - | - | Y |
| **B32-15** | BES | Germany | N54.28 W12.29 | F | 28/08/2015 | - | - | Y |
| **B33-13** | BES | Germany | N54.30 W12.10 | F | 02/09/2013 | - | - | Y |
| **B34-13** | BES | Germany | N54.60 W11.37 | F | 03/09/2013 | Y | - | Y |
| **B37-16** | BES | Germany | N54.35 W12.38 | F | 08/08/2016 | - | - | Y |
| **B38-15** | BES | Germany | N54.50 W12.40 | F | 25/06/2015 | - | - | - |
| **B40-16** | BES | Germany | N54.20 W12.00 | F | 23/08/2016 | - | - | - |
| **B41-14** | BES | Germany | N54.50 W12.00 | F | 02/07/2014 | Y | - | Y |
| **B43-15** | BES | Germany | N54.50 W12.66 | M | 08/10/2015 | Y | - | Y |
| **B44-16** | BES | Germany | N54.18 W12.11 | M | 02/09/2016 | - | - | - |
| **B47-15** | BES-PBS | Germany | N54.58 W13.58 | M | 09/11/2015 | - | - | Y |
| **B47-16** | BES | Germany | N54.67 W13.30 | F | 06/07/2016 | - | - | Y |
| **B49-16** | BES | Germany | N54.18 W12.01 | M | 22/09/2015 | - | - | - |
| **B50-14** | BES | Germany | N54.50 W12.50 | F | 15/07/2014 | Y | - | Y |
| **B50-16** | BES-PBS | Germany | N54.70 W13.50 | F | 01/07/2016 | - | - | Y |
| **B53-16** | BES | Germany | N54.20 W11.60 | M | 14/08/2016 | - | - | Y |
| **B56-16** | BES-PBS | Germany | N54.04 W14.04 | M | 18/09/2016 | - | - | Y |
| **B58-14** | BES | Germany | N54.15 W11.80 | F | 23/09/2014 | - | - | - |
| **B58-15** | BES | Germany | N54.70 W13.36 | F | 28/09/2015 | Y | - | Y |
| **B59-16** | BES | Germany | N54.15 W11.68 | F | 26/07/2016 | - | - | Y |
| **B61-14** | BES | Germany | N55.00 W13.10 | F | 27/08/2014 | - | - | - |
| **B63-16** | BES | Germany | N54.28 W12.29 | M | 02/12/2016 | - | - | Y |
| **B68-16** | BES | Germany | N54.45 W11.00 | F | 27/07/2016 | - | - | Y |
| **B71-16** | BES | Germany | N54.67 W13.27 | F | 28/12/2016 | - | - | Y |
| **B74-14** | BES | Germany | N55.00 W13.00 | F | 19/09/2014 | - | - | Y |
| **B75-14** | BES | Germany | N54.70 W13.13 | F | 19/09/2014 | Y | - | Y |
| **B85-14** | BES | Germany | N54.90 W12.90 | M | 19/09/2014 | - | - | Y |
| **C1979-05074** | PBS | Sweden | N58.00 W18.00 | M | 15/06/1979 | - | - | Y |
| **C1988-05707** | PBS | Sweden | N56.08 W14.96 | F | 30/05/1988 | Y | - | Y |
| **C1989-05718** | BES | Sweden | N55.52 W12.60 | F | 14/06/1989 | - | - | Y |
| **C1989-05720** | PBS | Sweden | N56.15 W16.90 | F | 06/09/1989 | Y | - | Y |
| **C1990-03048** | PBS | Sweden | N56.05 W15.40 | M | 14/09/1990 | - | - | Y |
| **C1990-03078** | BES-PBS | Sweden | N55.30 W13.60 | M | 15/12/1990 | - | - | Y |
| **C1990-03080** | PBS | Sweden | N57.20 W16.70 | F | 03/01/1991 | - | - | Y |
| **C1990-03105** | BES-PBS | Sweden | N55.90 W14.40 | M | 13/03/1991 | Y | - | Y |
| **C1990-03169** | BES-PBS | Sweden | N55.90 W14.45 | F | 13/05/1991 | - | - | Y |
| **C1990-05746** | BES-PBS | Sweden | N56.00 W14.55 | M | 13/10/1990 | - | Y | Y |
| **C1990-05788** | SKA | Sweden | N59.00 W10.60 | M | 30/07/1990 | - | - | Y |
| **C1990-05796** | SKA | Sweden | N59.00 W10.50 | F | 02/08/1990 | - | Y | Y |
| **C1990-05805** | BES-PBS | Sweden | N55.35 W13.70 | M | 05/10/1990 | - | - | Y |
| **C1991-04044** | PBS | Sweden | N56.00 W15.00 | M | 01/10/1991 | Y | - | Y |
| **C1991-04045** | BES-PBS | Sweden | N55.37 W13.74 | M | 10/10/1991 | - | - | Y |
| **C1991-05348** | SKA | Sweden | N58.00 W11.00 | F | 02/05/1991 | - | - | Y |
| **C1991-05349** | SKA | Sweden | N58.00 W11.10 | M | 06/05/1991 | - | - | Y |
| **C1991-05350** | SKA | Sweden | N58.05 W11.00 | M | 06/05/1991 | - | - | Y |
| **C1991-05364** | SKA | Sweden | N58.60 W11.05 | F | 03/07/1991 | - | - | Y |
| **C2004-5008** | PBS | Latvia | N57.00 W20.40 | F | 15/10/2003 | Y | - | Y |
| **C2004-5009** | PBS | Latvia | N57.00 W20.00 | M | 31/01/2004 | Y | - | Y |
| **C2005-05350** | KAT | Sweden | N56.50 W12.00 | F | 06/08/2005 | - | - | Y |
| **C2005-05354** | KAT | Sweden | N56.45 W12.00 | F | 16/08/2005 | - | - | Y |
| **C2005-05361** | SKA | Sweden | N57.15 W12.00 | - | 01/09/2004 | - | - | Y |
| **C2005-05362** | KAT | Sweden | N56.65 W12.85 | F | 01/06/2005 | - | - | Y |
| **C2005-05363** | KAT | Sweden | N56.55 W12.00 | F | 01/09/2005 | - | - | Y |
| **C2005-05364** | SKA | Sweden | N58.10 W11.20 | F | 16/09/2005 | - | - | - |
| **C2005-05366** | SKA | Sweden | N57.48 W11.70 | - | 04/08/2005 | - | - | Y |
| **C2005-05373** | KAT | Sweden | N56.70 W12.00 | F | 02/09/2004 | - | - | Y |
| **C2006-05296** | SKA | Sweden | N58.70 W11.10 | - | 04/05/2006 | - | - | Y |
| **C2006-05297** | SKA | Sweden | N58.65 W11.00 | - | 17/06/2006 | - | - | Y |
| **C2006-05298** | KAT | Sweden | N56.55 W12.10 | - | 24/07/2006 | - | Y | Y |
| **C2006-05301** | KAT | Sweden | N56.45 W12.20 | - | 31/07/2006 | - | - | Y |
| **C2006-05308** | SKA | Sweden | N58.71 W11.05 | - | 13/09/2006 | - | - | Y |
| **C2007-07619** | KAT | Sweden | N56.60 W12.10 | F | 03/09/2007 | - | - | Y |
| **C2007-07622** | KAT | Sweden | N56.40 W12.20 | F | 06/09/2007 | - | - | - |
| **C2007-07717** | SKA | Sweden | N58.20 W11.15 | - | 20/09/2007 | - | - | Y |
| **C2007-08047** | SKA | Sweden | N58.50 W11.10 | M | 23/09/2007 | Y | - | Y |
| **C2007-08049** | SKA | Sweden | N58.90 W10.70 | - | 10/08/2007 | - | - | - |
| **C2008-00017** | BES | Sweden | N55.55 W12.32 | M | 23/03/2008 | - | Y | Y |
| **C2008-08411** | SKA | Sweden | N57.15 W12.20 | F | 18/07/2008 | - | Y | Y |
| **C2008-08857** | SKA | Sweden | N57.05 W12.08 | - | 20/07/2008 | - | Y | Y |
| **C2008-08861** | KAT | Sweden | N56.20 W12.20 | F | 03/03/2008 | - | - | Y |
| **C2008-08864** | KAT | Sweden | N56.10 W12.10 | F | 06/05/2008 | - | - | Y |
| **C2008-09130** | BES | Sweden | N55.45 W12.20 | F | 24/12/2008 | - | Y | Y |
| **C2009-02664** | SKA | Sweden | N57.90 W11.00 | - | 01/06/2009 | - | Y | Y |
| **C2009-02665** | SKA | Sweden | N56.98 W12.00 | F | 06/07/2009 | - | Y | Y |
| **C2009-04448** | SKA | Sweden | N58.25 W11.15 | - | 08/06/2009 | - | - | Y |
| **C2009-04450** | BES | Sweden | N55.35 W12.90 | - | 25/08/2009 | Y | - | Y |
| **C2009-04451** | BES | Sweden | N55.23 W12.56 | - | 02/10/2009 | - | - | Y |
| **C2009-04460** | SKA | Sweden | N57.45 W11.85 | - | 28/04/2009 | - | - | Y |
| **C2009-04463** | SKA | Sweden | N58.55 W11.00 | - | 16/04/2009 | - | - | Y |
| **C2009-04464** | SKA | Sweden | N57.95 W11.00 | - | 25/07/2009 | - | Y | Y |
| **C2009-04466** | BES-PBS | Sweden | N56.00 W14.65 | - | 21/08/2009 | - | - | - |
| **C2010-10094** | SKA | Sweden | N58.00 W10.90 | F | 22/07/2010 | - | - | Y |
| **C2010-10096** | SKA | Sweden | N57.10 W11.90 | F | 02/08/2010 | - | - | Y |
| **C2010-10097** | SKA | Sweden | N57.00 W12.15 | F | 19/10/2010 | - | - | Y |
| **C2012-00001** | BES | Sweden | N55.58 W12.35 | F | 27/05/2011 | - | - | Y |
| **C2012-00004** | BES | Sweden | N55.50 W12.70 | F | 17/08/2011 | - | - | - |
| **C2012-00014** | SKA | Sweden | N58.15 W11.00 | - | 15/04/2011 | - | - | Y |
| **C2012-00015** | SKA | Sweden | N58.45 W11.00 | - | 28/07/2011 | - | - | Y |
| **C2012-00016** | SKA | Sweden | N59.00 W10.85 | - | 14/05/2011 | Y | - | Y |
| **C2013-07068** | SKA | Sweden | N57.40 W11.80 | - | 06/04/2012 | - | - | Y |
| **C2013-07070** | SKA | Sweden | N58.00 W11.20 | - | 20/08/2013 | - | - | Y |
| **C2013-07071** | SKA | Sweden | N57.10 W11.95 | - | 21/08/2013 | - | - | Y |
| **C2013-07073** | KAT | Sweden | N56.70 W12.60 | - | 04/09/2013 | - | - | Y |
| **C2013-07074** | KAT | Sweden | N56.10 W12.60 | - | 12/04/2013 | - | - | Y |
| **C2015-05289** | SKA | Sweden | N57.00 W12.00 | - | 02/07/2015 | - | - | Y |
| **G1988-07552** | SKA | Sweden | N59.00 W11.00 | F | 14/07/1988 | - | - | Y |
| **G1988-07656** | BES-PBS | Sweden | N55.35 W13.64 | M | 20/09/1988 | - | - | Y |
| **G1988-07719** | PBS | Sweden | N57.30 W16.60 | M | 17/11/1988 | - | - | Y |
| **G1989-07863** | PBS | Sweden | N56.10 W14.90 | M | 13/04/1989 | Y | - | Y |
| **G1989-07869** | PBS | Sweden | N56.00 W15.10 | M | 22/04/1989 | - | - | Y |
| **G2002-05694** | SKA | Sweden | N57.00 W12.00 | F | 26/06/2002 | - | - | Y |
| **M002-22** | BES | Germany | N54.18 W12.01 | F | 01/02/2022 | - | - | Y |
| **M002-23** | BES | Germany | N54.67 W13.27 | F | 05/02/2022 | - | - | Y |
| **M009-19** | BES | Germany | N54.38 W12.41 | F | 18/03/2018 | - | - | Y |
| **M010-23** | BES | Germany | N54.40 W12.44 | F | 15/09/2022 | - | - | Y |
| **M012-19** | BES | Germany | N54.39 W12.44 | F | 27/12/2018 | - | - | Y |
| **M012-23** | BES-PBS | Germany | N54.40 W13.63 | F | 20/08/2022 | - | - | Y |
| **M013-19** | BES | Germany | N54.44 W12.70 | F | 26/01/2018 | - | - | - |
| **M013-23** | BES-PBS | Germany | N54.40 W13.63 | M | 08/07/2022 | - | - | Y |
| **M017-21** | BES | Germany | N54.38 W12.42 | M | 16/03/2021 | - | - | Y |
| **M017-22** | BES-PBS | Germany | N54.40 W13.61 | F | 01/09/2021 | - | Y | Y |
| **M019-21** | BES | Germany | N54.42 W12.46 | F | 13/05/2021 | - | - | Y |
| **M019-23** | BES | Germany | N54.30 W12.34 | F | 27/04/2023 | - | - | Y |
| **M022-23** | BES | Germany | N54.33 W12.37 | F | 08/05/2023 | - | - | - |
| **M027-18** | BES | Germany | N54.37 W12.42 | M | 13/09/2017 | - | - | Y |
| **M031-18** | BES | Germany | N54.43 W12.47 | F | 31/05/2018 | - | - | Y |
| **M031-21** | BES-PBS | Germany | N54.02 W14.07 | M | 05/06/2021 | - | - | - |
| **M035-23** | BES | Germany | N54.52 W13.08 | M | 25/05/2023 | - | - | Y |
| **M039-22** | BES | Germany | N54.35 W12.39 | M | 28/05/2022 | - | - | Y |
| **M040-22** | BES | Germany | N54.35 W12.39 | F | 28/05/2022 | - | Y | Y |
| **M043-20** | BES | Germany | N54.38 W12.42 | F | 05/05/2020 | - | - | - |
| **M045-20** | BES | Germany | N54.67 W13.30 | M | 13/07/2020 | - | - | - |
| **M046-20** | BES | Germany | N54.57 W13.46 | F | 10/07/2020 | - | - | - |
| **M048-20** | BES-PBS | Germany | N54.29 W13.69 | M | 01/07/2020 | - | - | - |
| **M053-21** | BES | Germany | N54.46 W12.55 | M | 03/06/2021 | - | - | - |
| **M054-18** | BES | Germany | N54.38 W12.41 | M | 21/05/2017 | - | - | - |
| **M058-19** | BES | Germany | N54.66 W13.26 | M | 09/07/2019 | - | - | Y |
| **M060-22** | BES | Germany | N54.34 W12.38 | M | 05/07/2022 | - | - | - |
| **M061-22** | BES | Germany | N54.39 W12.53 | M | 13/09/2021 | - | - | Y |
| **M063-21** | BES-PBS | Germany | N54.46 W13.57 | M | 12/07/2021 | - | - | Y |
| **M063-22** | BES-PBS | Germany | N54.36 W13.72 | F | 28/06/2022 | - | - | Y |
| **M066-21** | BES-PBS | Germany | N54.34 W13.75 | M | 10/07/2021 | - | - | - |
| **M066-22** | BES | Germany | N54.31 W12.35 | F | 09/07/2022 | - | - | Y |
| **M067-20** | BES | Germany | N54.47 W12.50 | F | 02/07/2020 | - | - | - |
| **M068-20** | BES | Germany | N54.47 W12.50 | M | 02/07/2020 | - | - | - |
| **M068-22** | BES | Germany | N54.30 W12.33 | M | 13/07/2022 | - | - | Y |
| **M069-20** | BES | Germany | N54.43 W12.47 | F | 22/07/2020 | - | - | - |
| **M072-20** | BES | Germany | N54.39 W12.44 | M | 16/07/2020 | - | - | - |
| **M072-22** | BES | Germany | N54.20 W12.15 | M | 13/07/2022 | - | - | Y |
| **M073-22** | BES | Germany | N54.18 W12.02 | F | 06/05/2022 | - | - | Y |
| **M074-18** | BES | Germany | N54.26 W12.24 | F | 06/07/2018 | - | - | Y |
| **M075-18** | BES | Germany | N54.26 W12.25 | M | 06/07/2018 | - | - | - |
| **M080-18** | BES | Germany | N54.31 W12.35 | M | 08/07/2018 | - | - | Y |
| **M080-20** | BES | Germany | N54.67 W13.43 | M | 10/08/2020 | - | - | - |
| **M081-18** | BES | Germany | N54.35 W12.38 | M | 10/07/2018 | - | - | - |
| **M081-20** | BES | Germany | N54.59 W13.41 | M | 11/08/2020 | - | - | - |
| **M082-18** | BES | Germany | N54.35 W12.39 | F | 10/07/2018 | - | - | Y |
| **M083-22** | BES | Germany | N54.35 W12.38 | F | 29/08/2022 | - | - | Y |
| **M085-20** | BES | Germany | N54.64 W13.22 | M | 23/08/2020 | - | - | Y |
| **M087-18** | BES | Germany | N54.43 W12.47 | M | 04/07/2018 | - | - | Y |
| **M087-22** | BES | Germany | N54.61 W13.41 | M | 04/09/2022 | - | - | - |
| **M091-20** | BES | Germany | N54.67 W13.27 | M | 08/09/2020 | - | - | Y |
| **M093-18** | BES | Germany | N54.27 W12.26 | M | 19/07/2018 | - | Y | Y |
| **M094-18** | BES | Germany | N54.30 W12.35 | M | 20/07/2018 | - | - | Y |
| **M095-20** | BES | Germany | N54.67 W13.30 | F | 10/09/2020 | - | - | - |
| **M096-19** | BES-PBS | Germany | N54.51 W13.64 | M | 25/10/2019 | - | - | Y |
| **M097-22** | BES | Germany | N54.68 W13.35 | F | 19/09/2022 | - | - | - |
| **M098-19** | BES | Germany | N54.44 W12.73 | M | 23/07/2019 | - | - | Y |
| **M098-21** | BES | Germany | N54.67 W13.29 | F | 03/08/2021 | - | - | - |
| **M098-22** | BES | Germany | N54.67 W13.44 | F | 21/09/2022 | - | - | Y |
| **M099-18** | BES-PBS | Germany | N54.42 W13.60 | F | 31/07/2018 | - | - | Y |
| **M100-20** | BES | Germany | N54.65 W13.24 | F | 14/09/2020 | - | - | - |
| **M100-22** | BES | Germany | N54.37 W12.41 | F | 29/09/2022 | - | - | Y |
| **M101-18** | BES | Germany | N54.41 W12.45 | F | 06/08/2018 | - | - | - |
| **M101-22** | BES | Germany | N54.26 W12.24 | M | 28/09/2022 | - | - | Y |
| **M104-18** | BES | Germany | N54.67 W13.30 | F | 10/08/2018 | - | Y | - |
| **M108-18** | BES | Germany | N54.34 W12.38 | F | 14/08/2018 | - | Y | Y |
| **M109-18** | BES | Germany | N54.40 W12.45 | F | 20/07/2018 | - | - | Y |
| **M110-18** | BES | Germany | N54.37 W12.41 | M | 22/07/2018 | - | - | Y |
| **M111-18** | BES | Germany | N54.44 W12.75 | F | 22/08/2018 | - | - | Y |
| **M111-21** | BES-PBS | Germany | N54.37 W13.71 | F | 08/06/2021 | - | - | Y |
| **M111-22** | BES | Germany | N54.49 W12.52 | M | 07/10/2020 | - | - | Y |
| **M112-20** | BES-PBS | Germany | N54.12 W13.85 | M | 14/06/2020 | - | - | - |
| **M113-22** | BES | Germany | N54.37 W12.41 | F | 18/08/2021 | - | - | Y |
| **M115-20** | BES | Germany | N54.58 W13.42 | F | 15/10/2020 | - | - | - |
| **M115-22** | BES | Germany | N54.47 W12.50 | F | 28/04/2022 | - | - | Y |
| **M116-20** | BES-PBS | Germany | N54.43 W13.58 | F | 18/10/2020 | - | - | - |
| **M117-20** | BES-PBS | Germany | N54.52 W13.67 | F | 17/10/2020 | - | - | - |
| **M120-18** | BES | Germany | N54.43 W12.47 | M | 20/08/2018 | - | - | Y |
| **M122-18** | BES | Germany | N54.67 W13.31 | M | 22/08/2018 | - | - | - |
| **M122-22** | BES | Germany | N54.44 W12.70 | F | 09/12/2022 | - | - | Y |
| **M123-21** | BES | Germany | N54.67 W13.30 | M | 25/08/2021 | - | - | - |
| **M126-21** | BES | Germany | N54.45 W12.59 | F | 12/07/2021 | - | - | Y |
| **M133-18** | BES | Germany | N54.44 W12.69 | F | 03/09/2018 | - | - | Y |
| **M133-21** | BES | Germany | N54.41 W12.46 | M | 02/08/2021 | - | - | Y |
| **M135-21** | BES | Germany | N54.41 W12.46 | F | 16/08/2021 | - | - | - |
| **M136-18** | BES | Germany | N54.26 W12.24 | M | 13/08/2018 | - | - | - |
| **M138-18** | BES | Germany | N54.67 W13.28 | F | 17/09/2018 | - | - | Y |
| **M142-18** | BES | Germany | N54.30 W12.33 | F | 01/10/2018 | - | - | Y |
| **M142-21** | BES | Germany | N54.45 W12.62 | M | 27/11/2020 | - | - | Y |
| **M144-18** | BES | Germany | N54.30 W12.34 | F | 30/09/2018 | - | - | Y |
| **M153-21** | BES-PBS | Germany | N54.40 W13.61 | M | 20/09/2021 | - | - | - |
| **M172-21** | BES | Germany | N54.46 W13.05 | F | 08/04/2021 | - | - | - |
| **Pol_53** | PBS | Poland | N54.70 W18.60 | M | 01/07/1996 | Y | - | Y |
| **Pol_58** | PBS | Poland | N54.50 W18.65 | F | 01/01/1998 | Y | - | Y |
| **Pol_59** | PBS | Poland | N54.45 W18.64 | M | 01/01/1998 | - | - | Y |
| **Pol_60** | PBS | Poland | N55.50 W17.00 | M | 01/12/1999 | - | - | Y |
| **Pol_67** | PBS | Poland | N55.56 W17.63 | M | 01/08/2000 | - | - | Y |
| **Pol_68** | PBS | Poland | N55.30 W17.20 | F | 01/11/2000 | Y | - | Y |
| **Pol_69** | PBS | Poland | N54.73 W18.58 | M | 01/02/2000 | - | - | Y |
| **Pol_71** | PBS | Poland | N54.50 W19.20 | M | 01/03/2001 | - | - | Y |
| **Pol_72** | PBS | Poland | N54.80 W18.58 | F | 01/06/2001 | Y | - | Y |
| **Pol_74** | PBS | Poland | N54.70 W18.62 | F | 01/02/2002 | Y | - | Y |
| **S28** | SKA | Sweden | N57.00 W11.40 | F | 01/06/1990 | - | - | Y |
| **S30** | SKA | Sweden | N57.20 W11.20 | F | 01/05/1991 | - | - | Y |
| **S37** | - | - | - | - | - | - | - | Y |
| **S38** | SKA | Sweden | N57.10 W11.00 | F | 01/05/1991 | - | - | Y |
| **S39** | SKA | Sweden | N57.30 W11.30 | F | 01/05/1991 | - | - | Y |
| **S43** | SKA | Sweden | N57.20 W11.60 | F | 01/06/1990 | - | - | Y |
| **S46** | KAT | Sweden | N56.30 W12.20 | F | 01/04/1991 | - | - | Y |
| **S47** | - | - | - | - | - | - | - | Y |
| **S48** | KAT | Sweden | N56.00 W12.70 | F | 01/05/1990 | - | - | Y |
| **S49** | KAT | Sweden | N56.00 W11.90 | F | 01/07/1990 | - | - | Y |
| **S50** | KAT | Sweden | N56.15 W11.90 | F | 01/07/1991 | - | - | Y |
| **S51** | KAT | Sweden | N56.20 W11.80 | F | 01/07/1990 | - | - | Y |
| **S52** | KAT | Sweden | N56.15 W12.20 | F | 01/07/1990 | - | - | Y |
| **S55** | KAT | Sweden | N56.35 W12.70 | F | 01/05/1990 | - | - | Y |

**Table S3:** Testing scheme in fastsimcoal2 simulations to infer timing and order of splits among the three harbour porpoise populations in North and Baltic Sea. Single split refers to a single split from one ancestral population. Two splits refers to a first split of the ancestral population into two populations, one of which splitting later again. The two most likely scenarios are highlighted in green.

| **Name** | **Number of parameters estimated** | **Gene flow** | **T 1st split (years) prior** | **T 1st split (years) estimated** | **T 2nd split (years) prior** | **T 2nd split (years) estimated** | **Likelihood significantly increased (α<0.01)** | **compared to** | **Outcome** |
| --- | --- | --- | --- | --- | --- | --- | --- | --- | --- |
| **3 populations, single split, colonization restricted to Baltic Sea period (≤ 8,500 years)** | | | | | | | | | |
| P1 | 1 | no | ≤ 8,500 | 7,640 | - | - | - | - | - |
| **3 populations, two splits, colonization restricted to Baltic Sea period (≤ 8,500 years); split order unconstrained** | | | | | | | | | |
| P2a | 2 | no | ≤ 8,500 | 7,902 | ≤ 8,500 | 4,296 | yes | P1 | P1 rejected |
| P6a | 6 | Yes, adjacent pops | ≤ 8,500 | 8,044 | ≤ 8,500 | 5,510 | yes | P2a | P2a rejected |
| P8a | 8 | Yes, all pops | ≤ 8,500 | 8,033 | ≤ 8,500 | 4,379 | yes (marginally), but implausible population estimates | P6a | P6a maintained |
| **3 populations, two splits, colonization restricted to Baltic Sea period (≤ 8,500 years); 1st split NOS vs. (BES,PBS), 2nd split BES vs. PBS** | | | | | | | | | |
| P2b | 2 | no | 8,500-6,000 | 8,116 | ≤ 6,000 | 5,688 | no, significantly decreased | P2a | P2b rejected |
| P6b | 6 | Yes, adjacent pops | 8,500-6,000 | 8,366 | ≤ 6,000 | 5,867 | no, significantly decreased | P6a | P6b rejected |
| **3 populations, two splits, colonization allowed since onset of Yoldia period (≤ 12,000 years) ; split order unconstrained** | | | | | | | | | |
| P2c | 2 | no | ≤ 12,000 | 10,103 | ≤ 12,000 | 4,891 | yes | P1 | P1 rejected |
| P6c | 6 | Yes, adjacent pops | ≤ 12,000 | 11,805 | ≤ 12,000 | 7,854 | yes | P2c | P2c rejected |
| P8c | 8 | Yes, all pops | ≤ 12,000 | 11,460 | ≤ 12,000 | 6,973 | no | P6c | P6c maintained |
| **3 populations, two splits; 1st split NOS vs. (BES,PBS) during Yoldia period, 2nd split BES vs. PBS during Baltic Sea period** | | | | | | | | | |
| P2d | 2 | no | 12,000-10,000 | 11,127 | ≤ 8500 | 7,973 | no, significantly decreased | P2c | P2d rejected |
| P6d | 6 | Yes, adjacent pops | 12,000-10,000 | 11,257 | ≤ 8,500 | 9,211 | no, significantly decreased | P4c | P4d rejected |
| **3 populations, two splits; 1st split PBS vs. (NOS,BES) during Yoldia period, 2nd split NOS vs. BES during Baltic Sea period** | | | | | | | | | |
| P2e | 2 | no | 12,000-10,000 | 11,115 | ≤ 8,500 | 7,140 | no, significantly decreased | P2c | P2d rejected |
| P6e | 6 | Yes, adjacent pops | 12,000-10,000 | 11,091 | ≤ 8,500 | 7,818 | no, significantly decreased | P4c | P4d rejected |

**Table S4:** The 192 selected SNPs for the reduced panel of SNPs that assign samples to any of the three identified Harbour porpoise populations occurring in the Baltic region.

| **SNP_ID** | **Vaquita** **Chr** | **Fst** | **Population**  **comparison** | **Amplification**  **worked** | **Fluidigm-WGS**  **Error Rate** | **Fluidigm-Fluidigm**  **Error Rate** | **Final Reliable** |
| --- | --- | --- | --- | --- | --- | --- | --- |
| **ScshVNz_11693_2368968** | 1 | 0.5416 | NOS_BES | Yes | 7.02 | 6.06 | Yes |
| **ScshVNz_2264_104856** | 1 | 0.6896 | NOS_BES | Yes | 5.26 | 9.09 | Yes |
| **ScshVNz_2264_117251** | 1 | 0.688 | NOS_BES | Yes | 1.75 | 6.06 | Yes |
| **ScshVNz_2264_153571** | 1 | 0.6379 | NOS_BES | Yes | 3.51 | 12.12 | Yes |
| **ScshVNz_2264_186730** | 1 | 0.5829 | NOS_BES | Yes | 3.51 | 6.06 | Yes |
| **ScshVNz_2264_197765** | 1 | 0.5668 | NOS_BES | Yes | 8.77 | 3.03 | Yes |
| **ScshVNz_2264_201718** | 1 | 0.5706 | NOS_BES | Yes | 3.51 | 3.03 | Yes |
| **ScshVNz_2264_214236** | 1 | 0.5348 | NOS_BES | Yes | 8.77 | 3.03 | Yes |
| **ScshVNz_2264_214765** | 1 | 0.5927 | NOS_BES | Yes | 5.26 | 9.09 | Yes |
| **ScshVNz_2264_219370** | 1 | 0.5952 | NOS_BES | Yes | 3.51 | 21.21 | Yes |
| **ScshVNz_2264_228365** | 1 | 0.6471 | NOS_BES | Yes | 5.26 | 6.06 | Yes |
| **ScshVNz_2264_235089** | 1 | 0.6519 | NOS_BES | Yes | 1.75 | 6.06 | Yes |
| **ScshVNz_2264_59096** | 1 | 0.8006 | NOS_BES | Yes | 14.04 | 24.24 | Yes |
| **ScshVNz_7648_4670203** | 1 | 0.8626 | NOS_PBS | Yes | 1.75 | 3.03 | Yes |
| **ScshVNz_8603_8169203** | 1 | 0.935 | NOS_PBS | Yes | 5.26 | 9.09 | Yes |
| **ScshVNz_8603_8197267** | 1 | 0.935 | NOS_PBS | Yes | 5.26 | 12.12 | Yes |
| **ScshVNz_10140_7762930** | 2 | 0.8448 | BES_PBS | Yes | 5.26 | 12.12 | Yes |
| **ScshVNz_10140_9211906** | 2 | 0.8626 | NOS_PBS | Yes | 8.77 | 9.09 | Yes |
| **ScshVNz_3249_21425997** | 2 | 0.8652 | NOS_PBS | Yes | 0 | 3.03 | Yes |
| **ScshVNz_3337_11624956** | 2 | 0.8381 | BES_PBS | Yes | 0 | 0 | Yes |
| **ScshVNz_3337_18362286** | 2 | 0.8652 | NOS_PBS | Yes | 8.77 | 12.12 | Yes |
| **ScshVNz_3337_32561908** | 2 | 0.8222 | NOS_PBS | Yes | 5.26 | 3.03 | Yes |
| **ScshVNz_7569_23942648** | 2 | 0.8373 | BES_PBS | Yes | 0 | 0 | Yes |
| **ScshVNz_7569_24844269** | 2 | 0.8222 | NOS_PBS | Yes | 5.26 | 12.12 | Yes |
| **ScshVNz_7569_26494385** | 2 | 0.8792 | NOS_PBS | Yes | 1.75 | 3.03 | Yes |
| **ScshVNz_9960_13032288** | 2 | 0.8222 | NOS_PBS | Yes | 5.26 | 9.09 | Yes |
| **ScshVNz_9960_13035163** | 2 | 0.8626 | NOS_PBS | Yes | 1.75 | 9.09 | Yes |
| **ScshVNz_1186_12712766** | 3 | 0.8341 | BES_PBS | Yes | 3.51 | 6.06 | Yes |
| **ScshVNz_12231_25005158** | 3 | 0.8286 | BES_PBS | Yes | 1.75 | 3.03 | Yes |
| **ScshVNz_12231_27637037** | 3 | 0.7283 | NOS_BES | Yes | 3.51 | 6.06 | Yes |
| **ScshVNz_12231_31818296** | 3 | 0.7883 | BES_PBS | Yes | 0 | 9.09 | Yes |
| **ScshVNz_7191_10886974** | 3 | 0.8334 | BES_PBS | Yes | 0 | 0 | Yes |
| **ScshVNz_7191_20123107** | 3 | 0.8448 | BES_PBS | Yes | 3.51 | 3.03 | Yes |
| **ScshVNz_5358_5897141** | 4 | 0.5575 | NOS_BES | Yes | 3.51 | 9.09 | Yes |
| **ScshVNz_9797_17574369** | 4 | 0.6456 | NOS_BES | Yes | 12.28 | 9.09 | Yes |
| **ScshVNz_9797_4956315** | 4 | 0.8652 | NOS_PBS | Yes | 3.51 | 3.03 | Yes |
| **ScshVNz_9797_8087479** | 4 | 0.8626 | NOS_PBS | Yes | 0 | 3.03 | Yes |
| **ScshVNz_10772_6906691** | 5 | 0.8712 | NOS_PBS | Yes | 12.28 | 15.15 | Yes |
| **ScshVNz_10772_6948105** | 5 | 0.8626 | NOS_PBS | Yes | 7.02 | 9.09 | Yes |
| **ScshVNz_11919_1518680** | 5 | 0.5308 | NOS_BES | Yes | 5.26 | 3.03 | Yes |
| **ScshVNz_3795_1215616** | 5 | 0.8652 | NOS_PBS | Yes | 5.26 | 3.03 | Yes |
| **ScshVNz_3795_838108** | 5 | 0.8626 | NOS_PBS | Yes | 5.26 | 6.06 | Yes |
| **ScshVNz_8081_567307** | 5 | 0.849 | BES_PBS | Yes | 1.75 | 6.06 | Yes |
| **ScshVNz_1608_11775768** | 6 | 0.8853 | BES_PBS | Yes | 0 | 6.06 | Yes |
| **ScshVNz_515_4123512** | 6 | 0.5303 | NOS_BES | Yes | 5.26 | 3.03 | Yes |
| **ScshVNz_515_581874** | 6 | 0.8106 | NOS_PBS | Yes | 7.02 | 18.18 | Yes |
| **ScshVNz_6515_15528599** | 7 | 0.8589 | BES_PBS | Yes | 3.51 | 6.06 | Yes |
| **ScshVNz_6515_17472779** | 7 | 0.8626 | NOS_PBS | Yes | 12.28 | 12.12 | Yes |
| **ScshVNz_6515_22302010** | 7 | 0.8996 | BES_PBS | Yes | 3.51 | 3.03 | Yes |
| **ScshVNz_6515_7959107** | 7 | 0.8652 | NOS_PBS | Yes | 7.02 | 3.03 | Yes |
| **ScshVNz_6894_19007288** | 7 | 0.8569 | BES_PBS | Yes | 3.51 | 6.06 | Yes |
| **ScshVNz_6894_22832066** | 7 | 0.7936 | NOS_PBS | Yes | 3.51 | 12.12 | Yes |
| **ScshVNz_6894_41661689** | 7 | 0.8222 | NOS_PBS | Yes | 8.77 | 12.12 | Yes |
| **ScshVNz_6894_44836304** | 7 | 0.8448 | BES_PBS | Yes | 5.26 | 9.09 | Yes |
| **ScshVNz_6894_49784492** | 7 | 0.5423 | NOS_BES | Yes | 7.02 | 3.03 | Yes |
| **ScshVNz_6894_6578187** | 7 | 0.8667 | BES_PBS | Yes | 5.26 | 18.18 | Yes |
| **ScshVNz_9857_1841037** | 7 | 0.5479 | NOS_BES | Yes | 1.75 | 0 | Yes |
| **ScshVNz_2312_1986612** | 9 | 0.8745 | BES_PBS | Yes | 3.51 | 15.15 | Yes |
| **ScshVNz_2531_6619325** | 9 | 0.8341 | BES_PBS | Yes | 8.77 | 3.03 | Yes |
| **ScshVNz_6983_8080890** | 9 | 0.5386 | NOS_BES | Yes | 5.26 | 9.09 | Yes |
| **ScshVNz_1183_5701211** | 10 | 0.6436 | NOS_BES | Yes | 1.75 | 6.06 | Yes |
| **ScshVNz_1183_8233847** | 10 | 0.8341 | BES_PBS | Yes | 7.02 | 6.06 | Yes |
| **ScshVNz_8015_13514346** | 10 | 0.5601 | NOS_BES | Yes | 1.75 | 3.03 | Yes |
| **ScshVNz_8015_16045777** | 10 | 0.5301 | NOS_BES | Yes | 1.75 | 0 | Yes |
| **ScshVNz_8015_30948670** | 10 | 0.8652 | NOS_PBS | Yes | 3.51 | 21.21 | Yes |
| **ScshVNz_8015_32744515** | 10 | 0.7845 | BES_PBS | Yes | 0 | 0 | Yes |
| **ScshVNz_8015_39344553** | 10 | 0.8045 | BES_PBS | Yes | 5.26 | 9.09 | Yes |
| **ScshVNz_8015_46006816** | 10 | 0.7787 | BES_PBS | Yes | 5.26 | 3.03 | Yes |
| **ScshVNz_13313_9225276** | 11 | 0.8222 | NOS_PBS | Yes | 0 | 6.06 | Yes |
| **ScshVNz_13313_9241478** | 11 | 0.935 | NOS_PBS | Yes | 5.26 | 24.24 | Yes |
| **ScshVNz_4865_155797** | 11 | 0.8381 | BES_PBS | Yes | 1.75 | 0 | Yes |
| **ScshVNz_4865_6468067** | 11 | 0.8487 | NOS_PBS | Yes | 10.53 | 15.15 | Yes |
| **ScshVNz_77_2036304** | 11 | 0.8381 | BES_PBS | Yes | 1.75 | 3.03 | Yes |
| **ScshVNz_12949_17073540** | 12 | 0.8888 | BES_PBS | Yes | 0 | 0 | Yes |
| **ScshVNz_9860_18278254** | 12 | 0.8448 | BES_PBS | Yes | 5.26 | 3.03 | Yes |
| **ScshVNz_9860_23820149** | 12 | 0.8448 | BES_PBS | Yes | 3.51 | 6.06 | Yes |
| **ScshVNz_9860_23841667** | 12 | 1 | NOS_PBS | Yes | 1.75 | 0 | Yes |
| **ScshVNz_9860_24397844** | 12 | 0.5416 | NOS_BES | Yes | 3.51 | 0 | Yes |
| **ScshVNz_9860_29085321** | 12 | 0.8652 | NOS_PBS | Yes | 5.26 | 6.06 | Yes |
| **ScshVNz_9860_64490643** | 12 | 0.8626 | NOS_PBS | Yes | 5.26 | 9.09 | Yes |
| **ScshVNz_12437_3137058** | 13 | 0.8381 | BES_PBS | Yes | 0 | 6.06 | Yes |
| **ScshVNz_7385_11745008** | 13 | 0.8373 | BES_PBS | Yes | 1.75 | 9.09 | Yes |
| **ScshVNz_7385_11921252** | 13 | 0.8888 | BES_PBS | Yes | 8.77 | 3.03 | Yes |
| **ScshVNz_7385_11922935** | 13 | 0.8626 | NOS_PBS | Yes | 3.51 | 6.06 | Yes |
| **ScshVNz_7385_12053555** | 13 | 0.8098 | BES_PBS | Yes | 0 | 6.06 | Yes |
| **ScshVNz_7385_14034500** | 13 | 0.7883 | BES_PBS | Yes | 0 | 0 | Yes |
| **ScshVNz_7385_15585188** | 13 | 0.8334 | BES_PBS | Yes | 0 | 3.03 | Yes |
| **ScshVNz_7385_16618151** | 13 | 0.7858 | BES_PBS | Yes | 1.75 | 6.06 | Yes |
| **ScshVNz_7385_17059695** | 13 | 0.8246 | BES_PBS | Yes | 3.51 | 6.06 | Yes |
| **ScshVNz_7385_18780951** | 13 | 0.8045 | BES_PBS | Yes | 8.77 | 0 | Yes |
| **ScshVNz_7385_23260443** | 13 | 0.849 | BES_PBS | Yes | 5.26 | 0 | Yes |
| **ScshVNz_7385_23821532** | 13 | 0.8888 | BES_PBS | Yes | 0 | 6.06 | Yes |
| **ScshVNz_7385_28998592** | 13 | 0.8373 | BES_PBS | Yes | 7.02 | 3.03 | Yes |
| **ScshVNz_3285_14936232** | 14 | 0.5306 | NOS_BES | Yes | 0 | 6.06 | Yes |
| **ScshVNz_3285_46518671** | 14 | 0.8381 | BES_PBS | Yes | 1.75 | 3.03 | Yes |
| **ScshVNz_8998_10594912** | 14 | 0.849 | BES_PBS | Yes | 0 | 3.03 | Yes |
| **ScshVNz_8998_12252862** | 14 | 0.8626 | NOS_PBS | Yes | 1.75 | 0 | Yes |
| **ScshVNz_8998_12261636** | 14 | 0.8626 | NOS_PBS | Yes | 0 | 0 | Yes |
| **ScshVNz_8998_9935789** | 14 | 0.8373 | BES_PBS | Yes | 1.75 | 0 | Yes |
| **ScshVNz_10356_3076825** | 15 | 0.6091 | NOS_BES | Yes | 5.26 | 9.09 | Yes |
| **ScshVNz_12150_29886986** | 15 | 0.5779 | NOS_BES | Yes | 3.51 | 0 | Yes |
| **ScshVNz_12150_4820181** | 15 | 0.8896 | BES_PBS | Yes | 24.56 | 0 | Yes |
| **ScshVNz_12150_4831122** | 15 | 0.8996 | BES_PBS | Yes | 0 | 3.03 | Yes |
| **ScshVNz_12150_6226355** | 15 | 0.8652 | NOS_PBS | Yes | 3.51 | 15.15 | Yes |
| **ScshVNz_12150_7175090** | 15 | 0.8381 | BES_PBS | Yes | 1.75 | 0 | Yes |
| **ScshVNz_4275_2137085** | 15 | 0.8865 | BES_PBS | Yes | 1.75 | 0 | Yes |
| **ScshVNz_9915_12349973** | 15 | 0.5414 | NOS_BES | Yes | 7.02 | 9.09 | Yes |
| **ScshVNz_9915_12351875** | 15 | 0.5927 | NOS_BES | Yes | 3.51 | 9.09 | Yes |
| **ScshVNz_9915_12401736** | 15 | 0.602 | NOS_BES | Yes | 1.75 | 3.03 | Yes |
| **ScshVNz_9915_12407007** | 15 | 0.5908 | NOS_BES | Yes | 10.53 | 6.06 | Yes |
| **ScshVNz_9915_12415810** | 15 | 0.602 | NOS_BES | Yes | 0 | 21.21 | Yes |
| **ScshVNz_9915_12464328** | 15 | 0.6597 | NOS_BES | Yes | 1.75 | 18.18 | Yes |
| **ScshVNz_9915_12496798** | 15 | 0.73 | NOS_BES | Yes | 3.51 | 3.03 | Yes |
| **ScshVNz_9915_12513811** | 15 | 0.7748 | NOS_BES | Yes | 7.02 | 24.24 | Yes |
| **ScshVNz_9915_12550024** | 15 | 0.5694 | NOS_BES | Yes | 3.51 | 9.09 | Yes |
| **ScshVNz_9915_12564636** | 15 | 0.592 | NOS_BES | Yes | 3.51 | 6.06 | Yes |
| **ScshVNz_9915_12578828** | 15 | 0.6798 | NOS_BES | Yes | 3.51 | 15.15 | Yes |
| **ScshVNz_9915_12598231** | 15 | 0.6247 | NOS_BES | Yes | 1.75 | 6.06 | Yes |
| **ScshVNz_9915_12606307** | 15 | 0.6331 | NOS_BES | Yes | 0 | 15.15 | Yes |
| **ScshVNz_9915_12621447** | 15 | 0.6331 | NOS_BES | Yes | 0 | 18.18 | Yes |
| **ScshVNz_9915_12633879** | 15 | 0.6565 | NOS_BES | Yes | 3.51 | 6.06 | Yes |
| **ScshVNz_9915_12671138** | 15 | 0.5785 | NOS_BES | Yes | 1.75 | 6.06 | Yes |
| **ScshVNz_9915_4879279** | 15 | 0.5301 | NOS_BES | Yes | 7.02 | 3.03 | Yes |
| **ScshVNz_2616_8767703** | 16 | 0.5607 | NOS_BES | Yes | 12.28 | 18.18 | Yes |
| **ScshVNz_7704_6651459** | 16 | 0.8542 | NOS_PBS | Yes | 3.51 | 6.06 | Yes |
| **ScshVNz_982_48292424** | 16 | 0.8888 | BES_PBS | Yes | 3.51 | 9.09 | Yes |
| **ScshVNz_1753_11481852** | 17 | 0.8626 | NOS_PBS | Yes | 0 | 12.12 | Yes |
| **ScshVNz_4138_2475757** | 17 | 0.6812 | NOS_BES | Yes | 7.02 | 12.12 | Yes |
| **ScshVNz_4138_2483234** | 17 | 0.6789 | NOS_BES | Yes | 7.02 | 6.06 | Yes |
| **ScshVNz_7403_9923425** | 17 | 0.8106 | NOS_PBS | Yes | 7.02 | 9.09 | Yes |
| **ScshVNz_11493_31760905** | 18 | 0.8045 | BES_PBS | Yes | 3.51 | 15.15 | Yes |
| **ScshVNz_11493_44127044** | 18 | 0.8719 | NOS_PBS | Yes | 12.28 | 6.06 | Yes |
| **ScshVNz_9717_3420953** | 19 | 0.8542 | NOS_PBS | Yes | 1.75 | 3.03 | Yes |
| **ScshVNz_2056_18645305** | 20 | 0.9298 | BES_PBS | Yes | 15.79 | 3.03 | Yes |
| **ScshVNz_11897_2549980** | 21 | 0.8626 | NOS_PBS | Yes | 7.02 | 12.12 | Yes |
| **ScshVNz_12865_11432193** | Unplaced | 0.8334 | BES_PBS | Yes | 0 | 0 | Yes |
| **ScshVNz_12865_11453031** | Unplaced | 0.5352 | NOS_BES | Yes | 0 | 9.09 | Yes |
| **ScshVNz_12865_25997452** | Unplaced | 0.8373 | BES_PBS | Yes | 8.77 | 6.06 | Yes |
| **ScshVNz_12865_36299984** | Unplaced | 0.8334 | BES_PBS | Yes | 0 | 6.06 | Yes |
| **ScshVNz_12865_36435016** | Unplaced | 0.8347 | BES_PBS | Yes | 0 | 3.03 | Yes |
| **ScshVNz_12865_36617445** | Unplaced | 0.8853 | BES_PBS | Yes | 0 | 15.15 | Yes |
| **ScshVNz_9900_1059668** | Unplaced | 0.7936 | NOS_PBS | Yes | 8.77 | 0 | Yes |
| **ScshVNz_9900_18738679** | Unplaced | 0.85 | NOS_PBS | Yes | 8.77 | 6.06 | Yes |
| **ScshVNz_9900_25207323** | Unplaced | 0.8542 | NOS_PBS | Yes | 7.02 | 3.03 | Yes |
| **ScshVNz_9900_37628726** | Unplaced | 0.7936 | NOS_PBS | Yes | 5.26 | 6.06 | Yes |
| **ScshVNz_9900_4411903** | Unplaced | 0.8821 | BES_PBS | Yes | 1.75 | 3.03 | Yes |
| **ScshVNz_9900_6083947** | Unplaced | 0.849 | BES_PBS | Yes | 7.02 | 3.03 | Yes |
| **ScshVNz_9900_8669027** | Unplaced | 0.7936 | NOS_PBS | Yes | 10.53 | 9.09 | Yes |
| **ScshVNz_9900_9446313** | Unplaced | 1 | NOS_PBS | Yes | 7.02 | 3.03 | Yes |
| **ScshVNz_2264_6830** | 1 | 0.812 | NOS_PBS | Yes | 7.02 | 33.33 | No |
| **ScshVNz_9960_13060140** | 2 | 0.8542 | NOS_PBS | Yes | 89.47 | 42.42 | No |
| **ScshVNz_12231_24919664** | 3 | 0.5306 | NOS_BES | Yes | 1.75 | 30.3 | No |
| **ScshVNz_10772_14638890** | 5 | 0.8626 | NOS_PBS | Yes | 8.77 | 33.33 | No |
| **ScshVNz_6515_14276257** | 7 | 0.8719 | NOS_PBS | Yes | 77.19 | 12.12 | No |
| **ScshVNz_12947_1165306** | 13 | 0.8222 | NOS_PBS | Yes | 43.86 | 9.09 | No |
| **ScshVNz_12947_5530314** | 13 | 0.8575 | NOS_PBS | Yes | 61.4 | 12.12 | No |
| **ScshVNz_7385_22197235** | 13 | 0.8381 | BES_PBS | Yes | 94.74 | 42.42 | No |
| **ScshVNz_7385_38676569** | 13 | 0.8652 | NOS_PBS | Yes | 5.26 | 42.42 | No |
| **ScshVNz_9915_12429182** | 15 | 0.5905 | NOS_BES | Yes | 8.77 | 39.39 | No |
| **ScshVNz_9915_12678189** | 15 | 0.5433 | NOS_BES | Yes | 64.91 | 0 | No |
| **ScshVNz_10530_4007053** | Unplaced | 0.8626 | NOS_PBS | Yes | 26.32 | 15.15 | No |
| **ScshVNz_2264_101811** | 1 | 0.6952 | NOS_BES | No | - | - | No |
| **ScshVNz_2264_127157** | 1 | 0.5489 | NOS_BES | No | - | - | No |
| **ScshVNz_2264_173646** | 1 | 0.5829 | NOS_BES | No | - | - | No |
| **ScshVNz_3821_798863** | 3 | 0.563 | NOS_BES | No | - | - | No |
| **ScshVNz_11228_35528705** | 4 | 0.8652 | NOS_PBS | No | - | - | No |
| **ScshVNz_11228_36872990** | 4 | 0.5329 | NOS_BES | No | - | - | No |
| **ScshVNz_1990_627646** | 4 | 0.935 | NOS_PBS | No | - | - | No |
| **ScshVNz_10772_14628715** | 5 | 0.9384 | NOS_PBS | No | - | - | No |
| **ScshVNz_6515_15405832** | 7 | 0.8448 | BES_PBS | No | - | - | No |
| **ScshVNz_10729_26462707** | 8 | 0.8347 | BES_PBS | No | - | - | No |
| **ScshVNz_10729_6463694** | 8 | 0.8542 | NOS_PBS | No | - | - | No |
| **ScshVNz_13313_6818230** | 11 | 0.5283 | NOS_BES | No | - | - | No |
| **ScshVNz_13313_9224097** | 11 | 0.8222 | NOS_PBS | No | - | - | No |
| **ScshVNz_12949_2368722** | 12 | 0.8652 | NOS_PBS | No | - | - | No |
| **ScshVNz_12150_6396196** | 15 | 0.8652 | NOS_PBS | No | - | - | No |
| **ScshVNz_12150_7032682** | 15 | 0.8334 | BES_PBS | No | - | - | No |
| **ScshVNz_9915_12500761** | 15 | 0.7202 | NOS_BES | No | - | - | No |
| **ScshVNz_1753_11483879** | 17 | 0.8652 | NOS_PBS | No | - | - | No |
| **ScshVNz_4138_2471492** | 17 | 0.7623 | NOS_BES | No | - | - | No |
| **ScshVNz_1065_10193851** | 18 | 0.8792 | NOS_PBS | No | - | - | No |
| **ScshVNz_1065_11282177** | 18 | 0.8538 | NOS_PBS | No | - | - | No |
| **ScshVNz_1065_18392032** | 18 | 0.6064 | NOS_BES | No | - | - | No |
| **ScshVNz_1065_5619737** | 18 | 0.8373 | BES_PBS | No | - | - | No |
| **ScshVNz_1065_8572951** | 18 | 0.849 | BES_PBS | No | - | - | No |
| **ScshVNz_11493_17803051** | 18 | 0.8652 | NOS_PBS | No | - | - | No |
| **ScshVNz_11493_17809375** | 18 | 0.8652 | NOS_PBS | No | - | - | No |
| **ScshVNz_11493_3920334** | 18 | 0.7787 | BES_PBS | No | - | - | No |
| **ScshVNz_10530_31337784** | Unplaced | 0.8381 | BES_PBS | No | - | - | No |
| **ScshVNz_12227_1721310** | Unplaced | 0.5306 | NOS_BES | No | - | - | No |
| **ScshVNz_12865_31328387** | Unplaced | 0.8373 | BES_PBS | No | - | - | No |
| **ScshVNz_9900_1547497** | Unplaced | 0.877 | BES_PBS | No | - | - | No |

**Table S5:** Estimated migration rates (per generation) and relative effective population sizes among populations. The two most likely scenarios are highlighted in green.

| **Name** | **Number of parameters estimated** | **Gene flow** | **NOS=>BES** | **BES=>NOS** | **BES=>PBS** | **PBS=>BES** | **NOS=>PBS** | **PBS=>NOS** | **Order of pop size estimates**  **(proportion in parenthesis)** |
| --- | --- | --- | --- | --- | --- | --- | --- | --- | --- |
| **3 populations, single split, colonization restricted to Baltic Sea period (≤ 8,500 years)** | | | | | | | | | |
| P1 | 1 | no | - | - | - | - | - | - | BES (0.62) > NOS (0.29) > PBS (0.09) |
| **3 populations, two splits, colonization restricted to Baltic Sea period (≤ 8,500 years); split order unconstrained** | | | | | | | | | |
| P2a | 2 | no | - | - | - | - | - | - | BES (0.94) > NOS (0.05) > PBS (0.01) |
| P6a | 6 | Yes, adjacent pops | <0.001 | 0.017 | 0.004 | <0.001 | - | - | NOS (0.55) > BES (0.30) > PBS (0.15) |
| P8a | 8 | Yes, all pops | <0.001 | <0.001 | 0.006 | 0.004 | 0.011 | 0.009 | BES (0.98) > NOS (0.01) > PBS (0.01) |
| **3 populations, two splits, colonization restricted to Baltic Sea period (≤ 8,500 years); 1st split NOS vs. (BES,PBS), 2nd split BES vs. PBS** | | | | | | | | | |
| P2b | 2 | no | - | - | - | - | - | - | BES (0.93) > NOS (0.05) > PBS (0.02) |
| P6b | 6 | Yes, adjacent pops | 0.002 | <0.001 | <0.001 | 0.028 | - | - | BES (0.98) > NOS (0.02) > PBS (0.0) |
| **3 populations, two splits, colonization allowed since onset of Yoldia period (≤ 12,000 years) ; split order unconstrained** | | | | | | | | | |
| P2c | 2 | no | - | - | - | - | - | - | NOS (0.51) > BES (0.32) > PBS (0.17) |
| P6c | 6 | Yes, adjacent pops | <0.001 | 0.016 | <0.001 | <0.001 | - | - | NOS (0.51) > BES (0.32) > PBS (0.17) |
| P8c | 8 | Yes, all pops | 0.002 | <0.001 | 0.003 | <0.001 | 0.005 | 0.012 | BES (0.98) > NOS (0.02) > PBS (0.00) |
| **3 populations, two splits; 1st split NOS vs. (BES,PBS) during Yoldia period, 2nd split BES vs. PBS during Baltic Sea period** | | | | | | | | | |
| P2d | 2 | no | - | - | - | - | - | - | BES (0.81) > NOS (0.13) > PBS (0.06) |
| P6d | 6 | Yes, adjacent pops | <0.001 | <0.001 | <0.001 | 0.024 | - | - | BES (0.92) > NOS (0.07) > PBS (0.01) |
| **3 populations, two splits; 1st split PBS vs. (NOS,BES) during Yoldia period, 2nd split NOS vs. BES during Baltic Sea period** | | | | | | | | | |
| P2e | 2 | no | - | - | - | - | - | - | BES (0.77) > NOS (0.16) > PBS (0.07) |
| P6e | 6 | Yes, adjacent pops | 0.001 | <0.001 | <0.001 | 0.026 | - | - | BES (0.96) > NOS (0.04) > PBS (0.00) |

**Table S6**: 78 putative adaptive SNPs. The SNP ID shows its scaffold and position. The reference/alternative allele, vaquita chromosomal location and the pairwise Fst values of NOS-BES, BES-PBS and NOS-PBS is also shown. ^*^ indicates the SNPs were also identified as selective sweeps.

| SNP ID | REF | ALT | Vaquita  Chr | Vaquita  Position | NOS-BES  Fst | BES-PBS  Fst | NOS-PBS  Fst |
| --- | --- | --- | --- | --- | --- | --- | --- |
| ScshVNz_2264_6830 | C | A | 1 | 254044 | 0.83 | 0.00 | 0.81 |
| ScshVNz_2264_26709 | A | G | 1 | 273751 | 0.83 | 0.00 | 0.82 |
| ScshVNz_2264_59096 | T | C | 1 | 302753 | 0.80 | 0.00 | 0.94 |
| ScshVNz_2264_81148 | G | A | 1 | 323598 | 0.66 | 0.00 | 0.79 |
| ScshVNz_2264_81149 | A | C | 1 | 323598 | 0.66 | 0.00 | 0.79 |
| ScshVNz_2264_99440 | G | T | 1 | 341739 | 0.66 | 0.00 | 0.60 |
| ScshVNz_2264_101811 | A | G | 1 | 343913 | 0.70 | 0.00 | 0.69 |
| ScshVNz_2264_104856 | T | C | 1 | 346828 | 0.69 | 0.00 | 0.54 |
| ScshVNz_2264_117251 | A | G | 1 | 358971 | 0.69 | 0.00 | 0.69 |
| ScshVNz_2264_153571^*^ | G | A | 1 | 398035 | 0.64 | 0.00 | 0.70 |
| ScshVNz_2264_173646 | G | A | 1 | 421370 | 0.58 | 0.00 | 0.52 |
| ScshVNz_2264_186730^*^ | C | A | 1 | 434121 | 0.58 | 0.00 | 0.52 |
| ScshVNz_2264_197765^*^ | C | G | 1 | 445895 | 0.57 | 0.00 | 0.52 |
| ScshVNz_2264_201718^*^ | A | G | 1 | 449953 | 0.57 | 0.00 | 0.47 |
| ScshVNz_2264_214765^*^ | A | G | 1 | 463281 | 0.59 | 0.00 | 0.52 |
| ScshVNz_2264_228365^*^ | G | A | 1 | 474799 | 0.65 | 0.00 | 0.69 |
| ScshVNz_2264_235089^*^ | G | A | 1 | 481326 | 0.65 | 0.05 | 0.44 |
| ScshVNz_2264_245725^*^ | A | C | 1 | 494080 | 0.50 | 0.01 | 0.30 |
| ScshVNz_2264_246499^*^ | A | G | 1 | 494714 | 0.59 | 0.00 | 0.52 |
| ScshVNz_2264_269694 | A | G | 1 | 520109 | 0.57 | 0.00 | 0.52 |
| ScshVNz_11693_6566207 | T | G | 1 | 139186373 | 0.50 | 0.58 | 0.00 |
| ScshVNz_11693_4787675 | C | G | 1 | 140955398 | 0.51 | 0.00 | 0.50 |
| ScshVNz_9900_4628120 | G | T | 1 | 159739619 | 0.50 | 0.00 | 0.00 |
| ScshVNz_3249_5258871 | C | T | 2 | 49797302 | 0.54 | 0.25 | 0.14 |
| ScshVNz_7569_31272050 | G | A | 2 | 138240566 | 0.53 | 0.36 | 0.05 |
| ScshVNz_3337_9384344 | A | G | 2 | 167652542 | 0.48 | 0.00 | 0.42 |
| ScshVNz_3821_798863 | T | C | 3 | 13990458 | 0.56 | 0.00 | 0.34 |
| ScshVNz_8211_4432446 | T | C | 3 | 28508293 | 0.49 | 0.00 | 0.30 |
| ScshVNz_9797_20846126 | C | T | 4 | 53458399 | 0.50 | 0.00 | 0.26 |
| ScshVNz_4462_18121874 | G | A | 5 | 18658290 | 0.46 | 0.00 | 0.00 |
| ScshVNz_4462_18128394 | T | C | 5 | 18664765 | 0.48 | 0.00 | 0.00 |
| ScshVNz_4462_18140824 | T | C | 5 | 18677172 | 0.49 | 0.00 | 0.00 |
| ScshVNz_4462_50015455 | A | G | 5 | 50666896 | 0.49 | 0.00 | 0.03 |
| ScshVNz_3795_1215616 | A | C | 5 | 78805562 | 0.00 | 0.93 | 0.87 |
| ScshVNz_10623_27589689 | T | C | 5 | 112677899 | 0.56 | 0.00 | 0.30 |
| ScshVNz_3409_2410987 | T | C | 6 | 23536628 | 0.52 | 0.00 | 0.44 |
| ScshVNz_880_9056514 | A | G | 6 | 28907162 | 0.47 | 0.00 | 0.33 |
| ScshVNz_6515_7960378 | T | G | 7 | 20530359 | 0.50 | 0.00 | 0.40 |
| ScshVNz_12227_1721552 | T | C | 7 | 98646939 | 0.46 | 0.00 | 0.61 |
| ScshVNz_12227_1721310 | T | C | 7 | 98647065 | 0.53 | 0.06 | 0.19 |
| ScshVNz_12227_1081147 | C | G | 7 | 99276564 | 0.49 | 0.00 | 0.69 |
| ScshVNz_9857_1841037 | A | G | 7 | 102252903 | 0.55 | 0.25 | 0.16 |
| ScshVNz_2531_27758442 | A | T | 9 | 54030151 | 0.48 | 0.00 | 0.00 |
| ScshVNz_2312_15326152 | G | T | 9 | 85377550 | 0.56 | 0.00 | 0.27 |
| ScshVNz_8015_13514346 | T | A | 10 | 25223350 | 0.56 | 0.00 | 0.00 |
| ScshVNz_8015_16045777 | T | G | 10 | 27715512 | 0.53 | 0.00 | 0.00 |
| ScshVNz_1183_6260023 | T | G | 10 | 98155402 | 0.48 | 0.00 | 0.29 |
| ScshVNz_12496_6643905 | G | A | 11 | 23344469 | 0.51 | 0.07 | 0.19 |
| ScshVNz_8212_987475 | C | T | 11 | 83558010 | 0.54 | 0.00 | 0.30 |
| ScshVNz_9860_37127380 | T | A | 12 | 31911477 | 0.52 | 0.01 | 0.08 |
| ScshVNz_9860_37128486 | C | T | 12 | 31912586 | 0.50 | 0.01 | 0.06 |
| ScshVNz_9860_59962032 | G | A | 12 | 54687688 | 0.47 | 0.00 | 0.10 |
| ScshVNz_12949_18961325 | T | C | 12 | 86783477 | 0.50 | 0.00 | 0.26 |
| ScshVNz_7385_21639734 | T | A | 13 | 23098218 | 0.49 | 0.00 | 0.00 |
| ScshVNz_7385_21529313 | G | A | 13 | 23210834 | 0.49 | 0.00 | 0.03 |
| ScshVNz_7385_18382361 | C | A | 13 | 26316639 | 0.55 | 0.00 | 0.10 |
| ScshVNz_3285_26006888 | T | C | 14 | 25883720 | 0.52 | 0.00 | 0.03 |
| ScshVNz_9915_12725861 | A | G | 15 | 18609353 | 0.73 | 0.00 | 0.73 |
| ScshVNz_9915_12638086^*^ | A | G | 15 | 18694785 | 0.50 | 0.01 | 0.52 |
| ScshVNz_9915_12564636^*^ | T | C | 15 | 18765089 | 0.59 | 0.01 | 0.62 |
| ScshVNz_9915_12513811 | A | C | 15 | 18814377 | 0.77 | 0.00 | 0.73 |
| ScshVNz_9915_12496798^*^ | T | C | 15 | 18832386 | 0.73 | 0.05 | 0.49 |
| ScshVNz_9915_4879279 | C | T | 15 | 26368618 | 0.53 | 0.06 | 0.21 |
| ScshVNz_10356_3076825 | A | G | 15 | 40976463 | 0.61 | 0.00 | 0.35 |
| ScshVNz_12150_31542967 | G | A | 15 | 57186619 | 0.50 | 0.00 | 0.00 |
| ScshVNz_12150_4946587 | A | G | 15 | 83372676 | 0.00 | 0.00 | 0.00 |
| ScshVNz_2616_8767703 | C | A | 16 | 2716565 | 0.56 | 0.01 | 0.64 |
| ScshVNz_982_19188785 | C | G | 16 | 36150569 | 0.54 | 0.01 | 0.08 |
| ScshVNz_7704_2124763 | T | A | 16 | 78223465 | 0.48 | 0.00 | 0.21 |
| ScshVNz_958_4025890 | A | G | 17 | 3998174 | 0.53 | 0.00 | 0.38 |
| ScshVNz_958_17064534 | A | G | 17 | 16927245 | 0.57 | 0.59 | 0.00 |
| ScshVNz_4138_2475757 | C | T | 17 | 50560865 | 0.68 | 0.00 | 0.50 |
| ScshVNz_4138_2471492 | G | A | 17 | 50565063 | 0.76 | 0.00 | 0.72 |
| ScshVNz_1065_5744809 | A | G | 18 | 57455155 | 0.50 | 0.58 | 0.00 |
| ScshVNz_2056_7007420 | G | A | 20 | 12732553 | 0.65 | 0.00 | 0.62 |
| ScshVNz_2056_2258207 | A | G | 20 | 17406339 | 0.55 | 0.00 | 0.30 |
| ScshVNz_9860_6162769 | G | A | 20 | 27299114 | 0.49 | 0.00 | 0.00 |
| ScshVNz_8323_9538827 | G | A | 20 | 49473502 | 0.54 | 0.24 | 0.16 |

**Table S7**: Curated BLAST hits of the 78 putative adaptive SNPs against the vaquita genome. Identity, length, region of the Vaquita genome and e-values are also shown.

| SNP ID | Identity | Length | Vaquita  Chr | Vaquita  Start | Vaquita  End | E-value |
| --- | --- | --- | --- | --- | --- | --- |
| ScshVNz_2264_6830 | 99.333 | 600 | 1 | 253744 | 254343 | 0 |
| ScshVNz_2264_26709 | 99.167 | 600 | 1 | 273451 | 274050 | 0 |
| ScshVNz_2264_59096 | 99.132 | 576 | 1 | 302453 | 303028 | 0 |
| ScshVNz_2264_81148 | 100 | 299 | 1 | 323298 | 323596 | 3.81E-155 |
| ScshVNz_2264_81149 | 100 | 300 | 1 | 323298 | 323597 | 1.06E-155 |
| ScshVNz_2264_99440 | 99.18 | 366 | 1 | 341439 | 341804 | 0 |
| ScshVNz_2264_101811 | 99.5 | 600 | 1 | 343613 | 344212 | 0 |
| ScshVNz_2264_104856 | 96.921 | 617 | 1 | 346528 | 347144 | 0 |
| ScshVNz_2264_117251 | 97.5 | 600 | 1 | 358671 | 359260 | 0 |
| ScshVNz_2264_153571 | 99.167 | 600 | 1 | 397735 | 398334 | 0 |
| ScshVNz_2264_173646 | 99.667 | 600 | 1 | 421070 | 421669 | 0 |
| ScshVNz_2264_186730 | 95.13 | 616 | 1 | 433821 | 434436 | 0 |
| ScshVNz_2264_197765 | 99 | 600 | 1 | 445595 | 446193 | 0 |
| ScshVNz_2264_201718 | 98.759 | 403 | 1 | 449653 | 450055 | 0 |
| ScshVNz_2264_214765 | 98.507 | 469 | 1 | 462981 | 463449 | 0 |
| ScshVNz_2264_228365 | 98.101 | 474 | 1 | 474499 | 474972 | 0 |
| ScshVNz_2264_235089 | 99.337 | 603 | 1 | 481026 | 481628 | 0 |
| ScshVNz_2264_245725 | 99.589 | 487 | 1 | 493780 | 494266 | 0 |
| ScshVNz_2264_246499 | 98.575 | 351 | 1 | 494414 | 494764 | 1.03E-175 |
| ScshVNz_2264_269694 | 99.667 | 600 | 1 | 519809 | 520408 | 0 |
| ScshVNz_11693_6566207 | 98.007 | 602 | 1 | 139186073 | 139185472 | 0 |
| ScshVNz_11693_4787675 | 99.833 | 600 | 1 | 140955098 | 140954499 | 0 |
| ScshVNz_9900_4628120 | 99.833 | 600 | 1 | 159739319 | 159739918 | 0 |
| ScshVNz_3249_5258871 | 99.08 | 435 | 2 | 49797002 | 49796569 | 0 |
| ScshVNz_7569_31272050 | 99.828 | 580 | 2 | 138240266 | 138240845 | 0 |
| ScshVNz_3337_9384344 | 99.833 | 600 | 2 | 167652242 | 167651643 | 0 |
| ScshVNz_3821_798863 | 96.494 | 542 | 3 | 13990158 | 13989633 | 0 |
| ScshVNz_8211_4432446 | 100 | 203 | 3 | 28507993 | 28507791 | 8.86E-102 |
| ScshVNz_9797_20846126 | 99.333 | 600 | 4 | 53458099 | 53457500 | 0 |
| ScshVNz_4462_18121874 | 99.511 | 409 | 5 | 18657990 | 18658398 | 0 |
| ScshVNz_4462_18128394 | 99.605 | 506 | 5 | 18664465 | 18664970 | 0 |
| ScshVNz_4462_18140824 | 98.701 | 385 | 5 | 18676872 | 18677256 | 0 |
| ScshVNz_4462_50015455 | 98.833 | 600 | 5 | 50666596 | 50667189 | 0 |
| ScshVNz_3795_1215616 | 99.333 | 600 | 5 | 78805262 | 78805861 | 0 |
| ScshVNz_10623_27589689 | 99.333 | 600 | 5 | 112677599 | 112677000 | 0 |
| ScshVNz_3409_2410987 | 99.5 | 600 | 6 | 23536328 | 23536927 | 0 |
| ScshVNz_880_9056514 | 99.231 | 130 | 6 | 28906862 | 28906733 | 1.57E-59 |
| ScshVNz_6515_7960378 | 99.667 | 600 | 7 | 20530059 | 20529460 | 0 |
| ScshVNz_12227_1721552 | 98.833 | 600 | 7 | 98646639 | 98646042 | 0 |
| ScshVNz_12227_1721310 | 99.587 | 484 | 7 | 98646765 | 98646282 | 0 |
| ScshVNz_12227_1081147 | 99.667 | 601 | 7 | 99276264 | 99275664 | 0 |
| ScshVNz_9857_1841037 | 99.5 | 600 | 7 | 102252603 | 102253202 | 0 |
| ScshVNz_2531_27758442 | 99.492 | 394 | 9 | 54029851 | 54029458 | 0 |
| ScshVNz_2312_15326152 | 100 | 230 | 9 | 85377250 | 85377021 | 8.67E-117 |
| ScshVNz_8015_13514346 | 99.203 | 502 | 10 | 25223050 | 25223551 | 0 |
| ScshVNz_8015_16045777 | 100 | 557 | 10 | 27715212 | 27715768 | 0 |
| ScshVNz_1183_6260023 | 100 | 600 | 10 | 98155102 | 98155701 | 0 |
| ScshVNz_12496_6643905 | 99.171 | 603 | 11 | 23344169 | 23343567 | 0 |
| ScshVNz_8212_987475 | 99.5 | 600 | 11 | 83557710 | 83558309 | 0 |
| ScshVNz_9860_37127380 | 99.5 | 600 | 12 | 31911177 | 31911776 | 0 |
| ScshVNz_9860_37128486 | 99.268 | 410 | 12 | 31912286 | 31912695 | 0 |
| ScshVNz_9860_59962032 | 100 | 327 | 12 | 54687388 | 54687714 | 1.04E-170 |
| ScshVNz_12949_18961325 | 99.667 | 600 | 12 | 86783177 | 86783776 | 0 |
| ScshVNz_7385_21639734 | 99.833 | 599 | 13 | 23097918 | 23097320 | 0 |
| ScshVNz_7385_21529313 | 99 | 600 | 13 | 23210534 | 23209935 | 0 |
| ScshVNz_7385_18382361 | 99.333 | 600 | 13 | 26316339 | 26315740 | 0 |
| ScshVNz_3285_26006888 | 99.679 | 312 | 14 | 25883420 | 25883731 | 1.05E-160 |
| ScshVNz_9915_12725861 | 99.833 | 600 | 15 | 18609053 | 18608454 | 0 |
| ScshVNz_9915_12638086 | 97.674 | 602 | 15 | 18694485 | 18693884 | 0 |
| ScshVNz_9915_12564636 | 100 | 600 | 15 | 18764789 | 18764190 | 0 |
| ScshVNz_9915_12513811 | 99.833 | 600 | 15 | 18814077 | 18813478 | 0 |
| ScshVNz_9915_12496798 | 99.5 | 600 | 15 | 18832086 | 18831487 | 0 |
| ScshVNz_9915_4879279 | 99.746 | 394 | 15 | 26368318 | 26367925 | 0 |
| ScshVNz_10356_3076825 | 99.167 | 600 | 15 | 40976163 | 40975564 | 0 |
| ScshVNz_12150_31542967 | 99.5 | 600 | 15 | 57186319 | 57185720 | 0 |
| ScshVNz_12150_4946587 | 99.318 | 440 | 15 | 83372376 | 83371937 | 0 |
| ScshVNz_2616_8767703 | 98.958 | 192 | 16 | 2716265 | 2716074 | 2.50E-92 |
| ScshVNz_982_19188785 | 99.235 | 523 | 16 | 36150269 | 36150790 | 0 |
| ScshVNz_7704_2124763 | 99.667 | 600 | 16 | 78223165 | 78223764 | 0 |
| ScshVNz_958_4025890 | 99.684 | 316 | 17 | 3997874 | 3998189 | 6.29E-163 |
| ScshVNz_958_17064534 | 99.296 | 426 | 17 | 16926945 | 16927368 | 0 |
| ScshVNz_4138_2475757 | 98.51 | 604 | 17 | 50560565 | 50559962 | 0 |
| ScshVNz_4138_2471492 | 98.344 | 604 | 17 | 50564763 | 50564163 | 0 |
| ScshVNz_1065_5744809 | 99.833 | 600 | 18 | 57454855 | 57455454 | 0 |
| ScshVNz_2056_7007420 | 100 | 595 | 20 | 12732253 | 12731659 | 0 |
| ScshVNz_2056_2258207 | 99.833 | 600 | 20 | 17406039 | 17405440 | 0 |
| ScshVNz_9860_6162769 | 99.169 | 602 | 20 | 27298814 | 27299415 | 0 |
| ScshVNz_8323_9538827 | 99.667 | 600 | 20 | 49473202 | 49472603 | 0 |

**Table S8:** Candidate genes identified within a 20 Kb distance of the 78 putative adaptive SNPs. Intragenic putative adaptive SNP IDs are shown for each respective gene.

| Gene | **Vaquita**  **Chr** | **Vaquita**  **Start** | **Vaquita**  **End** | **Intragenic SNPs** |
| --- | --- | --- | --- | --- |
| AGRN | 1 | 222091 | 254867 | ScshVNz_2264_6830 |
| PTPN11 | 1 | 259021 | 268641 | - |
| RNF223 | 1 | 269476 | 272257 | - |
| LOC116752387 | 1 | 273279 | 276335 | - |
| C1H1orf159 | 1 | 276237 | 286182 | - |
| TTLL10 | 1 | 362748 | 374791 | - |
| TNFRSF18 | 1 | 378323 | 381251 | - |
| TNFRSF4 | 1 | 385551 | 388831 | - |
| SDF4 | 1 | 391501 | 404099 | ScshVNz_2264_153571 |
| B3GALT6 | 1 | 404202 | 407047 | - |
| C1QTNF12 | 1 | 417083 | 421177 | ScshVNz_2264_173646 |
| UBE2J2 | 1 | 423339 | 442637 | ScshVNz_2264_186730 |
| SCNN1D | 1 | 442818 | 446178 | ScshVNz_2264_197765 |
| ACAP3 | 1 | 446633 | 460981 | ScshVNz_2264_201718 |
| PUSL1 | 1 | 460848 | 464531 | ScshVNz_2264_214765 |
| INTS11 | 1 | 464525 | 477833 | ScshVNz_2264_228365 |
| CPTP | 1 | 477915 | 481800 | ScshVNz_2264_235089 |
| TAS1R3 | 1 | 481947 | 486985 | - |
| DVL1 | 1 | 487128 | 499127 | ScshVNz_2264_245725, ScshVNz_2264_246499 |
| MXRA8 | 1 | 501976 | 506266 | No |
| AURKAIP1 | 1 | 510517 | 522859 | ScshVNz_2264_269694 |
| CCNL2 | 1 | 524513 | 532739 | - |
| MRPL20 | 1 | 535639 | 538288 | - |
| NMNAT2 | 1 | 139081408 | 139280792 | ScshVNz_11693_6566207 |
| SWT1 | 1 | 140933433 | 141024724 | ScshVNz_11693_4787675 |
| FAM169B | 2 | 49723234 | 49820354 | ScshVNz_3249_5258871 |
| PRIMA1 | 2 | 167585088 | 167645130 | - |
| ILVBL | 3 | 13973334 | 13983306 | - |
| SYDE1 | 3 | 13983306 | 13990169 | - |
| OR1\|1 | 3 | 14000756 | 14001835 | - |
| TENM2 | 3 | 28346067 | 29354191 | ScshVNz_8211_4432446 |
| NAALADL2 | 4 | 52438021 | 53631303 | ScshVNz_9797_20846126 |
| SPATA5 | 5 | 18471561 | 18816592 | ScshVNz_4462_18121874, ScshVNz_4462_18128394, ScshVNz_4462_18140824 |
| ARHGAP10 | 5 | 112413515 | 112744654 | ScshVNz_10623_27589689 |
| PALM2AKAP2 | 6 | 23329378 | 23826234 | ScshVNz_3409_2410987 |
| GPR39 | 7 | 98527325 | 98768064 | ScshVNz_12227_1721310, ScshVNz_12227_1721552 |
| RPLP1 | 7 | 98538907 | 98648723 | - |
| DDX18 | 7 | 102230202 | 102247358 | - |
| MAGI2 | 9 | 84589307 | 85936364 | ScshVNz_2312_15326152 |
| KCNA1 | 10 | 98152893 | 98154583 | - |
| CARMIL1 | 11 | 83455895 | 83779624 | ScshVNz_8212_987475 |
| ALK | 13 | 22968531 | 23711026 | ScshVNz_7385_21529313, ScshVNz_7385_21639734 |
| SMAD2 | 14 | 25800049 | 25909443 | ScshVNz_3285_26006888 |
| CHD6 | 15 | 18487167 | 18700992 | ScshVNz_9915_12638086, ScshVNz_9915_12725861 |
| LOC116739994 | 15 | 18687536 | 18696566 | - |
| LPIN3 | 15 | 18738119 | 18755410 | - |
| ZHX3 | 15 | 18775284 | 18898847 | ScshVNz_9915_12496798, ScshVNz_9915_12513811 |
| ASXL1 | 15 | 26304337 | 26372939 | ScshVNz_9915_4879279 |
| MACROD2 | 15 | 40366977 | 42426128 | - |
| USP7 | 15 | 57170670 | 57228466 | ScshVNz_12150_31542967 |
| PMS2 | 15 | 83331474 | 83356209 | - |
| AIMP2 | 15 | 83356342 | 83364475 | - |
| EIF2AK1 | 15 | 83364473 | 83395844 | ScshVNz_12150_4946587 |
| ANKRD61 | 15 | 83369728 | 83374140 | - |
| WAPL | 16 | 36100249 | 36177872 | ScshVNz_982_19188785 |
| KCNB2 | 17 | 16844867 | 17235822 | ScshVNz_958_17064534 |
| SMG6 | 20 | 12593180 | 12828054 | ScshVNz_2056_7007420 |
| DNAH17 | 20 | 49334128 | 49497240 | ScshVNz_8323_9538827 |
| PGS1 | 20 | 49479792 | 49535023 | - |

**Table S9:** Significantly enriched Gene Ontology (GO) terms with Fisher’s exact test Pvalue < 0.05 in candidate genes.

| Category | Term | GO terms | Genes | P-value |
| --- | --- | --- | --- | --- |
| Biological Process | GO:0051128 | Regulation of cellular component organization | ALK, CARMIL1, USP7, TENM2, EIF2AK1, MAGI2, PTPN11, WAPL, SMG6, SYDE1, ACAP3, CPTP, DVL1, CCNL2, AGRN, AURKAIP1, ANKRD61 | 0.0004 |
| Biological Process | GO:0031346 | Positive regulation of cell projection organization | ALK, CARMIL1, TENM2, MAGI2, DVL1, AGRN | 0.0026 |
| Molecular Function | GO:0140640 | Catalytic activity, acting on a nucleic acid | INTS11, DDX18, CHD6, PMS2, PUSL1, WAPL, SMG6 | 0.0067 |
| Biological Process | GO:0120035 | Regulation of plasma membrane bounded cell projection organization | ALK, CARMIL1, ACAP3, TENM2, MAGI2, DVL1, AGRN | 0.0073 |
| Biological Process | GO:0043113 | Receptor clustering | MAGI2, DVL1, AGRN | 0.0081 |
| Biological Process | GO:0031344 | Regulation of cell projection organization | ALK, CARMIL1, ACAP3, TENM2, MAGI2, DVL1, AGRN | 0.0082 |
| Biological Process | GO:0080090 | Regulation of primary metabolic process | ALK, SMAD2, INTS11, USP7, TENM2, ZHX3, TNFRSF18, EIF2AK1, MAGI2, CHD6, PTPN11, NMNAT2, WAPL, SMG6, ASXL1, AIMP2, C1QTNF12, DVL1, PMS2, CCNL2, AGRN, AURKAIP1, TNFRSF4, LPIN3 | 0.0150 |
| Molecular Function | GO:0005524 | ATP binding | PGS1, ALK, DDX18, DNAH17, TTLL10, EIF2AK1, CHD6, PMS2, NMNAT2, UBE2J2 | 0.0151 |
| Biological Process | GO:0009987 | Cellular process | ALK, INTS11, TENM2, TTLL10, KCNA1, RPLP1, CHD6, UBE2J2, PALM2AKAP2, SMG6, PGS1, MRPL20, SYDE1, DVL1, SDF4, PMS2, MACROD2, PUSL1, TAS1R3, TNFRSF4, SMAD2, GPR39, CARMIL1, DDX18, USP7, ZHX3, DNAH17, TNFRSF18, EIF2AK1, KCNB2, MAGI2, B3GALT6, PTPN11, NMNAT2, WAPL, ASXL1, AIMP2, ARHGAP10, ACAP3, SCNN1D, ILVBL, CPTP, C1QTNF12, MXRA8, AURKAIP1, AGRN, ANKRD61, LPIN3 | 0.0159 |
| Molecular Function | GO:0032559 | Adenyl ribonucleotide binding | PGS1, ALK, DDX18, DNAH17, TTLL10, EIF2AK1, CHD6, PMS2, NMNAT2, UBE2J2 | 0.0192 |
| Biological Process | GO:0048731 | System development | ALK, SMAD2, TENM2, TTLL10, KCNA1, MAGI2, PTPN11, NMNAT2, ASXL1, ACAP3, AIMP2, DVL1, MXRA8, SDF4, PMS2, MACROD2, AGRN | 0.0202 |
| Molecular Function | GO:0043168 | Anion binding | ALK, DDX18, DNAH17, TTLL10, EIF2AK1, CHD6, NMNAT2, UBE2J2, PGS1, ILVBL, CPTP, PMS2, AGRN | 0.0232 |
| Cellular Component | GO:0016020 | Membrane | ALK, TENM2, TTLL10, KCNA1, UBE2J2, PALM2AKAP2, PGS1, MRPL20, SYDE1, DVL1, SDF4, TAS1R3, TNFRSF4, PRIMA1, SMAD2, GPR39, CARMIL1, DDX18, TNFRSF18, KCNB2, MAGI2, B3GALT6, NMNAT2, NAALADL2, RNF223, AIMP2, ARHGAP10, ACAP3, SCNN1D, ILVBL, CPTP, MXRA8, AURKAIP1, AGRN, LPIN3 | 0.0235 |
| Biological Process | GO:2000812 | Regulation of barbed-end actin filament capping | CARMIL1, ANKRD61 | 0.0253 |
| Molecular Function | GO:0005031 | Tumor necrosis factor receptor activity | TNFRSF18, TNFRSF4 | 0.0253 |
| Biological Process | GO:0022008 | Neurogenesis | ALK, ACAP3, TENM2, KCNA1, DVL1, MXRA8, PTPN11, NMNAT2, AGRN | 0.0267 |
| Molecular Function | GO:0030554 | Adenyl nucleotide binding | PGS1, ALK, DDX18, DNAH17, TTLL10, EIF2AK1, CHD6, PMS2, NMNAT2, UBE2J2 | 0.0270 |
| Biological Process | GO:0007399 | Nervous system development | ALK, ACAP3, TENM2, KCNA1, MAGI2, DVL1, MXRA8, SDF4, PTPN11, NMNAT2, MACROD2, AGRN | 0.0274 |
| Biological Process | GO:0006639 | Acylglycerol metabolic process | PGS1, PTPN11, LPIN3 | 0.0291 |
| Biological Process | GO:0006638 | Neutral lipid metabolic process | PGS1, PTPN11, LPIN3 | 0.0296 |
| Biological Process | GO:0048666 | Neuron development | ALK, TENM2, KCNA1, DVL1, PTPN11, NMNAT2, AGRN | 0.0305 |
| Biological Process | GO:0061564 | Axon development | TENM2, KCNA1, DVL1, PTPN11, NMNAT2 | 0.0323 |
| Molecular Function | GO:0005035 | Death receptor activity | TNFRSF18, TNFRSF4 | 0.0328 |
| Cellular Component | GO:0120025 | Plasma membrane bounded cell projection | CARMIL1, ACAP3, TENM2, DNAH17, TTLL10, KCNB2, KCNA1, MAGI2, DVL1, MXRA8, SDF4, NMNAT2 | 0.0353 |
| Biological Process | GO:0048699 | Generation of neurons | ALK, ACAP3, TENM2, KCNA1, DVL1, PTPN11, NMNAT2, AGRN | 0.0360 |
| Cellular Component | GO:0150034 | Distal axon | ACAP3, TENM2, KCNA1, DVL1 | 0.0372 |
| Biological Process | GO:0072015 | Podocyte development | ASXL1, MAGI2 | 0.0377 |
| Biological Process | GO:0033043 | Regulation of organelle organization | SYDE1, CARMIL1, USP7, EIF2AK1, AURKAIP1, ANKRD61, WAPL, SMG6 | 0.0391 |
| Biological Process | GO:0072310 | Glomerular epithelial cell development | ASXL1, MAGI2 | 0.0402 |
| Cellular Component | GO:0032991 | Protein-containing complex | ALK, INTS11, KCNA1, RPLP1, CHD6, UBE2J2, SMG6, MRPL20, DVL1, PMS2, CCNL2, TAS1R3, SMAD2, CARMIL1, USP7, ZHX3, DNAH17, KCNB2, MAGI2, PTPN11, WAPL, ASXL1, AIMP2, SCNN1D, ILVBL, AURKAIP1 | 0.0445 |
| Molecular Function | GO:0008094 | ATP-dependent activity, acting on DNA | CHD6, PMS2, WAPL | 0.0450 |
| Cellular Component | GO:0042995 | Cell projection | CARMIL1, ACAP3, TENM2, DNAH17, TTLL10, KCNB2, KCNA1, MAGI2, DVL1, MXRA8, SDF4, NMNAT2 | 0.0477 |
| Biological Process | GO:0045721 | Negative regulation of gluconeogenesis | USP7, C1QTNF12 | 0.0499 |

**Table S10:** Genes and putative adaptive SNPs also identified as selective sweeps of the divergent region located in Pseudochromosome 1, Scaffold_2264- 0-280,000.

| Vaquita Chr | Vaquita Start | Vaquita End | Orientation | Gene Symbol / SNP |
| --- | --- | --- | --- | --- |
| 1 | 222091 | 254867 | plus | **AGRN** |
| 1 | 259021 | 268641 | plus | **PTPN11** |
| 1 | 269476 | 272257 | minus | **RNF223** |
| 1 | 276237 | 286182 | minus | **C1H1orf159** |
| 1 | 362748 | 374791 | plus | **TTLL10** |
| 1 | 378323 | 381251 | minus | **TNFRSF18** |
| 1 | 385551 | 388831 | minus | **TNFRSF4** |
| 1 | 391501 | 404099 | minus | **SDF4** |
| 1 | 397735 | - | - | ScshVNz_2264_153571 |
| 1 | 404202 | 407047 | plus | **B3GALT6** |
| 1 | 417083 | 421177 | minus | **C1QTNF12** |
| 1 | 423339 | 442637 | minus | **UBE2J2** |
| 1 | 433821 | - | - | ScshVNz_2264_186730 |
| 1 | 442818 | 446178 | plus | **SCNN1D** |
| 1 | 445595 | - | - | ScshVNz_2264_197765 |
| 1 | 446633 | 460981 | minus | **ACAP3** |
| 1 | 449653 | - | - | ScshVNz_2264_201718 |
| 1 | 460848 | 464531 | plus | **INTS11** |
| 1 | 462981 | - | - | ScshVNz_2264_214765 |
| 1 | 464525 | 477833 | minus | **PUSL1** |
| 1 | 474499 | - | - | ScshVNz_2264_228365 |
| 1 | 477915 | 481800 | plus | **CPTP** |
| 1 | 481026 | - | - | ScshVNz_2264_235089 |
| 1 | 481947 | 486985 | plus | **TAS1R3** |
| 1 | 487128 | 499127 | minus | **DVL1** |
| 1 | 493780 | - | - | ScshVNz_2264_245725 |
| 1 | 494414 | - | - | ScshVNz_2264_246499 |
| 1 | 501976 | 506266 | minus | **MXRA8** |
| 1 | 510517 | 522859 | minus | **AURKAIP1** |
| 1 | 524513 | 532739 | minus | **CCNL2** |

**Table S11:** Genes and putative adaptive SNPs also identified as selective sweeps of the divergent region located in Pseudochromosome 15, Scaffold_9915-12,250,000-12,800,000.

| Vaquita Chr | Vaquita Start | Vaquita End | Orientation | Gene Symbol / SNP |
| --- | --- | --- | --- | --- |
| 15 | 18487167 | 18700992 | plus | **CHD6** |
| 15 | 18694485 | - | - | ScshVNz_9915_12638086 |
| 15 | 18732406 | 18738024 | plus | **EMILIN3** |
| 15 | 18738119 | 18755410 | minus | **LPIN3** |
| 15 | 18775284 | 18898847 | plus | **ZHX3** |
| 15 | 18832086 | - | - | ScshVNz_9915_12496798 |
| 15 | 18902109 | 18937684 | minus | **PLCG1** |
| 15 | 18947302 | 19033328 | minus | **TOP1** |

**Table S12:** Assigned populations and sampling region for each of the 273 reliably assigned Harbour porpoises discovered between May to October.

|  | **Assigned NOS** | **Assigned BES** | **Assigned PBS** |  |
| --- | --- | --- | --- | --- |
| **NOS region** | 32 | 22 | 0 | **54** |
| **BES region** | 23 | 174 | 3 | **200** |
| **PBS region** | 6 | 10 | 3 | **19** |
|  | **61** | **206** | **6** | 273 |

**Table S13:** Assigned populations and sampling region for each of the 78 reliably assigned Harbour porpoises bycaught between May to October.

|  | **Assigned NOS** | **Assigned BES** | **Assigned PBS** |  |
| --- | --- | --- | --- | --- |
| **NOS region** | 16 | 11 | 0 | **27** |
| **BES region** | 6 | 26 | 1 | **33** |
| **PBS region** | 5 | 10 | 3 | **18** |
|  | **27** | **47** | **4** | 78 |

**Table S14:** Sensitivity analysis (10 replicates) on the effect of different mutation rates (per site per generation) on the estimated divergence times and effective population sizes (Ne) for the two most likely scenarios 6a and 6c. The values for the original 100 replicates are provided for comparison. The coefficients of variation (CV) show that altered mutation rates do not substantially change estimates for divergence times (around 1% for 6a; 2-15% for 6c). However, Ne estimates are more sensitive to altered mutation rates (CV 8-19% for 6a; 16-148% for 6c), driven largely by a lack of convergence in one of the ten 6c 1.44x10^-8^ replicates.

| **Scenario** | **Mutation rate** | **No replicates** | **Divergence time BES (yBP)** | **Divergence time PBS (yBP)** | **Ne NOS** | **Ne BES** | **Ne PBS** |
| --- | --- | --- | --- | --- | --- | --- | --- |
| **6a** | 2.56x10^-8^ | 100 | 8,044 | 5,510 | 28,287 | 15,238 | 7,918 |
| **6a** | 2.56x10^-8^ | 10 | 8,294 | 5,855 | 32,489 | 19,854 | 8,418 |
| **6a** | 1.44x10^-8^ | 10 | 8,366 | 5,926 | 25,242 | 21,480 | 9,682 |
| **6a** | 1.08x10^-8^ | 10 | 8,223 | 5,760 | 22,891 | 18,086 | 8,635 |
| **CV** | among | 10 | 0.009 | 0.014 | 0.186 | 0.086 | 0.076 |
| **6c** | 2.56x10^-8^ | 100 | 11,805 | 7,854 | 35,313 | 22,165 | 11,884 |
| **6c** | 2.56x10^-8^ | 10 | 11,484 | 8,782 | 39,709 | 28,486 | 12,529 |
| **6c** | 1.44x10^-8^ | 10 | 11,234 | 6,474 | 600,466 | 22,366 | 5,411 |
| **6c** | 1.08x10^-8^ | 10 | 11,745 | 7,747 | 25,242 | 21,480 | 9,682 |
| **CV** | among | 10 | 0.022 | 0.151 | 1.479 | 0.158 | 0.389 |

**Figure S1:** Postglacial evolution of the Baltic Sea basin after retreat of the Fennoscandian ice sheet: (a) Baltic Ice Lake (~14,000–11,700 BP), freshwater, ice-dammed; (b) Yoldia Sea (~11,700–10,700 BP), brief marine/brackish phase; (c) Ancylus Lake (~10,700–9,800 BP), freshwater following isostatic uplift; (d) modern brackish Baltic Sea, established from ~8,000 BP as the Danish straits opened. Present-day coastlines and major bordering cities shown for reference. Maps courtesy of GEOMAR Kiel, derived from Koyos and Juschki (CC-BY-SA 4.0).

**Figure S2:** Proportion of chromosomes lengths in final scaffold mapping between the harbour porpoise (HP) and the vaquita.

**Figure S3:** Bioinformatic pipeline.

**Figure S4:** Demographic scenarios tested in *fastsimcoal2*. Thirteen scenarios of increasing complexity representing different models of population divergence and connectivity among the North Sea (NOS), Belt Sea (BES), and Proper Baltic Sea (PBS) harbour porpoise populations. Scenario P1 assumes a simultaneous split of all three populations. P2 scenarios assume two sequential splits without gene flow, differing in the order of divergence and the permitted time window for colonization: scenarios restricted to the Baltic Sea period (P2a, P2b) allow splits from 8,500 years before present (yBP) to present, while those extending into the Yoldia Sea period (P2c, P2d, P2e) allow splits from up to 12,000 yBP. P6 scenarios mirror the corresponding P2 topologies, but additionally incorporate asymmetric gene flow between adjacent populations (dashed arrows). P8 scenarios similarly mirror their P2 counterparts, but allow asymmetric gene flow among all three populations. Time ranges (in yBP) indicate the permitted intervals for each divergence event. NOS was designated as the ancestral population in all scenarios.

P1

P2a

P2c

**P6a**

**P6c**

P8a

P8c

P2b

P6b

P2d

P6d

P2e

P6e

**1 Parameter**

(one split)

**2 Parameters**

(two splits)

**6 Parameters**

(two splits and four migration rates)

**8 Parameters**

(two splits and six migration rates)

**Split order**

**unconstrained**

**Split order**

**constrained**

**Split order**

**constrained**

**Baltic Sea period**

**only**

**Baltic Sea & Yoldia**

**period**

**Figure S5:** Graphical visualization of testing scheme among the 13 evaluated scenarios. Note that only the scenarios combined with arrows were directly compared (arrow towards favoured scenario), corresponding to a strictly orthogonal testing scheme to maintain the set experiment wise error of α=0.01. Overall favoured scenarios are marked in green.

**Figure S6:** Admixture analysis of Harbour porpoises (N = 56), K2. Colors represent genetic clusters: Dark blue Belt Sea population, light blue Inner Baltic Sea population. Delta K method showing K3 as the highest likelihood

**Figure S7:** Maps showing the assignment and location of the harbour porpoise samples sequenced at the whole genome level. Samples are colored according to their genetic ancestry under K3: North Sea (NOS) red, Belt Sea (BES) dark blue, Admixed purple, Proper Baltic Sea (PBS) light blue.

**Figure S8:** Likelihood distributions across demographic scenarios tested in fastsimcoal2. Box plots show the distribution of log-likelihoods across 100 independent runs of 1,000,000 coalescent simulations for each scenario. Scenarios are grouped by colonization time window of the first split: scenarios with suffixes "a" and "b" were restricted to the Baltic Sea period (<8,500 years BP), and those with suffix "c", "d", and "e" extended into the Yoldia Sea period (<12,000 years BP). Within each group, scenarios increase in complexity from left to right, with higher-numbered scenarios incorporating gene flow among adjacent (P6) or all populations (P8). Higher (less negative) likelihood values indicate better-supported models. Box plots show the median (centre line), interquartile range (box), and full range of likelihoods (whiskers) across the 100 runs. Note that non-overlapping likelihood ranges between scenarios were used as the criterion for model comparison, maintaining an experiment wise error rate of α = 0.01.

***Fst***

***dxy***

***π***

***NOS***

***PBS***

***Fst***

***π***

***dxy***

***π***

***Fst***

***dxy***

**Figure S9:** NOS-PBS Fst, dxy and π statistic values and correlations. Red dots represent outlier windows (0.05% top Fst).

***NOS***

***BES***

***PBS***

**Figure S10:** Within population diversity (π) values calculated with pixy

**Figure S11:** NOS-PBS XP_EHH values. Positive XP_EHH scores suggest recent positive selection in PBS, while negative one's positive selection in NOS.

**Figure S12:** The 376 Harbour porpoise samples for which the SNP panel was genotyped with microfluidic arrays. Samples are colored according to location: North Sea (NOS) red, Belt Sea (BES) dark blue, Belt Sea – Proper Baltic Sea transition zone (BES-PBS) purple, Proper Baltic Sea (PBS) light blue. The dotted lines represent the population boundaries identify by Carlén et al 2018 and Sveegard et al 2015.

**Figure S13**: Evaluation of power of assignment of the SNP panel. (a) Principal Component Analysis (PCA) of baseline samples (N = 47) showing the first and second PCs, using the 192 SNPs of the SNP panel. The colors represent the genetic clusters: Red Atlantic cluster, dark blue Belt Sea population, light blue Proper Baltic Sea population. (b) Admixture Analysis of harbour porpoises (N = 47), assuming 3 populations. (c) Assignment accuracies estimated with *assingpop* R package with three levels of baseline individuals (50%, 70% and 90% of individuals from each group) and four levels of training SNPs (top 10%, 25%, 50% and all SNPs) by 100 resampling events. From left to right: all populations combined, BES, NOS and PBS populations analyzed separately.

**Figure S14:** The 273 Harbour porpoise samples which were discovered in the period when the Baltic populations are considered to be geographically separated (May to October) and that were genotyped with microfluidic arrays. Colors represent the genetic clusters: Red Atlantic cluster, dark blue Belt Sea population, light blue Proper Baltic Sea population. The dotted lines represent the population boundaries identify by Carlén et al 2018 and Sveegard et al 2015.

**Figure S15:** The 78 Harbour porpoise samples which were bycaught in the period when the Baltic populations are considered to be geographically separated (May to October) and that were genotyped with microfluidic arrays. Colors represent the genetic clusters: Red Atlantic cluster, dark blue Belt Sea population, light blue Proper Baltic Sea population. The dotted lines represent the population boundaries identify by Carlén et al 2018 and Sveegard et al 2015.

**P6a_2.56**

**P6c_2.56**

**P6a_1.08**

**P6a_1.44**

**P6c_1.08**

**P6c_1.08**

**Figure S16:** Likelihood distributions for the two best-supported scenarios (6a and 6c) under three different mutation rates. Box plots show the distribution of log-likelihoods across 10 independent replicate runs of 1,000,000 coalescent simulations for each scenario-mutation rate combination, using the mutation rate adopted in the main analyses (2.56 × 10⁻⁸ substitutions/site/generation; P4a, P4c) and two alternative rates (1.08 × 10⁻⁸: P4a_108, P4c_108; 1.44 × 10⁻⁸: P4a_144, P4c_144). Box plots show the median (center line), interquartile range (box), and full range of likelihoods (whiskers) across the 10 replicates.
